# Checklist of the Coleoptera of Mordovia State Nature Reserve, Russia

**DOI:** 10.3897/zookeys.962.54477

**Published:** 2020-08-20

**Authors:** Leonid V. Egorov, Alexander B. Ruchin, Viktor B. Semenov, Oleg I. Semionenkov, Gennady B. Semishin

**Affiliations:** 1 The State Nature Reserve «Prisursky», Lesnoj, 9, 428034 Cheboksary, Russia; 2 Joint Directorate of the Mordovia State Nature Reserve and National Park «Smolny», Krasnaya str., 30, 430005 Saransk, Russia; 3 National Park «Smolensk Lakeland», Gurevich str., 19, Przhevalskoye, Demidov district, 216270 Smolensk region, Russia

**Keywords:** Biodiversity, beetles, Coleoptera checklist, eastern Europe, Republic of Mordovia

## Abstract

All 2,145 species of Coleoptera from 88 families known to occur in Mordovia State Nature Reserve, Russia, are listed, along with their author(s) and year of description using the most recent classification framework. Adventive species for European Russia are indicated. There are 31 adventive species in the reserve, comprising 1.44% of the total beetle fauna.

## Introduction

Rapid environmental changes due to urbanization and climate change have recently had a major impact on biodiversity (Czech et al. 2000, Kottawa-Arachchi and Wijeratne 2017, Rozhnov et al. 2019, Zamotajlov et al. 2019). In particular, the application of modern technologies in agriculture, ongoing deforestation, and changes in land use due to urbanization, cause the loss of biodiversity (Myers and Knoll 2001, Novacek and Cleland 2001, Lambin et al. 2003, Kestemont 2019). That is why the value of protected area (nature reserves and national parks) is steadily increasing. Moreover, in order to preserve biodiversity, it is necessary to identify key factors determining the distribution of species in their habitats. Such studies can be carried out in territories that were little affected by human activity; these territories are called protected area (Basset et al. 2007, Grebennikov 2016).

The term biodiversity hotspot is commonly used for regions or areas with high species richness, genetic richness, evolutionary important areas of origin, etc. (Reid 1998, Médail and Quézel 1999, Zagmajster et al. 2008, Silva and Ferreira 2016, Kumar et al. 2020). At the same time, in the most developed areas, such biodiversity hotspots are protected areas. Protected areas usually occupy certain areas in natural and climatic zones and include typical ecosystems of such climatic zones. In the forest natural zone, such areas are sparsely touched woodlands, these are different types of steppe areas in steppe. Currently global Protected Area Network covers approximately 14.9% of the world’s terrestrial land surface (Belle et al. 2018). Inventorying all biota is the best way to study biodiversity in the area (Weibull et al. 2003, Grebennikov 2016, Negrobov et al. 2018). However, such studies may often not be carried out due to limitations in the field of human resources and, therefore, certain insect families or ecological insect groups that are bioindicators are more often used (Lindenmayer et al. 2006, Lachat et al. 2012, Pozsgai and Littlewood 2014, Polevoi et al. 2018, Prokin et al. 2019; Ruchin et al. 2019a). On the other hand, the faunal analysis of individual insect orders can be carried out for a certain time; the data generated can then be used to compile a checklist of species and further analyze the spread of species, their distribution in the territory, settlement routes, etc. This can only be done if the most diverse methods covering all ecological groups of insects are applied (Basset et al. 2007); hence this checklist of the Coleoptera (Insecta) of Mordovia State Nature Reserve, based on a variety of methods.

**Figure 1.**
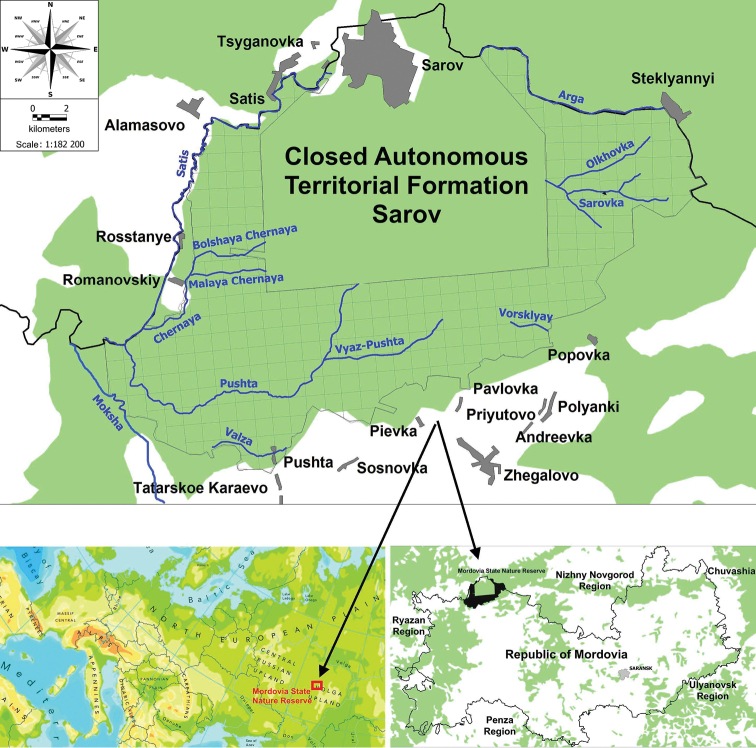
Location map of the Mordovia State Nature Reserve.

The Mordovia State Nature Reserve was established in 1936. It is located in the Temnikov district of the Republic of Mordovia (European Russia) on the forested right bank of the Moksha River and covers an area of 321.62 km^2^ (Fig. 1). From the north, the border runs along the Satis River (the right tributary of the Moksha), further to the east along the Arga River, which flows into the Satis River. The western border runs along the Chernaya, Satis, and Moksha rivers. From the south, the forest-steppe approaches naturally delineating the boundary of the reserve massif. By natural zoning, the forest tract of the Mordovia State Nature Reserve belongs to the zone of coniferous-deciduous forests on the border with the forest-steppe. Forest communities occupy 89.3% of the total territory (Ruchin and Egorov 2017b). In general, the vegetation cover of the Mordovia State Nature Reserve has a taiga character with tendency towards a nemoral (broad-leaved) forest type during successions. The intermix of forest-steppe elements is typical for this territory. *Pinus sylvestris* L. is the dominant forest tree in the Mordovia State Nature Reserve. It forms pure or mixed communities in the southern, central, and western parts of the reserve. *Betula pendula* Roth stands are the second largest forest type in the reserve. These are predominantly secondary communities at the sites of cut and burnt pine forests. Especially, many young birch stands developed at places damaged by the wildfire in 2010. *Tilia cordata* Mill. stands are located mainly in the northern part of the Mordovia State Nature Reserve. These are also secondary plant communities that arose on the site of pine forests and lime-spruce forests. *Quercus robur* L. forests occupy a relatively small area of the Mordovia State Nature Reserve. They are common in the Moksha River floodplain in the western part of the reserve. *Picea abies* L. and *Alnus glutinosa* (L.) Gaertn. stands are located mainly in floodplains of rivers and streams (Pushta, Vyaz-Pushta, Vorsklyai, Arga, etc.) and occupy small areas (Stojko and Senkevich 2018). Plant communities of small-leaved tree species (birch, aspen, alder) are formed in burnt forest areas (Khapugin et al. 2016, Vargot 2016, Khapugin and Ruchin 2019). The main areas of floodplain meadows are located along the Moksha River in the south-west of the Mordovia State Nature Reserve.

Previously, studies were carried out for individual orders and families of the arthropod fauna of the Mordovia State Nature Reserve, in particular for the Neuroptera and Raphidioptera (Ruchin and Makarkin 2017, Makarkin and Ruchin 2019), Hymenoptera (Ruchin and Antropov 2019), Orthoptera (Ruchin and Mikhailenko 2018) and Diptera (Chursina and Ruchin 2018a, 2018b, Astakhov et al. 2019).

## Materials and methods

The work is based on the study of authors’ own collections and analysis of published data. Two publications (Redikortsev 1938, Plavilshchikov 1964) were the first to describe the beetle fauna of Mordovia State Nature Reserve. In the late 1960s, xylophagous insects were studied in this area (Mozolevskaya et al. 1971, Kirsta 1974), and in the 1970s Carabidae were investigated (Feoktistov 1978, Feoktistov and Dushenkov 1982). Besides these groups, the Coleoptera fauna was hardly studied until the 2000s. More intensive research has been carried out using a variety of methods in the reserve over the past 12 years (Kurmaeva et al. 2008, Egorov et al. 2010, Feoktistov 2011, Egorov and Ruchin 2012, 2013a, 2013b, 2014, Pavlov and Ruchin 2013, Legalov et al. 2014, Egorov et al. 2015, 2016, 2017, 2018, 2019, 2020, Egorov and Semishin 2016, Egorov 2017, Sazhnev 2017, Ruchin and Egorov 2018a, 2018b, 2018d, 2018e, 2019b, Tomaszewska et al. 2018, Kazantsev et al. 2019, Ruchin et al. 2019b, 2019c, Egorov and Ruchin 2020). There are published data on individual families: Carabidae (Feoktistov 2008, Ruchin et al. 2016), Staphylinidae (Semenov 2014, Kurbatov and Egorov 2014, Semenov 2015, 2016, 2017), Scydmaenidae (Kurbatov and Egorov 2014), Curculionidae (Scolytinae) (Mandelshtam and Egorov 2017, 2018, 2019). Data on some species from the reserve are contained in faunal articles on different taxa (Ruchin and Egorov 2007, Ruchin et al. 2009, Egorov and Shapovalov 2017, Ruchin and Egorov 2017a) and in reviews of some Coleoptera families (Ruchin et al. 2013, Kovalev and Egorov 2017, Ruchin and Egorov 2018c, Ruchin et al. 2018, Ruchin and Egorov 2019a, Ruchin et al. 2019d, Zemoglyadchuk et al. 2020) of the Republic of Mordovia.

The research material was collected by the authors and their colleagues from 2008 to 2019. More than 70,000 samples were studied. Most of the Coleoptera species is stored in the collection of the Mordovia State Nature Reserve (Pushta, Republic of Mordovia) (indicated in our publications), Staphylinidae is stored in the collection of O.I. Semionenkov (Smolensk), Pselaphinae and Scydmaenidae are stored in the collection of S.A. Kurbatov (Moscow), Mordellidae is in the collection of A.V. Zemoglyadchuk (Baranovichi). Several species were transferred to the Zoological Institute of the Russian Academy of Sciences, St. Petersburg (ZIN), and to the Zoological Museum of Lomonosov Moscow State University, Moscow (ZMMU). Some species are stored in the personal collection of S.K. Alekseev (Kaluga).

In order to collect a representative material, the entire range of entomological field research methods were used included various traps such as pitfall traps, light traps, simple crown traps, flight interception traps, cow manure-baited pitfall traps, rodent burrow pitfall traps (Golub et al. 2012, Egorov and Semishin 2016, Ruchin et al. 2020).

L.V. Egorov identified most of Coleoptera taxa while V.B. Semenov and O.I. Semionenkov identified most of Staphylinidae taxa. Other scientists consulted the authors on individual taxa: S.K. Alekseev (Carabidae), A.O. Bieńkowski (Chrysomelidae), M.L. Danilevsky (Cerambycidae), A.A. Gusakov (Scarabaeidae), B.M. Kataev (Carabidae), S.V. Kazantsev (Cantharidae), A.G. Kirejtshuk (Nitidulidae), A.V. Kovalev (Eucnemidae), B.A. Korotyaev (Brentidae, Curculionidae), S.A. Kurbatov (Pselaphinae, Scydmaenidae), A.A. Legalov (Rhynchitidae), K.V. Makarov (Carabidae), M.Yu. Mandelshtam (Scolytinae), A.S. Prosvirov (Elateridae), A.S. Sazhnev (Heteroceridae), A.M. Shapovalov (Cerambycidae), W. Tomaszewska (Anamorphidae), S.E. Tshernyshev (Byrrhidae), M.G. Volkovitsh (Buprestidae) and A.V. Zemoglyadchuk (Mordellidae).

The classification of the family-group taxa used in this checklist predominantly follows Bouchard et al. (2011) and its latest revision (Bouchard and Bousquet 2020). Changes are taken into account from the Catalogue of Palaearctic Coleoptera (Löbl and Smetana 2011, 2013, Löbl I and Löbl D 2015, 2016, 2017), and from the papers of Robertson et al. (2015) on Cucujoidea, and Alonso-Zarazaga et al. (2017) on Curculionoidea. The Scydmaenidae is considered a separate family (Kurbatov and Egorov 2012). The classification and nomenclature of Cerambycidae are given according to Danilevsky (2019). To clarify the nomenclature, the cited studies were used, as well as the Catalogue of Palaearctic Coleoptera (Löbl and Smetana 2007, 2008, 2010) and a number of more recent publications (Kazantsev 2011, Huang and Colonelli 2014, Schimmel et al. 2015, Bieńkowski 2019, Nikitsky 2019, Nilsson and Hájek 2019). The years of description of some species are used as specified by Bousquet (2016). The order used in the checklist is phylogenetic for superfamilies, families, and subfamilies, starting with the accepted most basal-grade taxa, and is alphabetical for supertribes, tribes, and subtribes. Genera and species are listed alphabetically.

The authors of all scientific names are listed along with the date of publication of the taxa. To avoid confusion with authors with same last name, initials are included for some authors.

An asterisk [*] after a species name indicates that the taxon is recorded for the first time for the Mordovia State Nature Reserve and for the Republic of Mordovia, a dagger [†] denotes an adventive species in European Russia. The adventive species were specified according to Orlova-Bienkowskaja (2019). A question mark [?] in front of the species name indicates that confirmation of the record of the species in the reserve is necessary (links to publications are provided). The reference or location for some species is included in brackets {…}.

## Results

### Checklist of the Coleoptera (Insecta) of Mordovia State Nature Reserve (Republic of Mordovia, Russia).

**Order COLEOPTERA Linnaeus, 1758**

**Suborder MYXOPHAGA Crowson, 1955**

**Superfamily SPHAERIUSOIDEA Erichson, 1845**

**Family SPHAERIUSIDAE Erichson, 1845**

*Sphaerius acaroides* Waltl, 1838

**Suborder ADEPHAGA Schellenberg, 1806**

**Family GYRINIDAE Latreille, 1810**

**Subfamily GYRININAE Latreille, 1810**

**Tribe Gyrinini Latreille, 1810**

Subtribe Gyrinina Latreille, 1810

Gyrinus (Gyrinulus) minutus Fabricius, 1798

Gyrinus (Gyrinus) aeratus Stephens, 1835

Gyrinus (Gyrinus) marinus Gyllenhal, 1808

Gyrinus (Gyrinus) natator (Linnaeus, 1758)

Gyrinus (Gyrinus) substriatus Stephens, 1828

**Family CARABIDAE Latreille, 1802**

**Subfamily NEBRIINAE Laporte, 1834**

**Tribe Nebriini Laporte, 1834**

Leistus (Leistus) ferrugineus (Linnaeus, 1758)

Leistus (Leistus) terminatus (Panzer, 1793)

**Tribe Notiophilini Motschulsky, 1850**

*Notiophilus aquaticus* (Linnaeus, 1758)

*Notiophilus biguttatus* (Fabricius, 1779)

*Notiophilus germinyi* Fauvel, 1863

*Notiophilus palustris* (Duftschmid, 1812)

**Subfamily CARABINAE Latreille, 1802**

**Tribe Carabini Latreille, 1802**

Subtribe Calosomatina Bonelli, 1810

Calosoma (Calosoma) inquisitor inquisitor (Linnaeus, 1758)

Calosoma (Calosoma) investigator (Illiger, 1798)

Calosoma (Calosoma) maderae maderae (Fabricius, 1775)

Subtribe Carabina Latreille, 1802

Carabus (Archicarabus) nemoralis nemoralis O.F. Müller, 1764

Carabus (Carabus) arvensis baschkiricus Breuning, 1932

Carabus (Carabus) granulatus granulatus Linnaeus, 1758

Carabus (Carabus) stscheglowi Mannerheim, 1827

Carabus (Hemicarabus) nitens Linnaeus, 1758

Carabus (Limnocarabus) clathratus clathratus Linnaeus, 1760

Carabus (Megodontus) schoenherri schoenherri Fischer von Waldheim, 1820

Carabus (Megodontus) violaceus aurolimbatus Dejean, 1830

Carabus (Pachystus) glabratus glabratus Paykull, 1790

Carabus (Pachystus) hortensis hortensis Linnaeus, 1758

Carabus (Procrustes) coriaceus coriaceus Linnaeus, 1758

Carabus (Tachypus) cancellatus cancellatus Illiger, 1798

Carabus (Tomocarabus) convexus convexus Fabricius, 1775

Carabus (Trachycarabus) estreicheri Fischer von Waldheim, 1820

**Tribe Cychrini Perty, 1830**

Subtribe Cychrina Perty, 1830

Cychrus (Cychrus) caraboides caraboides (Linnaeus, 1758)

**Subfamily CICINDELINAE Latreille, 1802**

**Tribe Cicindelini Latreille, 1802**

Subtribe Cicindelina Latreille, 1802

Cicindela (Cicindela) campestris campestris Linnaeus, 1758

Cicindela (Cicindela) hybrida hybrida Linnaeus, 1758

Cicindela (Cicindela) sylvatica sylvatica Linnaeus, 1758

Cylindera (Cylindera) germanica germanica (Linnaeus, 1758)

**Subfamily LORICERINAE Bonelli, 1810**

**Tribe Loricerini Bonelli, 1810**

Loricera (Loricera) pilicornis (Fabricius, 1775)

**Subfamily ELAPHRINAE Latreille, 1802**

**Tribe Elaphrini Latreille, 1802**

*Blethisa multipunctata multipunctata* (Linnaeus, 1758)

Elaphrus (Elaphrus) riparius (Linnaeus, 1758)

Elaphrus (Neoelaphrus) cupreus Duftschmid, 1812

**Subfamily OMOPHRONINAE Bonelli, 1810**

**Tribe Omophronini Bonelli, 1810**

Omophron (Omophron) limbatum (Fabricius, 1777)

**Subfamily SCARITINAE Bonelli, 1810**

**Tribe Clivinini Rafinesque, 1815**

Subtribe Clivinina Rafinesque, 1815

? Clivina (Clivina) collaris (Herbst, 1784) {Feoktistov 2008}

Clivina (Clivina) fossor fossor (Linnaeus, 1758)

**Tribe Dyschiriini H.J. Kolbe, 1880**

Dyschirius (Dyschiriodes) aeneus aeneus (Dejean, 1825)

Dyschirius (Dyschiriodes) nitidus nitidus (Dejean, 1825)

Dyschirius (Dyschiriodes) politus politus (Dejean, 1825)

Dyschirius (Dyschiriodes) tristis Stephens, 1827

Dyschirius (Dyschirius) thoracicus (P. Rossi, 1790)

Dyschirius (Eudyschirius) globosus (Herbst, 1784)

**Subfamily BROSCINAE Hope, 1838**

**Tribe Broscini Hope, 1838**

Subtribe Broscina Hope, 1838

Broscus (Broscus) cephalotes (Linnaeus, 1758)

*Miscodera arctica* (Paykull, 1798)

**Subfamily TRECHINAE Bonelli, 1810**

**Tribe Bembidiini Stephens, 1827**

Subtribe Bembidiina Stephens, 1827

*Asaphidion flavipes* (Linnaeus, 1760)

Bembidion (Bembidion) quadrimaculatum (Linnaeus, 1760)

Bembidion (Bracteon) litorale (G.-A. Olivier, 1790)

Bembidion (Eupetedromus) dentellum (Thunberg, 1787)

Bembidion (Metallina) lampros (Herbst, 1784)

Bembidion (Metallina) properans (Stephens, 1828)

Bembidion (Notaphus) obliquum Sturm, 1825

Bembidion (Notaphus) varium (G.-A. Olivier, 1795)

Bembidion (Paraprincidium) ruficolle (Panzer, 1796)

Bembidion (Peryphus) bruxellense Wesmael, 1835

Bembidion (Peryphus) bualei polonicum J. Müller, 1930

[indicated by us as *B. andreae* (Fabricius, 1787) (Ruchin et al. 2016)]

Bembidion (Peryphus) tetracolum tetracolum Say, 1823

Bembidion (Philochthus) biguttatum (Fabricius, 1779)

Bembidion (Philochthus) guttula (Fabricius, 1792)

Bembidion (Philochthus) mannerheimii C.R. Sahlberg, 1827

Bembidion (Philochthus) gilvipes Sturm, 1825

Bembidion (Semicampa) schueppelii Dejean, 1831

Bembidion (Trepanedoris) doris (Panzer, 1796)

Bembidion (Trepanes) articulatum (Panzer, 1796)

Bembidion (Trepanes) octomaculatum (Goeze, 1777)

Subtribe Tachyina Motschulsky, 1862

*Porotachys bisulcatus* (Nicolai, 1822)

Tachys (Paratachys) micros (Fischer von Waldheim, 1828)

*Tachyta nana nana* (Gyllenhal, 1810)

**Tribe Trechini Bonelli, 1810**

Subtribe Trechina Bonelli, 1810

*Blemus discus discus* (Fabricius, 1792)

Trechus (Epaphius) secalis (Paykull, 1790)

Trechus (Trechus) quadristriatus (Schrank, 1781)

Trechus (Trechus) rubens (Fabricius, 1792)

**Subfamily PATROBINAE Kirby, 1837**

**Tribe Patrobini Kirby, 1837**

Subtribe Patrobina Kirby, 1837

*Patrobus assimilis* Chaudoir, 1844

*Patrobus atrorufus* (Strøm, 1768)

*Patrobus septentrionis volgensis* Zamotajlov & Isaev, 2006

**Subfamily BRACHININAE Bonelli, 1810**

**Tribe Brachinini Bonelli, 1810**

Subtribe Brachinina Bonelli, 1810

*Brachinus nigricornis* Gebler, 1830

**Subfamily HARPALINAE Bonelli, 1810**

**Tribe Chlaeniini Brullé, 1834**

Subtribe Callistina Laporte, 1834

*Callistus lunatus lunatus* (Fabricius, 1775)

Subtribe Chlaeniina Brullé, 1834

Chlaenius (Chlaeniellus) nigricornis (Fabricius, 1787)

Chlaenius (Chlaeniellus) nitidulus (Schrank, 1781)

Chlaenius (Chlaeniellus) tristis tristis (Schaller, 1783)

**Tribe Harpalini Bonelli, 1810**

Subtribe Anisodactylina Lacordaire, 1854

Anisodactylus (Anisodactylus) binotatus (Fabricius, 1787)

Anisodactylus (Anisodactylus) nemorivagus (Duftschmid, 1812)

Anisodactylus (Paeudanisodactylus) signatus (Panzer, 1796)

Subtribe Harpalina Bonelli, 1810

Harpalus (Harpalus) affinis (Schrank, 1781)

? Harpalus (Harpalus) amplicollis Ménétriés, 1848 {Feoktistov 2008}

Harpalus (Harpalus) anxius (Duftschmid, 1812)

Harpalus (Harpalus) autumnalis (Duftschmid, 1812)

Harpalus (Harpalus) distinguendus distinguendus (Duftschmid, 1812)

Harpalus (Harpalus) flavescens (Piller & Mitterpacher, 1783)

Harpalus (Harpalus) froelichii Sturm, 1818

Harpalus (Harpalus) laevipes Zetterstedt, 1828

Harpalus (Harpalus) latus (Linnaeus, 1758)

Harpalus (Harpalus) luteicornis (Duftschmid, 1812)

Harpalus (Harpalus) modestus Dejean, 1829

Harpalus (Harpalus) picipennis (Duftschmid, 1812)

Harpalus (Harpalus) progrediens Schauberger, 1922

Harpalus (Harpalus) pumilus Sturm, 1818

Harpalus (Harpalus) rubripes (Duftschmid, 1812)

Harpalus (Harpalus) smaragdinus (Duftschmid, 1812)

Harpalus (Harpalus) solitaris Dejean, 1829

Harpalus (Harpalus) tardus (Panzer, 1796)

Harpalus (Harpalus) xanthopus winkleri Schauberger, 1923

Harpalus (Harpalus) zabroides Dejean, 1829

Harpalus (Pseudoophonus) calceatus (Duftschmid, 1812)

Harpalus (Pseudoophonus) griseus (Panzer, 1796)

Harpalus (Pseudoophonus) rufipes (De Geer, 1774)

Harpalus (Semiophonus) signaticornis (Duftschmid, 1812) {ZIN}

Ophonus (Hesperophonus) azureus (Fabricius, 1775)

Ophonus (Metophonus) puncticollis (Paykull, 1798)

Ophonus (Metophonus) rufibarbis (Fabricius, 1792)

Ophonus (Ophonus) stictus Stephens, 1828

Subtribe Stenolophina Kirby, 1837

Acupalpus (Acupalpus) exiguus Dejean, 1829

Acupalpus (Acupalpus) flavicollis (Sturm, 1825)

Acupalpus (Acupalpus) meridianus (Linnaeus, 1760)

Acupalpus (Acupalpus) parvulus (Sturm, 1825)

*Anthracus consputus* (Duftschmid, 1812)

Stenolophus (Stenolophus) mixtus (Herbst, 1784)

Stenolophus (Stenolophus) teutonus (Schrank, 1781)

**Tribe Lebiini Bonelli, 1810**

Subtribe Cymindidina Laporte, 1834

Cymindis (Tarsostinus) macularis Fischer von Waldheim, 1824

Cymindis (Tarulus) vaporariorum (Linnaeus, 1758)

Subtribe Demetriadina Bates, 1886

Demetrias (Demetrias) monostigma Samouelle, 1819

Subtribe Dromiusina Bonelli, 1810

Dromius (Dromius) agilis (Fabricius, 1787)

Dromius (Dromius) fenestratus (Fabricius, 1794)

Dromius (Dromius) quadraticollis A. Morawitz, 1862

Dromius (Dromius) schneideri Crotch, 1871*

*Microlestes maurus maurus* (Sturm, 1827)

*Microlestes minutulus* (Goeze, 1777)

Paradromius (Manodromius) linearis (G.-A. Olivier, 1795)

? *Philorhizus notatus* (Stephens, 1827) {Feoktistov 2008}

*Philorhizus sigma* (P. Rossi, 1790)

*Syntomus foveatus* (Geoffroy, 1785)

Subtribe Lebiina Bonelli, 1810

Lebia (Lamprias) chlorocephala (J.J. Hoffmann, 1803)

Lebia (Lebia) cruxminor cruxminor (Linnaeus, 1758)

Lebia (Lebia) marginata (Geoffroy, 1785)

**Tribe Licinini Bonelli, 1810**

Subtribe Licinina Bonelli, 1810

Badister (Badister) bullatus (Schrank, 1798)

Badister (Badister) lacertosus lacertosus Sturm, 1815

Badister (Badister) meridionalis Puel, 1925

Badister (Badister) unipustulatus Bonelli, 1813

Badister (Baudia) collaris Motschulsky, 1844

Badister (Baudia) dilatatus Chaudoir, 1837

Badister (Baudia) peltatus peltatus (Panzer, 1796)

Badister (Trimorphus) sodalis (Duftschmid, 1812)

Licinus (Licinus) depressus (Paykull, 1790)

**Tribe Odacanthini Laporte, 1834**

Odacantha (Odacantha) melanura (Linnaeus, 1767)

**Tribe Oodini La Ferté-Sénectère, 1851**

*Oodes gracilis* A.Villa & G.B. Villa, 1833

*Oodes helopioides* (Fabricius, 1792)

**Tribe Panagaeini Bonelli, 1810**

Panagaeus (Panagaeus) bipustulatus (Fabricius, 1775)

Panagaeus (Panagaeus) cruxmajor (Linnaeus, 1758)

**Tribe Patynini Bonelli, 1810**

Agonum (Agonum) gracilipes (Duftschmid, 1812)

Agonum (Agonum) marginatum (Linnaeus, 1758)

Agonum (Agonum) muelleri (Herbst, 1784)

Agonum (Europhilus) fuliginosum (Panzer, 1809)

Agonum (Europhilus) gracile Sturm, 1824

Agonum (Europhilus) micans (Nicolai, 1822)

Agonum (Europhilus) piceum (Linnaeus, 1758)

Agonum (Europhilus) thoreyi Dejean, 1828

Agonum (Olisares) dolens (C.R. Sahlberg, 1827)

? Agonum (Olisares) duftschmidi J. Schmidt, 1994 [identification of species of this group is difficult; this may be A. (O.) emarginatum (Gyllenhal, 1827)]

Agonum (Olisares) ericeti (Panzer, 1809)

Agonum (Olisares) impressum (Panzer, 1796)

Agonum (Olisares) hypocrita (Apfelbeck, 1904)

Agonum (Olisares) lugens (Duftschmid, 1812)

Agonum (Olisares) sexpunctatum (Linnaeus, 1758)

Agonum (Olisares) versutum Sturm, 1824

Agonum (Olisares) viduum (Panzer, 1796)

*Anchomenus dorsalis dorsalis* (Pontoppidan, 1763)

*Limodromus assimilis* (Paykull, 1790)

*Limodromus krynickii* (Sperk, 1835)

*Limodromus longiventris* Mannerheim, 1825

*Oxypselaphus obscurus* (Herbst, 1784)

*Platynus livens* (Gyllenhal, 1810)

*Platynus mannerheimii* (Dejean, 1828)

*Sericoda quadripunctata* (De Geer, 1774)

**Tribe Pterostichini Bonelli, 1810**

Poecilus (Ancholeus) crenuliger crenuliger Chaudoir, 1876

Poecilus (Poecilus) cupreus cupreus (Linnaeus, 1758)

Poecilus (Poecilus) lepidus lepidus (Leske, 1785)

Poecilus (Poecilus) punctulatus (Schaller, 1783)

Poecilus (Poecilus) versicolor (Sturm, 1824)

Pterostichus (Adelosia) macer macer (Marsham, 1802)

Pterostichus (Argutor) vernalis (Panzer, 1796)

Pterostichus (Bothriopterus) oblongopunctatus oblongopunctatus (Fabricius, 1787)

Pterostichus (Bothriopterus) quadrifoveolatus Letzner, 1852

Pterostichus (Eosteropus) mannerheimii (Dejean, 1831)

Pterostichus (Melanius) aterrimus aterrimus (Herbst, 1784)

Pterostichus (Morphnosoma) melanarius melanarius (Illiger, 1798)

Pterostichus (Petrophilus) uralensis uralensis (Motschulsky, 1850)

Pterostichus (Phaenoraphis) diligens (Sturm, 1824)

Pterostichus (Phaenoraphis) strenuus (Panzer, 1796)

Pterostichus (Platysma) niger (Schaller, 1783)

Pterostichus (Pseudomaseus) anthracinus anthracinus (Illiger, 1798)

Pterostichus (Pseudomaseus) gracilis gracilis (Dejean, 1828)

Pterostichus (Pseudomaseus) minor minor (Gyllenhal, 1827)

Pterostichus (Pseudomaseus) nigrita nigrita (Paykull, 1790)

Pterostichus (Pseudomaseus) rhaeticus Heer, 1837

Stomis (Stomis) pumicatus pumicatus (Panzer, 1796)

**Tribe Sphodrini Laporte, 1834**

Subtribe Calathina Laporte, 1834

Calathus (Calathus) fuscipes fuscipes (Goeze, 1777)

Calathus (Lindrothius) ambiguus ambiguus (Paykull, 1790)

Calathus (Lindrothius) erratus erratus (C.R. Sahlberg, 1827)

Calathus (Lindrothius) melanocephalus melanocephalus (Linnaeus, 1758)

Calathus (Lindrothius) micropterus (Duftschmid, 1812)

Subtribe Synuchina Lindroth, 1956

Synuchus (Synuchus) vivalis vivalis (Illiger, 1798)

**Tribe Zabrini Bonelli, 1810**

Subtribe Amarina C.C.A. Zimmermann, 1832

Amara (Amara) aenea (De Geer, 1774)

Amara (Amara) communis (Panzer, 1797)

Amara (Amara) convexior Stephens, 1828

Amara (Amara) curta Dejean, 1828

Amara (Amara) eurynota (Panzer, 1796)

Amara (Amara) famelica C.C.A. Zimmermann, 1832

Amara (Amara) familiaris (Duftschmid, 1812)

Amara (Amara) littorea C.G. Thomson, 1857

Amara (Amara) lunicollis Schiødte, 1837

Amara (Amara) montivaga Sturm, 1825

Amara (Amara) ovata (Fabricius, 1792)

Amara (Amara) similata (Gyllenhal, 1810)

Amara (Amara) spreta Dejean, 1831

Amara (Amara) tibialis (Paykull, 1798)

Amara (Amarocelia) erratica (Duftschmid, 1812)

Amara (Bradytus) apricaria (Paykull, 1790)

Amara (Bradytus) consularis (Duftschmid, 1812)

Amara (Bradytus) crenata Dejean, 1828

Amara (Bradytus) fulva (O.F. Müller, 1776)

Amara (Bradytus) majuscula (Chaudoir, 1850)

Amara (Celia) bifrons (Gyllenhal, 1810)

Amara (Celia) brunnea (Gyllenhal, 1810)

Amara (Celia) infima (Duftschmid, 1812)

Amara (Celia) praetermissa (C.R. Sahlberg, 1827)

Amara (Curtonotus) aulica (Panzer, 1796)

? Amara (Curtonotus) convexiuscula (Marsham, 1802) {Feoktistov 2008}

Amara (Curtonotus) gebleri Dejean, 1831

Amara (Paracelia) quenseli silvicola C.C.A. Zimmermann, 1832

Amara (Percosia) equestris equestris (Duftschmid, 1812)

Amara (Xenocelia) ingenua (Duftschmid, 1812)

Amara (Xenocelia) municipalis (Duftschmid, 1812)

Amara (Zezea) plebeja (Gyllenhal, 1810)

**Family HALIPLIDAE Aubé, 1836**

Haliplus (Haliplus) fluviatilis Aubé, 1836

Haliplus (Haliplus) fulvicollis Erichson, 1837

Haliplus (Haliplus) lineolatus Mannerheim, 1844

Haliplus (Haliplus) ruficollis (De Geer, 1774)

**Family NOTERIDAE C.G. Thomson, 1860**

**Subfamily NOTERINAE C.G. Thomson, 1860**

**Tribe Noterini C.G. Thomson, 1860**

*Noterus clavicornis* (De Geer, 1774)

*Noterus crassicornis* (O.F. Müller, 1776)

**Family DYTISCIDAE Leach, 1815**

**Subfamily AGABINAE C.G. Thomson, 1867**

**Tribe Agabini C.G. Thomson, 1867**

Agabus (Acatodes) congener (Thunberg, 1794)

Agabus (Acatodes) fuscipennis (Paykull, 1798)

Agabus (Acatodes) sturmii (Gyllenhal, 1808)

Agabus (Agabus) uliginosus (Linnaeus, 1760)

Agabus (Gaurodytes) affinis (Paykull, 1798)

Agabus (Gaurodytes) biguttatus (G.-A. Olivier, 1795)

Agabus (Gaurodytes) guttatus guttatus (Paykull, 1798)

Agabus (Gaurodytes) melanarius Aubé, 1837

*Ilybius aenescens* C.G. Thomson, 1870

*Ilybius ater* (De Geer, 1774)

*Ilybius erichsoni* (Gemminger & Harold, 1868)

*Ilybius fenestratus* (Fabricius, 1781)

*Ilybius fuliginosus fuliginosus* (Fabricius, 1792)

*Ilybius guttiger* (Gyllenhal, 1808)

*Ilybius neglectus* (Erichson, 1837)

*Ilybius quadriguttatus* (Lacordaire, 1835)

*Ilybius similis* C.G. Thomson, 1856

*Ilybius subaeneus* Erichson, 1837

*Ilybius subtilis* (Erichson, 1837)

*Ilybius wasastjernae* (C.R. Sahlberg, 1824) {ZIN}

*Platambus maculatus* (Linnaeus, 1758)

**Subfamily COLYMBETINAE Erichson, 1837**

**Tribe Colymbetini Erichson, 1837**

*Colymbetes paykulli* Erichson, 1837

*Colymbetes striatus* (Linnaeus, 1758)

Rhantus (Nartus) grapii (Gyllenhal, 1808)

Rhantus (Rhantus) exsoletus (Forster, 1771)

Rhantus (Rhantus) frontalis (Marsham, 1802)

Rhantus (Rhantus) latitans Sharp, 1882

Rhantus (Rhantus) notaticollis (Aubé, 1837)

Rhantus (Rhantus) suturellus (Harris, 1828)

*Liopterus haemorrhoidalis* (Fabricius, 1787)

**Subfamily CYBISTRINAE Sharp, 1880**

**Tribe Cybistrini Sharp, 1880**

Cybister (Cybister) lateralimarginalis lateralimarginalis (De Geer, 1774)

**Subfamily DYTISCINAE Leach, 1815**

**Tribe Aciliini C.G. Thomson, 1867**

Acilius (Acilius) canaliculatus (Nicolai, 1822)

Acilius (Acilius) sulcatus (Linnaeus, 1758)

*Graphoderus bilineatus* (De Geer, 1774)

*Graphoderus cinereus* (Linnaeus, 1758)

*Graphoderus zonatus zonatus* (Hoppe, 1795)

**Tribe Dytiscini Leach, 1815**

*Dytiscus circumcinctus* Ahrens, 1811

*Dytiscus latissimus* Linnaeus, 1758

*Dytiscus marginalis marginalis* Linnaeus, 1758

*Dytiscus thianschanicus* (Gschwendtner, 1923)

**Tribe Hydaticini Sharp, 1880**

Hydaticus (Hydaticus) aruspex H. Clark, 1864

Hydaticus (Hydaticus) continentalis J. Balfour-Browne, 1944

Hydaticus (Hydaticus) seminiger (De Geer, 1774)

Hydaticus (Hydaticus) transversalis transversalis (Pontoppidan, 1763)

**Subfamily HYDROPORINAE Aubé, 1836**

**Tribe Bidessini Sharp, 1880**

*Bidessus grossepunctatus* Vorbringer, 1907

*Bidessus unistriatus* (Goeze, 1777)

*Hydroglyphus geminus* (Fabricius, 1792)

**Tribe Hydroporini Aubé, 1836**

Subtribe Hydroporina Aubé, 1836

*Hydroporus angustatus* Sturm, 1835

*Hydroporus dorsalis* (Fabricius, 1787)

*Hydroporus erythrocephalus* (Linnaeus, 1758)

*Hydroporus fuscipennis* Schaum, 1867

*Hydroporus incognitus* Sharp, 1869

*Hydroporus memnonius* Nicolai, 1822

*Hydroporus neglectus* Schaum, 1845

*Hydroporus palustris* (Linnaeus, 1760)

*Hydroporus planus* (Fabricius, 1782)

*Hydroporus scalesianus* Stephens, 1828

*Hydroporus striola* (Gyllenhal, 1826)

*Hydroporus tristis* (Paykull, 1798)

Subtribe Siettitiina Smrž, 1982

*Graptodytes bilineatus* (Sturm, 1835)

*Graptodytes granularis* (Linnaeus, 1767)

*Graptodytes pictus* (Fabricius, 1787)

*Porhydrus lineatus* (Fabricius, 1775)

**Tribe Hygrotini Portevin, 1929**

Hygrotus (Coelambus) impressopunctatus (Schaller, 1783)

Hygrotus (Hygrotus) decoratus (Gyllenhal, 1810)

Hygrotus (Hygrotus) inaequalis (Fabricius, 1777)

Hygrotus (Hygrotus) versicolor (Schaller, 1783)

**Tribe Hyphydrini Gistel, 1848**

*Hyphydrus ovatus* (Linnaeus, 1760)

**Tribe Laccornini Wolfe & Roughley, 1990**

*Laccornis oblongus* (Stephens, 1835)

**Subfamily LACCOPHILINAE Gistel, 1848**

**Tribe Laccophilini Gistel, 1848**

*Laccophilus hyalinus* (De Geer, 1774)

*Laccophilus minutus* (Linnaeus, 1758)

**Suborder POLYPHAGA Emery, 1886**

**Superfamily HYDROPHILOIDEA Latreille, 1802**

**Family HELOPHORIDAE Leach, 1815**

Helophorus (Kyphohelophorus) tuberculatus Gyllenhal, 1808

**Family GEORISSIDAE Laporte, 1840**

Georissus (Georissus) crenulatus (P. Rossi, 1794)

**Family HYDROCHIDAE C.G. Thomson, 1859**

*Hydrochus brevis* (Herbst, 1793)

*Hydrochus crenatus* (Fabricius, 1792)

*Hydrochus elongatus* (Schaller, 1783)

*Hydrochus kirgisicus* Motschulsky, 1860

**Family HYDROPHILIDAE Latreille, 1802**

**Subfamily HYDROPHILINAE Latreille, 1802**

**Tribe Berosini Mulsant, 1844**

Berosus (Berosus) luridus (Linnaeus, 1760)

Berosus (Berosus) signaticollis (Charpentier, 1825)

**Tribe Laccobiini Houlbert, 1922**

Laccobius (Laccobius) albipes Kuwert, 1890

Laccobius (Laccobius) minutus (Linnaeus, 1758)

**Tribe Hydrobiusini Mulsant, 1844**

*Hydrobius fuscipes* (Linnaeus, 1758)

**Tribe Hydrophilini Latreille, 1802**

*Hydrochara caraboides* (Linnaeus, 1758)

*Hydrophilus aterrimus* Eschscholtz, 1822

**Subfamily CHAETARTHRIINAE Bedel, 1881**

**Tribe Anacaenini M. Hansen, 1991**

*Anacaena lutescens* (Stephens, 1829)

**Tribe Chaetarthriini Bedel, 1881**

*Chaetarthria seminulum* (Herbst, 1797)

**Subfamily ENOCHRINAE Short & Fikáček, 2013**

*Cymbiodyta marginella* (Fabricius, 1792)

Enochrus (Lumetus) bicolor (Fabricius, 1792)

Enochrus (Lumetus) fuscipennis (C.G. Thomson, 1884)

Enochrus (Lumetus) quadripunctatus (Herbst, 1797)

Enochrus (Methydrus) affinis (Thunberg, 1794)

Enochrus (Methydrus) coarctatus (Gredler, 1863)

**Subfamily ACIDOCERINAE Zaitzev, 1908**

Helochares (Helochares) obscurus (O.F. Müller, 1776)

**Subfamily SPHAERIDIINAE Latreille, 1802**

**Tribe Coelostomatini L. Heyden, 1891**

Coelostoma (Coelostoma) orbiculare (Fabricius, 1775)

**Tribe Megasternini Mulsant, 1844**

Cercyon (Cercyon) bifenestratus Küster, 1851

Cercyon (Cercyon) convexiusculus Stephens, 1829

Cercyon (Cercyon) granarius Erichson, 1837

Cercyon (Cercyon) haemorrhoidalis (Fabricius, 1775)

Cercyon (Cercyon) impressus (Sturm, 1807)

Cercyon (Cercyon) lateralis (Marsham, 1802)

Cercyon (Cercyon) marinus C.G. Thomson, 1853

Cercyon (Cercyon) melanocephalus (Linnaeus, 1758)

Cercyon (Cercyon) pygmaeus (Illiger, 1801)

Cercyon (Cercyon) quisquilius (Linnaeus, 1760)

Cercyon (Cercyon) sternalis Sharp, 1918

Cercyon (Cercyon) tristis (Illiger, 1801)

Cercyon (Cercyon) unipunctatus (Linnaeus, 1758)

Cercyon (Conocercyon) ustulatus (Preyssler, 1790)

Cercyon (Paracercyon) analis (Paykull, 1798)

Cercyon (Paracycreon) laminatus Sharp, 1873†

*Cryptopleurum crenatum* (Kugelann, 1794)

*Cryptopleurum minutum* (Fabricius, 1775)

**Tribe Sphaeridiini Latreille, 1802**

*Sphaeridium bipustulatum* Fabricius, 1781

*Sphaeridium lunatum* Fabricius, 1792

*Sphaeridium scarabaeoides* (Linnaeus, 1758)

**Family SPHAERITIDAE Shuckard, 1839**

*Sphaerites glabratus* (Fabricius, 1792)

**Family HISTERIDAE Gyllenhal, 1808**

**Subfamily ABRAEINAE W.S. MacLeay, 1819**

**Tribe Acritini Wenzel, 1944**

Acritus (Acritus) minutus (Herbst, 1791)

Acritus (Pycnacritus) homoeopathicus Wollaston, 1857

**Tribe Plegaderini Portevin, 1929**

Plegaderus (Plegaderus) caesus (Herbst, 1791)

Plegaderus (Plegaderus) saucius Erichson, 1834

Plegaderus (Plegaderus) vulneratus (Panzer, 1797)

**Subfamily DENDROPHILINAE Reitter, 1909**

**Tribe Dendrophilini Reitter, 1909**

Dendrophilus (Dendrophilus) punctatus punctatus (Herbst, 1791)

Dendrophilus (Dendrophilus) pygmaeus (Linnaeus, 1758)*

**Tribe Paromalini Reitter, 1909**

Paromalus (Paromalus) flavicornis (Herbst, 1791)

Paromalus (Paromalus) parallelepipedus (Herbst, 1791)

*Platylomalus complanatus* (Panzer, 1797)

**Subfamily HISTERINAE Gyllenhal, 1808**

**Tribe Histerini Gyllenhal, 1808**

*Atholus duodecimstriatus duodecimstriatus* (Schrank, 1781)

*Hister bissexstriatus* Fabricius, 1801

*Hister funestus* Erichson, 1834

*Hister unicolor unicolor* Linnaeus, 1758

Margarinotus (Eucalohister) bipustulatus (Schrank, 1781)

Margarinotus (Paralister) neglectus (Germar, 1813)

Margarinotus (Paralister) purpurascens (Herbst, 1791)

Margarinotus (Paralister) ventralis (Marseul, 1854)

Margarinotus (Ptomister) brunneus (Fabricius, 1775)

Margarinotus (Ptomister) merdarius (J.J. Hoffmann, 1803)

Margarinotus (Ptomister) striola striola (C.R. Sahlberg, 1819)

Margarinotus (Ptomister) terricola (Germar, 1823)

**Tribe Hololeptini Hope, 1840**

Hololepta (Hololepta) plana (Sulzer, 1776)

**Tribe Platysomatini Bickhardt, 1914**

*Eurosomides minor* (P. Rossi, 1790)

Platysoma (Cylister) angustatum (J.J. Hoffmann, 1803)

Platysoma (Cylister) elongatum elongatum (Thunberg, 1787)

Platysoma (Cylister) lineare Erichson, 1834

Platysoma (Platysoma) deplanatum (Gyllenhal, 1808)

**Subfamily SAPRININAE C.É. Blanchard, 1845**

*Chalcionellus decemstriatus decemstriatus* (P. Rossi, 1792)

*Gnathoncus buyssoni* Auzat, 1917*

*Gnathoncus nannetensis* (Marseul, 1862)

Hypocaccus (Hypocaccus) rugifrons (Paykull, 1798)

*Myrmetes paykulli* Kanaar, 1979

Saprinus (Saprinus) aeneus (Fabricius, 1775)

Saprinus (Saprinus) caerulescens caerulescens (J.J. Hoffmann, 1803)

Saprinus (Saprinus) planiusculus Motschulsky, 1849

Saprinus (Saprinus) rugifer (Paykull, 1809)*

Saprinus (Saprinus) semistriatus (L.G. Scriba, 1790)

**Superfamily STAPHYLINOIDEA Latreille, 1802**

**Family HYDRAENIDAE Mulsant, 1844**

**Subfamily HYDRAENINAE Mulsant, 1844**

**Tribe Limnebiini Mulsant, 1844**

*Limnebius truncatellus* (Thunberg, 1794)

**Family LEIODIDAE Fleming, 1821**

**Subfamily CHOLEVINAE Kirby, 1837**

**Tribe Cholevini Kirby, 1837**

Subtribe Catopina Chaudoir, 1845

*Apocatops nigrita* (Erichson, 1837)

*Fissocatops westi* (Krogerus, 1931)

*Sciodrepoides fumatus* (Spence, 1813)

*Sciodrepoides watsoni watsoni* (Spence, 1813)

Subtribe Cholevina Kirby, 1837

Choleva (Choleva) oblonga oblonga Latreille, 1806*

**Subfamily COLONINAE Horn, 1880**

*Colon serripes* (C.R. Sahlberg, 1822)

**Subfamily LEIODINAE Fleming, 1821**

**Tribe Anisotomini Horaninow, 1834**

Agathidium (Cyphoceble) discoideum Erichson, 1845

Agathidium (Neoceble) nigripenne (Fabricius, 1792)

Agathidium (Neoceble) rotundatum rotundatum (Gyllenhal, 1827)

*Amphicyllis globus* (Fabricius, 1792)

*Anisotoma axillaris* Gyllenhal, 1810

*Anisotoma castanea castanea* (Herbst, 1791)

*Anisotoma glabra* (Fabricius, 1787)

*Anisotoma humeralis* (Herbst, 1791)

*Anisotoma orbicularis* (Herbst, 1791)

*Liodopria serricornis* (Gyllenhal, 1813)

**Tribe Leiodini Fleming, 1821**

*Cyrtusa subtestacea* (Gyllenhal, 1813)

**Tribe Pseudoliodini Portevin, 1926**

Colenis (Colenis) immunda (Sturm, 1807)

**Family SILPHIDAE Latreille, 1806**

**Subfamily SILPHINAE Latreille, 1806**

*Dendroxena quadrimaculata* (Scopoli, 1771)

*Necrodes littoralis* (Linnaeus, 1758)

*Oiceoptoma thoracicum* (Linnaeus, 1758)

*Phosphuga atrata atrata* (Linnaeus, 1758)

*Silpha carinata* Herbst, 1783

*Silpha obscura obscura* Linnaeus, 1758

*Silpha tristis* Illiger, 1798

*Thanatophilus dispar* (Herbst, 1793)

*Thanatophilus rugosus* (Linnaeus, 1758)

*Thanatophilus sinuatus* (Fabricius, 1775)

**Subfamily NICROPHORINAE Kirby, 1837**

*icrophorus humator* (Gleditsch, 1767)

*Nicrophorus interruptus* Stephens, 1830

*Nicrophorus investigator* Zetterstedt, 1824

*Nicrophorus sepultor* Charpentier, 1825

*Nicrophorus vespillo* (Linnaeus, 1758)

*Nicrophorus vespilloides* Herbst, 1783

**Family STAPHYLINIDAE Latreille, 1802**

**Subfamily OMALIINAE W.S. MacLeay, 1825**

**Tribe Anthophagini C.G. Thomson, 1859**

*Acidota crenata crenata* (Fabricius, 1792)

*Acidota cruentata* Mannerheim, 1830

Anthobium (Anthobium) atrocephalum (Gyllenhal, 1827)

Anthophagus (Dimorphoschelus) angusticollis angusticollis (Mannerheim, 1830)

Anthophagus (Phaganthus) caraboides caraboides (Linnaeus, 1758)

*Arpedium brachypterum* (Gravenhorst, 1802)

*Arpedium quadrum* (Gravenhorst, 1806)

Deliphrum (Deliphrum) tectum (Paykull, 1789)

**Tribe Eusphalerini Hatch, 1957**

*Eusphalerum luteum luteum* (Marsham, 1802)

*Eusphalerum minutum* (Fabricius, 1792)

**Tribe Omaliini W.S. MacLeay, 1825**

*Acrulia inflata* (Gyllenhal, 1813)

*Omalium caesum* Gravenhorst, 1806

*Omalium rivulare* (Paykull, 1789)

Phloeonomus (Phloeonomus) pusillus (Gravenhorst, 1806)

*Phloeostiba lapponica* (Zetterstedt, 1838)

*Phloeostiba plana* (Paykull, 1792)

*Phyllodrepa melanocephala melanocephala* (Fabricius, 1787)

*Phyllodrepa nigra* (Gravenhorst, 1806)

**Subfamily PROTEININAE Erichson, 1839**

**Tribe Proteinini Erichson, 1839**

*Megarthrus denticollis* (Beck, 1817)

*Megarthrus depressus* (Paykull, 1789)

*Megarthrus hemipterus* (Illiger, 1794)

*Proteinus atomarius* Erichson, 1840

*Proteinus brachypterus* (Fabricius, 1792)

*Proteinus laevigatus* Hochhuth, 1872

**Subfamily MICROPEPLINAE Leach, 1815**

Arrhenopeplus (Arrhenopeplus) tesserula (Curtis, 1828)

**Subfamily PSELAPHINAE Latreille, 1802**

**Supertribe Euplectitae Streubel, 1839**

**Tribe Euplectini Streubel, 1839**

*Euplectus karstenii* (Reichenbach, 1816)

*Euplectus kirbii kirbii* Denny, 1825

*Euplectus punctatus* Mulsant & Rey, 1861

**Tribe Trichonychini Reitter, 1882**

Subtribe Bibloporina O. Park, 1951

Bibloporus (Bibloporus) minutus Raffray, 1914

Subtribe Panaphantina Jeannel, 1950

Bibloplectus (Bibloplectus) ambiguus (Reichenbach, 1816)

Subtribe Trichonychina Reitter, 1882

*Trichonyx sulcicollis* (Reichenbach, 1816)*

Subtribe Trimiina Brendel & Wickham, 1890

*Trimium brevicorne* (Reichenbach, 1816)

**Tribe Brachyglutini Raffray, 1904**

Subtribe Brachyglutina Raffray, 1904

Brachygluta (Brachygluta) fossulata (Reichenbach, 1816)

*Brachygluta haematica* (Reichenbach, 1816)

*Fagniezia impressa* (Panzer, 1803)

*Rybaxis longicornis* (Leach, 1817)

**Tribe Bythinini Raffray, 1890**

Subtribe Bythinini Raffray, 1890

*Bryaxis bulbifer* (Reichenbach, 1816)

**Tribe Tychini Raffray, 1904**

*Tychus niger* (Paykull, 1800)

**Supertribe Pselaphitae Latreille, 1802**

**Tribe Pselaphini Latreille, 1802**

*Pselaphaulax dresdensis* (Herbst, 1791)

**Tribe Tyrini Reitter, 1882**

Subtribe Tyrina Reitter, 1882

*Tyrus mucronatus mucronatus* (Panzer, 1803)

**Subfamily PHLOEOCHARINAE Erichson, 1839**

Phloeocharis (Phloeocharis) subtilissima Mannerheim, 1830

**Subfamily TACHYPORINAE W.S. MacLeay, 1825**

**Tribe Mycetoporini C.G. Thomson, 1859**

Bolitobius (Bolitobius) castaneus boreomontanicus Schülke, 2010

*Carphacis striatus* (G.-A. Olivier, 1795)

*Ischnosoma longicorne* (Mäklin, 1847)

*Ischnosoma splendidum* (Gravenhorst, 1806)

*Lordithon exoletus* (Erichson, 1839)

*Lordithon lunulatus* (Linnaeus, 1760)

*Lordithon pulchellus* (Mannerheim, 1830)

*Lordithon speciosus* (Erichson, 1839)

*Lordithon thoracicus thoracicus* (Fabricius, 1777)

*Lordithon trimaculatus* (Fabricius, 1792)

*Mycetoporus bimaculatus* Lacordaire, 1835*

*Mycetoporus lepidus* (Gravenhorst, 1806)

*Mycetoporus maerkelii* Kraatz, 1857

*Mycetoporus monticola* Fowler, 1888

*Mycetoporus punctus* (Gravenhorst, 1806)

*Parabolitobius formosus* (Gravenhorst, 1806)

**Tribe Tachyporini W.S. MacLeay, 1825**

*Lamprinodes saginatus* (Gravenhorst, 1806)

*Sepedophilus binotatus* (Gravenhorst, 1802)

*Sepedophilus bipunctatus* (Gravenhorst, 1802)

*Sepedophilus bipustulatus* (Gravenhorst, 1802)

*Sepedophilus constans* (Fowler, 1888)

*Sepedophilus immaculatus* (Stephens, 1832)

*Sepedophilus littoreus* (Linnaeus, 1758)

*Sepedophilus marshami* (Stephens, 1832)

*Sepedophilus pedicularius* (Gravenhorst, 1802)

*Sepedophilus testaceus* (Fabricius, 1792)

Tachinus (Tachinus) bipustulatus (Fabricius, 1792)

Tachinus (Tachinus) laticollis Gravenhorst, 1802

Tachinus (Tachinus) marginellus marginellus (Fabricius, 1781)*

Tachinus (Tachinus) proximus Kraatz, 1855

Tachinus (Tachinus) rufipes (Linnaeus, 1758)

Tachinus (Tachinus) subterraneus (Linnaeus, 1758)

Tachyporus (Palporus) nitidulus (Fabricius, 1781)

Tachyporus (Tachyporus) abdominalis (Fabricius, 1781)

Tachyporus (Tachyporus) chrysomelinus (Linnaeus, 1758)

Tachyporus (Tachyporus) dispar (Paykull, 1789)

Tachyporus (Tachyporus) formosus A. [H]. Matthews, 1838

Tachyporus (Tachyporus) hypnorum (Fabricius, 1775)

Tachyporus (Tachyporus) obtusus (Linnaeus, 1767)

Tachyporus (Tachyporus) pallidus Sharp, 1871

Tachyporus (Tachyporus) pulchellus Mannerheim, 1843

Tachyporus (Tachyporus) quadriscopulatus quadriscopulatus Pandellé, 1869

Tachyporus (Tachyporus) scitulus Erichson, 1839

Tachyporus (Tachyporus) solutus Erichson, 1839

Tachyporus (Tachyporus) transversalis Gravenhorst, 1806

**Subfamily HABROCERINAE Mulsant & Rey, 1876**

*Habrocerus capillaricornis* (Gravenhorst, 1806)

**Subfamily ALEOCHARINAE Fleming, 1821**

**Tribe Aleocharini Fleming, 1821**

Subtribe Aleocharina Fleming, 1821

Aleochara (Aleochara) curtula (Goeze, 1777)

Aleochara (Ceranota) erythroptera Gravenhorst, 1806

Aleochara (Coprochara) bipustulata (Linnaeus, 1760)

Aleochara (Xenochara) brevipennis Gravenhorst, 1806

Aleochara (Xenochara) falcata Assing, 2009*

Aleochara (Xenochara) fumata Gravenhorst, 1802

Aleochara (Xenochara) grandeguttata Assing, 2009

Aleochara (Xenochara) haematoptera Kraatz, 1858

Aleochara (Xenochara) stichai Likovský, 1965

**Tribe Athetini Casey, 1910**

Subtribe Athetina Casey, 1910

Acrotona (Acrotona) aterrima (Gravenhorst, 1802)

Acrotona (Acrotona) convergens (A. Strand, 1958)

Acrotona (Acrotona) exigua (Erichson, 1837)

Acrotona (Acrotona) muscorum (Brisout de Barneville, 1860)

Acrotona (Acrotona) obfuscata (Gravenhorst, 1802)

Acrotona (Acrotona) parvula (Mannerheim, 1830)

Acrotona (Acrotona) pseudotenera (Cameron, 1933)†

Acrotona (Acrotona) pygmaea (Gravenhorst, 1802)

Acrotona (Acrotona) sylvicola (Kraatz, 1856)

*Alianta incana* (Erichson, 1837)

*Amischa analis* (Gravenhorst, 1802)

*Amischa bifoveolata* (Mannerheim, 1830)

*Amischa decipiens* (Sharp, 1869)

Atheta (Alaobia) gagatina (Baudi di Selve, 1848)

Atheta (Alaobia) pallidicornis (C.G. Thomson, 1856)

Atheta (Alaobia) scapularis (C.R. Sahlberg, 1831)

Atheta (Alaobia) sodalis (Erichson, 1837)

Atheta (Atheta) basicornis (Mulsant & Rey, 1852)

Atheta (Atheta) crassicornis (Fabricius, 1792)

Atheta (Atheta) ebenina (Mulsant & Rey, 1873)

Atheta (Atheta) euryptera (Stephens, 1832)

Atheta (Atheta) harwoodi B.S. Williams, 1930

Atheta (Atheta) paracrassicornis Brundin, 1954

Atheta (Atheta) pilicornis (C.G. Thomson, 1852)

Atheta (Atheta) vaga (Heer, 1839)

Atheta (Badura) cauta (Erichson, 1837)*

Atheta (Bessobia) occulta (Erichson, 1837)

Atheta (Ceritaxa) subterranea (Mulsant & Rey, 1853)

Atheta (Chaetida) longicornis (Gravenhorst, 1802)

Atheta (Datomicra) canescens (Sharp, 1869)

Atheta (Datomicra) dadopora C.G. Thomson, 1867

Atheta (Datomicra) nigra (Kraatz, 1856)

Atheta (Dimetrota) intermedia (Thomson, 1852)

Atheta (Microdota) atomaria (Kraatz, 1856)*

Atheta (Microdota) minuscula (Brisout de Barneville, 1860)

Atheta (Mocyta) clientula (Erichson, 1839)

Atheta (Mocyta) fungi fungi (Gravenhorst, 1806)

Atheta (Mocyta) fussi Bernhauer, 1908

Atheta (Mocyta) orbata (Erichson, 1837)

Atheta (Mycetota) laticollis (Stephens, 1832)

Atheta (Parameotica) laticeps (C.G. Thomson, 1856)

Atheta (Philhygra) britteni Joy, 1913

Atheta (Philhygra) debilis (Erichson, 1837)

Atheta (Philhygra) deformis (Kraatz, 1856)

Atheta (Philhygra) elongatula (Gravenhorst, 1802)

Atheta (Philhygra) gyllenhalii (C.G. Thomson, 1856)

Atheta (Philhygra) hygrobia (C.G. Thomson, 1856)

Atheta (Philhygra) luridipennis (Mannerheim, 1830)

Atheta (Philhygra) malleus Joy, 1913

Atheta (Philhygra) palustris (Kiesenwetter, 1844)

Atheta (Philhygra) pseudoelongatula Bernhauer, 1907

Atheta (Philhygra) sequanica (Brisout de Barneville, 1860)

Atheta (Philhygra) tmolosensis Bernhauer, 1940

Atheta (Tetropla) liturata (Stephens, 1832)

Atheta (Tetropla) nigritula (Gravenhorst, 1802)

Atheta (Xenota) lativentris J.R. Sahlberg, 1876

*Dadobia immersa* (Erichson, 1837)

*Dinaraea aequata* (Erichson, 1837)

*Dochmonota clancula* (Erichson, 1837)

*Eurodotina inquinula* (Gravenhorst, 1802)

*Liogluta microptera* C.G. Thomson, 1867

*Lyprocorrhe anceps* (Erichson, 1837)

*Nehemitropia lividipennis* (Mannerheim, 1830)

Notothecta (Notothecta) flavipes (Gravenhorst, 1806)

*Pachnida nigella* (Erichson, 1837)

Plataraea (Plataraea) dubiosa (G. Benick, 1935)

*Schistoglossa aubei* (Brisout de Barneville, 1860)

*Schistoglossa gemina* (Erichson, 1837)

*Schistoglossa viduata* (Erichson, 1837)

**Tribe Autaliini C.G. Thomson, 1859**

*Autalia longicornis* Scheerpeltz, 1947

**Tribe Deinopsini Sharp, 1883**

*Deinopsis erosa* (Stephens, 1832)

**Tribe Falagriini Mulsant & Rey, 1873**

*Cordalia obscura* (Gravenhorst, 1802)

*Falagria caesa* Erichson, 1837

**Tribe Geostibini Seevers, 1978**

*Alevonota egregia* (Rye, 1876)

*Alevonota gracilenta* (Erichson, 1839)*

*Alevonota rufotestacea* (Kraatz, 1856)

Geostiba (Geostiba) circellaris (Gravenhorst, 1806)

**Tribe Homalotini Heer, 1839**

Subtribe Bolitocharina C.G. Thomson, 1859

*Bolitochara obliqua* Erichson, 1837

*Bolitochara pulchra* (Gravenhorst, 1806)

*Bolitochara tecta* Assing, 2014

Euryusa (Euryusa) castanoptera Kraatz, 1856

Leptusa (Leptusa) pulchella (Mannerheim, 1830)

*Phymatura brevicollis* (Kraatz, 1856)

*Tachyusida gracilis* (Erichson, 1837)

Subtribe Gyrophaenina Kraatz, 1856

Encephalus (Encephalus) complicans Stephens, 1832

Gyrophaena (Agaricophaena) boleti (Linnaeus, 1758)

Gyrophaena (Gyrophaena) bihamata C.G. Thomson, 1867

Gyrophaena (Gyrophaena) fasciata (Marsham, 1802)

Gyrophaena (Gyrophaena) gentilis Erichson, 1839

Gyrophaena (Gyrophaena) joyi Wendler, 1924

Gyrophaena (Gyrophaena) joyioides Wüsthoff, 1937

Gyrophaena (Gyrophaena) lucidula Erichson, 1837

Gyrophaena (Gyrophaena) manca Erichson, 1839

Gyrophaena (Gyrophaena) nitidula (Gyllenhal, 1810)

Gyrophaena (Gyrophaena) orientalis A. Strand, 1938

Gyrophaena (Gyrophaena) poweri Crotch, 1867

Gyrophaena (Leptarthrophaena) affinis Mannerheim, 1830

Gyrophaena (Phaenogyra) strictula Erichson, 1839

Subtribe Homalotina Heer, 1839

*Anomognathus cuspidatus* (Erichson, 1839)

*Cyphea curtula* (Erichson, 1837)

*Homalota plana* (Gyllenhal, 1810)

**Tribe Hygronomini C.G. Thomson, 1859**

Subtribe Hygronomina C.G. Thomson, 1859

*Hygronoma dimidiata* (Gravenhorst, 1806)

**Tribe Hypocyphtini Laporte, 1835**

*Cypha discoidea* (Erichson, 1839)

*Cypha seminulum* (Erichson, 1839)

*Cypha tarsalis* (Luze, 1902)

*Holobus apicatus* (Erichson, 1837)

*Holobus flavicornis* (Lacordaire, 1835)

*Oligota granaria* Erichson, 1837

*Oligota inflata* (Mannerheim, 1830)

*Oligota parva* Kraatz, 1862

*Oligota pusillima* (Gravenhorst, 1806)

**Tribe Lomechusini Fleming, 1821**

Subtribe Lomechusina Fleming, 1821

*Lomechusa pubicollis* Brisout de Barneville, 1860

Subtribe Myrmedoniina C.G. Thomson, 1867

Drusilla (Drusilla) canaliculata (Fabricius, 1787)

*Pella cognata* (Märkel, 1842)

*Pella funesta* (Gravenhorst, 1806)

*Pella laticollis* (Märkel, 1844)

*Pella lugens* (Gravenhorst, 1802)

Zyras (Zyras) collaris (Paykull, 1800)

**Tribe Myllaenini Ganglbauer, 1895**

*Myllaena dubia* (Gravenhorst, 1806)

*Myllaena intermedia* Erichson, 1837

*Myllaena minuta* (Gravenhorst, 1806)

**Tribe Oxypodini C.G. Thomson, 1859**

Subtribe Dinardina Mulsant & Rey, 1873

*Dinarda hagensii* Wasmann, 1889*

*Thiasophila lohsei* Zerche, 1987

Subtribe Meoticina Seevers, 1978

*Meotica exilis* (Gravenhorst, 1806)

*Meotica filiformis* (Motschulsky, 1860)

Subtribe Microglottina Fenyes, 1918

*Crataraea suturalis* (Mannerheim, 1830)

*Haploglossa villosula* (Stephens, 1832)

Subtribe Oxypodina C.G. Thomson, 1859

*Calodera aethiops* (Gravenhorst, 1802)

*Calodera riparia* Erichson, 1837

*Calodera uliginosa* Erichson, 1837

*Dexiogyia corticina* (Erichson, 1837)

*Ilyobates nigricollis* (Paykull, 1800)

Ocalea (Ocalea) badia Erichson, 1837

*Ocyusa maura* (Erichson, 1837)

Oxypoda (Baeoglena) praecox Erichson, 1839

Oxypoda (Bessopora) haemorrhoa (Mannerheim, 1830)

Oxypoda (Disochara) elongatula Aubé, 1850

Oxypoda (Disochara) procerula Mannerheim, 1830

Oxypoda (Mycetodrepa) alternans (Gravenhorst, 1802)

Oxypoda (Oxypoda) acuminata (Stephens, 1832)

Oxypoda (Oxypoda) longipes Mulsant & Rey, 1861

Oxypoda (Oxypoda) opaca (Gravenhorst, 1802)

Oxypoda (Podoxya) brevicornis (Stephens, 1832)

Oxypoda (Podoxya) hansseni A. Strand, 1946

Oxypoda (Podoxya) skalitzkyi Bernhauer, 1902*

Oxypoda (Thliboptera) togata Erichson, 1837

Subtribe Phloeoporina C.G. Thomson, 1859

*Phloeopora corticalis corticalis* (Gravenhorst, 1802)

*Phloeopora nitidiventris* Fauvel, 1900

*Phloeopora testacea* (Mannerheim, 1830)

**Tribe Placusini Mulsant & Rey, 1871**

Placusa (Placusa) atrata (Mannerheim, 1830)

Placusa (Placusa) complanata Erichson, 1839

Placusa (Placusa) depressa Mäklin, 1845

Placusa (Placusa) tachyporoides (Waltl, 1838)

**Tribe Tachyusini C.G. Thomson, 1859**

*Brachyusa concolor* (Erichson, 1839)

*Dasygnypeta velata* (Erichson, 1837)

*Dilacra vilis* (Erichson, 1837)

*Ischnopoda leucopus* (Marsham, 1802)

*Ischnopoda umbratica* (Erichson, 1837)

*Tachyusa coarctata* Erichson, 1837

*Tachyusa constricta* Erichson, 1837

*Tachyusa objecta* Mulsant & Rey, 1870

*Tachyusa atra* (Gravenhorst, 1806)

**Subfamily SCAPHIDIINAE Latreille, 1806**

**Tribe Scaphidiini Latreille, 1806**

*Scaphidium quadrimaculatum* G.-A. Olivier, 1790

**Tribe Scaphisomatini Casey, 1893**

*Scaphisoma agaricinum* (Linnaeus, 1758)

*Scaphisoma assimile assimile* Erichson, 1845*

*Scaphisoma balcanicum* Tamanini, 1954

*Scaphisoma boreale* Lundblad, 1952

*Scaphisoma limbatum* Erichson, 1845

*Scaphisoma subalpinum subalpinum* Reitter, 1880

**Subfamily OXYTELINAE Fleming, 1821**

**Tribe Blediini Ádám, 2001**

Bledius (Astycops) subterraneus Erichson, 1839

Bledius (Astycops) talpa (Gyllenhal, 1810)

Bledius (Bargus) opacus (Block, 1799)

Bledius (Bargus) pallipes (Gravenhorst, 1806)

Bledius (Bledius) tricornis (Herbst, 1784)

Bledius (Dicarenus) fergussoni fergussoni Joy, 1912

Bledius (Hesperophilus) dissimilis Erichson, 1840

Bledius (Hesperophilus) gallicus (Gravenhorst, 1806)

**Tribe Coprophilini Heer, 1839**

Coprophilus (Coprophilus) striatulus (Fabricius, 1792)

**Tribe Oxytelini Fleming, 1821**

*Anotylus hamatus* (Fairmaire & Laboulbène, 1856)

*Anotylus insecatus* (Gravenhorst, 1806)

*Anotylus nitidulus* (Gravenhorst, 1802)

*Anotylus pumilus* (Erichson, 1839)

*Anotylus rugosus* (Fabricius, 1775)

*Anotylus tetracarinatus* (Block, 1799)

Oxytelus (Epomotylus) sculptus Gravenhorst, 1806

Oxytelus (Oxytelus) fulvipes Erichson, 1839

Oxytelus (Oxytelus) migrator Fauvel, 1904†

Oxytelus (Oxytelus) piceus (Linnaeus, 1767)

Oxytelus (Tanycraerus) laqueatus (Marsham, 1802)*

Platystethus (Craetopycrus) cornutus cornutus (Gravenhorst, 1802)

Platystethus (Craetopycrus) nitens (C.R. Sahlberg, 1832)

**Tribe Planeustomini Jacquelin du Val, 1857**

*Manda mandibularis* (Gyllenhal, 1827)

**Tribe Syntomiini Böving & Craighead, 1931**

*Syntomium aeneum* (P. Müller, 1821)

**Tribe Thinobiini J.R. Sahlberg, 1876**

Carpelimus (Carpelimus) fuliginosus (Gravenhorst, 1802)

Carpelimus (Carpelimus) lindrothi lindrothi (Palm, 1943)

Carpelimus (Carpelimus) obesus (Kiesenwetter, 1844)

Carpelimus (Carpelimus) pusillus (Gravenhorst, 1802)

Carpelimus (Paratrogophloeus) bilineatus Stephens, 1834

Carpelimus (Paratrogophloeus) rivularis (Motschulsky, 1860)

Carpelimus (Troginus) exiguus (Erichson, 1839)

Carpelimus (Trogophloeus) corticinus (Gravenhorst, 1806)

Carpelimus (Trogophloeus) elongatulus elongatulus (Erichson, 1839)

Carpelimus (Trogophloeus) gracilis (Mannerheim, 1830)

Carpelimus (Trogophloeus) manchuricus subtilicornis (Roubal, 1946)

Carpelimus (Trogophloeus) modestus Casey, 1889

Thinobius (Thinobius) flagellatus Lohse, 1984

**Subfamily OXYPORINAE** Fleming, 1821

*Oxyporus mannerheimii* Gyllenhal, 1827

*Oxyporus maxillosus* Fabricius, 1792

*Oxyporus rufus* (Linnaeus, 1758)*

**Subfamily STENINAE W.S. MacLeay, 1825**

*Stenus argus* Gravenhorst, 1806 *

*Stenus ater* Mannerheim, 1830

*Stenus bifoveolatus* Gyllenhal, 1827

*Stenus bimaculatus* Gyllenhal, 1810

*Stenus boops boops* Ljungh, 1810

*Stenus carbonarius* Gyllenhal, 1827

*Stenus cicindeloides* (Schaller, 1783)

*Stenus clavicornis* (Scopoli, 1763)

*Stenus comma comma* LeConte, 1863

*Stenus excubitor* Erichson, 1839

*Stenus flavipes flavipes* Stephens, 1833*

*Stenus formicetorum* Mannerheim, 1843

*Stenus fossulatus* Erichson, 1840*

*Stenus gallicus* Fauvel, 1873

*Stenus humilis* Erichson, 1839

*Stenus incrassatus* Erichson, 1839

*Stenus juno* (Paykull, 1789)

*Stenus lustrator* Erichson, 1839

*Stenus morio* Gravenhorst, 1806

*Stenus nanus* Stephens, 1833

*Stenus pubescens pubescens* Stephens, 1833

*Stenus similis* (Herbst, 1784)

*Stenus solutus* Erichson, 1840

*Stenus sylvester* Erichson, 1839

**Subfamily EUAESTHETINAE C.G. Thomson, 1859**

**Tribe Euaesthetini C.G. Thomson, 1859**

*Euaesthetus ruficapillus* (Lacordaire, 1835)

**Subfamily PAEDERINAE Fleming, 1821**

**Tribe Paederini Fleming, 1821**

Subtribe Astenina Hatch, 1957

Astenus (Astenus) gracilis (Paykull, 1789)

Astenus (Astenus) pulchellus (Heer, 1839)

Subtribe Cryptobiina Casey, 1905

*Ochthephilum fracticorne* (Paykull, 1800)

Subtribe Lathrobiina Laporte, 1835

*Achenium humile humile* (Nicolai, 1822)

Lathrobium (Lathrobium) brunnipes (Fabricius, 1792)

Lathrobium (Lathrobium) flavipes Hochhuth, 1851

Lathrobium (Lathrobium) fovulum Stephens, 1833

Lathrobium (Lathrobium) fulvipenne (Gravenhorst, 1806)

Lathrobium (Lathrobium) geminum Kraatz, 1857

Lathrobium (Lathrobium) longulum Gravenhorst, 1802

Lathrobium (Lathrobium) rufipenne Gyllenhal, 1813

*Tetartopeus quadratus* (Paykull, 1789)

*Tetartopeus rufonitidus* (Reitter, 1909)

*Tetartopeus terminatus* (Gravenhorst, 1802)

Subtribe Medonina Casey, 1905

*Lithocharis nigriceps* Kraatz, 1859

*Pseudomedon obscurellus* (Erichson, 1840)

Subtribe Paederina Fleming, 1821

Paederus (Heteropaederus) fuscipes fuscipes Curtis, 1826

Paederus (Paederus) riparius (Linnaeus, 1758)

*Paederus (Poederomorphus) littoralis littoralis* Gravenhorst, 1802

Subtribe Scopaeina Mulsant & Rey, 1878

Scopaeus (Scopaeus) laevigatus (Gyllenhal, 1827)

Subtribe Stilicina Casey, 1905

Rugilus (Rugilus) angustatus (Geoffroy, 1785)

Rugilus (Rugilus) erichsonii (Fauvel, 1867)

Rugilus (Rugilus) rufipes Germar, 1836

**Subfamily STAPHYLININAE Latreille, 1802**

**Tribe Othiini C.G. Thomson, 1859**

*Atrecus affinis* (Paykull, 1789)

*Othius punctulatus* (Goeze, 1777)

**Tribe Staphylinini Latreille, 1802**

Subtribe Amblyopinina Seevers, 1944

*Heterothops quadripunctulus* (Gravenhorst, 1806)

*Heterothops stiglundbergi* Israelson, 1979

Subtribe Philonthina Kirby, 1837

*Bisnius cephalotes* (Gravenhorst, 1802)*

*Bisnius fimetarius* (Gravenhorst, 1802)

*Bisnius nitidulus* (Gravenhorst, 1802)

*Bisnius puella* (Nordmann, 1837)

*Bisnius sordidus* (Gravenhorst, 1802)

*Bisnius subuliformis* (Gravenhorst, 1802)

Erichsonius (Erichsonius) cinerascens (Gravenhorst, 1802)

*Gabrius appendiculatus* Sharp, 1910

*Gabrius austriacus* Scheerpeltz, 1947

*Gabrius bescidicus* Smetana, 1954

*Gabrius breviventer* (Sperk, 1835)

*Gabrius exspectatus* Smetana, 1952

*Gabrius osseticus* (Kolenati, 1846)

*Gabrius trossulus* (Nordmann, 1837)

*Neobisnius procerulus procerulus* (Gravenhorst, 1806)

*Neobisnius villosulus* (Stephens, 1833)

Philonthus (Onychophilonthus) marginatus (O.F. Müller, 1764)

Philonthus (Philonthus) addendus Sharp, 1867

Philonthus (Philonthus) albipes (Gravenhorst, 1802)

Philonthus (Philonthus) atratus (Gravenhorst, 1802)

Philonthus (Philonthus) carbonarius (Gravenhorst, 1802)

Philonthus (Philonthus) cognatus Stephens, 1832

Philonthus (Philonthus) concinnus (Gravenhorst, 1802)

Philonthus (Philonthus) corvinus Erichson, 1839

Philonthus (Philonthus) cruentatus (Gmelin, 1790)

Philonthus (Philonthus) cyanipennis (Fabricius, 1792)

Philonthus (Philonthus) debilis (Gravenhorst, 1802)

Philonthus (Philonthus) decorus (Gravenhorst, 1802)

Philonthus (Philonthus) fumarius (Gravenhorst, 1806)

Philonthus (Philonthus) furcifer Renkonen, 1937

Philonthus (Philonthus) lepidus (Gravenhorst, 1802)

Philonthus (Philonthus) micans (Gravenhorst, 1802)

Philonthus (Philonthus) micantoides G. Benick & Lohse, 1956

Philonthus (Philonthus) nigrita (Gravenhorst, 1806)

Philonthus (Philonthus) politus (Linnaeus, 1758)

Philonthus (Philonthus) quisquiliarius quisquiliarius (Gyllenhal, 1810)

Philonthus (Philonthus) rectangulus Sharp, 1874†

Philonthus (Philonthus) rubripennis Stephens, 1832

Philonthus (Philonthus) sanguinolentus (Gravenhorst, 1802)

Philonthus (Philonthus) splendens splendens (Fabricius, 1792)

Philonthus (Philonthus) succicola C.G. Thomson, 1860

Philonthus (Philonthus) tenuicornis Mulsant & Rey, 1853

Philonthus (Philonthus) umbratilis (Gravenhorst, 1802)

Philonthus (Philonthus) varians (Paykull, 1789)

*Rabigus pullus* (Nordmann, 1837)

*Rabigus tenuis* (Fabricius, 1792)

Subtribe Quediina Kraatz, 1857

*Acylophorus wagenschieberi* Kiesenwetter, 1850

Quedius (Microsaurus) cruentus (G.-A. Olivier, 1795)

Quedius (Microsaurus) longicornis Kraatz, 1857*

Quedius (Microsaurus) maurus (C.R. Sahlberg, 1830)

Quedius (Microsaurus) mesomelinus mesomelinus (Marsham, 1802)

Quedius (Microsaurus) scitus (Gravenhorst, 1806)

Quedius (Microsaurus) xanthopus Erichson, 1839

Quedius (Quedius) fuliginosus (Gravenhorst, 1802)

Quedius (Quedius) molochinus (Gravenhorst, 1806)

Quedius (Velleius) dilatatus (Fabricius, 1787)

Subtribe Staphylinina Latreille, 1802

*Creophilus maxillosus maxillosus* (Linnaeus, 1758)

Dinothenarus (Dinothenarus) pubescens pubescens (De Geer, 1774)

*Emus hirtus* (Linnaeus, 1758)

Ocypus (Matidus) nitens nitens (Schrank, 1781)

Ocypus (Ocypus) ophthalmicus ophthalmicus (Scopoli, 1763)

*Ontholestes murinus* (Linnaeus, 1758)

*Ontholestes tessellatus* (Geoffroy, 1785)

*Ontholestes tessellatus* (Geoffroy, 1785)

Platydracus (Platydracus) fulvipes (Scopoli, 1763)

Platydracus (Platydracus) latebricola (Gravenhorst, 1806)*

Platydracus (Platydracus) stercorarius stercorarius (G.-A. Olivier, 1795)

*Staphylinus erythropterus erythropterus* Linnaeus, 1758

Subtribe Tanygnathinina Reitter, 1909

*Quedionuchus plagiatus* (Mannerheim, 1843)

**Tribe Xantholinini Erichson, 1839**

Gyrohypnus (Gyrohypnus) angustatus Stephens, 1833

Gyrohypnus (Gyrohypnus) fracticornis (O.F. Müller, 1776)

*Hypnogyra angularis* (Ganglbauer, 1895)

*Leptacinus intermedius* Donisthorpe, 1936

*Leptacinus sulcifrons* (Stephens, 1833)

*Nudobius lentus* (Gravenhorst, 1806)

Xantholinus (Purrolinus) tricolor (Fabricius, 1787)

Xantholinus (Xantholinus) dvoraki Coiffait, 1956

Xantholinus (Xantholinus) linearis linearis (G.-A. Olivier, 1795)

Xantholinus (Xantholinus) longiventris Heer, 1839

**Family SCYDMAENIDAE Leach, 1815**

**Supertribe SCYDMAENITAE Leach, 1815**

**Tribe Eutheiini Casey, 1897**

*Eutheia scydmaenoides scydmaenoides* Stephens, 1830

**Tribe Stenichnini Fauvel, 1885**

Euconnus (Euconnus) hirticollis (Illiger, 1798)

Euconnus (Napochus) claviger claviger (P.W.J. Müller & Kunze, 1822)

Euconnus (Neonapochus) maklinii (Mannerheim, 1844)

Euconnus (Psomophus) wetterhallii (Gyllenhal, 1813)

Neuraphes (Neuraphes) angulatus (P.W.J. Müller & Kunze, 1822)

Neuraphes (Neuraphes) elongatulus (P.W.J. Müller & Kunze, 1822)

Stenichnus (Stenichnus) bicolor (Denny, 1825)

Stenichnus (Stenichnus) collaris collaris (P.W.J. Müller & Kunze, 1822)

Stenichnus (Stenichnus) scutellaris (P.W.J. Müller & Kunze, 1822)

**Tribe Scydmaenini Leach, 1815**

Scydmaenus (Cholerus) hellwigii (Herbst, 1791)

Scydmaenus (Scydmaenus) tarsatus P.W.J. Müller & Kunze, 1822

**Series SCARABAEIFORMIA Crowson, 1960**

**Superfamily SCARABAEOIDEA Latreille, 1802**

**Family GEOTRUPIDAE Latreille, 1802**

**Subfamily GEOTRUPINAE Latreille, 1802**

**Tribe Geotrupini Latreille, 1802**

*Anoplotrupes stercorosus* (L.G. Scriba, 1791)

*Geotrupes baicalicus* Reitter, 1892

Trypocopris (Trypocopris) vernalis vernalis (Linnaeus, 1758)

**Family TROGIDAE W.S. MacLeay, 1819**

**Subfamily TROGINAE W.S. MacLeay, 1819**

*Trox cadaverinus cadaverinus* Illiger, 1802

*Trox sabulosus sabulosus* (Linnaeus, 1758)

*Trox scaber* (Linnaeus, 1767)

**Family LUCANIDAE Latreille, 1804**

**Subfamily SYNDESINAE W.S. MacLeay, 1819**

**Tribe Ceruchini LeConte, 1861**

*Ceruchus chrysomelinus* (Hochenwarth, 1785)

**Tribe Sinodendrini LeConte, 1861**

*Sinodendron cylindricum* (Linnaeus, 1758)

**Subfamily LUCANINAE Latreille, 1804**

**Tribe Platycerini Mulsant, 1842**

*Platycerus caprea* (De Geer, 1774)

*Platycerus caraboides* (Linnaeus, 1758)

**Family SCARABAEIDAE Latreille, 1802**

**Subfamily APHODIINAE Leach, 1815**

**Tribe Aphodiini Leach, 1815**

Subtribe Aphodiina Leach, 1815

*Acanthobodilus immundus* (Creutzer, 1799)

*Acrossus depressus* (Kugelann, 1792)

*Acrossus luridus* (Fabricius, 1775)

*Acrossus rufipes* (Linnaeus, 1758)

*Agoliinus nemoralis* (Erichson, 1848)

*Agrilinus ater* (De Geer, 1774)

*Ammoecius brevis* (Erichson, 1848)*

*Aphodius fimetarius* (Linnaeus, 1758)

*Bodilopsis rufa* (Moll, 1782)

*Bodilopsis sordida sordida* (Fabricius, 1775)

*Bodilus lugens* (Creutzer, 1799)

*Calamosternus granarius* (Linnaeus, 1767)

*Chilothorax distinctus distinctus* (O.F. Müller, 1776)

*Chilothorax melanosticus* (W.L.E. Schmidt, 1840)

*Colobopterus erraticus* (Linnaeus, 1758)

*Esymus pusillus pusillus* (Herbst, 1789)

*Euheptaulacus sus* (Herbst, 1783)

*Eupleurus subterraneus subterraneus* (Linnaeus, 1758)

*Liothorax plagiatus* (Linnaeus, 1767)

*Melinopterus prodromus* (Brahm, 1790)

*Melinopterus punctatosulcatus hirtipes* (Fischer von Waldheim, 1844)

*Mendidaphodius linearis* (Reiche & Saulcy, 1856)

*Nialus varians* (Duftschmid, 1805)

*Otophorus haemorrhoidalis* (Linnaeus, 1758)

*Oxyomus sylvestris* (Scopoli, 1763)

*Teuchestes fossor* (Linnaeus, 1758)

*Volinus sticticus* (Panzer, 1798)

**Tribe Psammodiini Mulsant, 1842**

**Subtribe Rhyssemina Pittino & Mariani, 1986**

*Pleurophorus caesus* (Creutzer, 1796)

**Subfamily SCARABAEINAE Latreille, 1802**

**Tribe Coprini Leach, 1815**

Copris (Copris) lunaris (Linnaeus, 1758)

**Tribe Oniticellini H.J. Kolbe, 1905**

Subtribe Oniticellina H.J. Kolbe, 1905

*Euoniticellus fulvus* (Goeze, 1777)

**Tribe Onthophagini Streubel, 1846**

Caccobius (Caccobius) schreberi (Linnaeus, 1767)

Onthophagus (Furconthophagus) furcatus (Fabricius, 1781)

Onthophagus (Palaeonthophagus) coenobita (Herbst, 1783)

Onthophagus (Palaeonthophagus) fracticornis (Preyssler, 1790)

Onthophagus (Palaeonthophagus) gibbulus gibbulus (Pallas, 1781)

Onthophagus (Palaeonthophagus) nuchicornis (Linnaeus, 1758)

Onthophagus (Palaeonthophagus) ovatus (Linnaeus, 1767)

Onthophagus (Palaeonthophagus) vacca (Linnaeus, 1767)

**Subfamily MELOLONTHINAE Leach, 1819**

**Tribe Hopliini Latreille, 1829**

Hoplia (Hoplia) parvula Krynicki, 1832

Hoplia (Hoplia) zaitzevi Jakobson, 1914* {ZIN}

**Tribe Melolonthini Leach, 1819**

*Melolontha hippocastani hippocastani* Fabricius, 1801

**Tribe Rhizotrogini Burmeister, 1855**

*Amphimallon altaicum* (Mannerheim, 1825)

*Amphimallon solstitiale solstitiale* (Linnaeus, 1758)

**Tribe Sericini Kirby, 1837**

Maladera (Maladera) holosericea (Scopoli, 1772)

Serica (Serica) brunnea (Linnaeus, 1758)

**Subfamily RUTELINAE W.S. MacLeay, 1819**

**Tribe Anomalini Streubel, 1839**

Subtribe Anisopliina Burmeister, 1844

*Chaetopteroplia segetum segetum* (Herbst, 1783)

Subtribe Anomalina Streubel, 1839

*Anomala dubia dubia* (Scopoli, 1763)

*Mimela holosericea* (Fabricius, 1787)

*Phyllopertha horticola* (Linnaeus, 1758)

**Subfamily DYNASTINAE W.S. MacLeay, 1819**

**Tribe Oryctini Mulsant, 1842**

Oryctes (Oryctes) nasicornis polonicus Minck, 1918

**Subfamily CETONIINAE Leach, 1815**

**Tribe Cetoniini Leach, 1815**

Subtribe Cetoniina Leach, 1815

Cetonia (Cetonia) aurata aurata (Linnaeus, 1758)

Protaetia (Cetonischema) speciosissima (Scopoli, 1786)

Protaetia (Liocola) marmorata marmorata (Fabricus, 1792)

Protaetia (Potosia) cuprea volhyniensis (Gory & Percheron, 1833)

[indicated as *Protaetia metallica* (Herbst, 1782) in our publications Egorov and Ruchin 2013b, 2014]

Protaetia (Potosia) fieberi boldyrevi Jakobson, 1909

Subtribe Leucocelina Kraatz, 1882

*Oxythyrea funesta* (Poda von Neuhaus, 1761)

**Tribe Osmodermatini Schenkling, 1922**

*Osmoderma barnabita* Motschulsky, 1845

**Tribe Trichiini Fleming, 1821**

Subtribe Trichiina Fleming, 1821

*Gnorimus variabilis* (Linnaeus, 1758)

*Trichius fasciatus* (Linnaeus, 1758)

**Tribe Valgini Mulsant, 1842**

*Valgus hemipterus hemipterus* (Linnaeus, 1758)

**Series SCIRTIFORMIA Fleming, 1821**

**Superfamily SCIRTOIDEA Fleming, 1821**

**Family SCIRTIDAE Fleming, 1821**

**Subfamily SCIRTINAE Fleming, 1821**

*Contacyphon padi* (Linnaeus, 1758)

*Contacyphon pubescens* (Fabricius, 1792)

*Contacyphon variabilis* (Thunberg, 1787)

*Elodes minutus* (Linnaeus, 1767)

*Microcara testacea* (Linnaeus, 1767)

*Scirtes hemisphaericus* (Linnaeus, 1758)

**Family EUCINETIDAE Lacordaire, 1857**

*Eucinetus haemorrhoidalis* (Germar, 1818)

**Series ELATERIFORMIA Crowson, 1960**

**Superfamily DASCILLOIDEA Guérin-Méneville, 1843 (1834)**

**Family DASCILLIDAE Guérin-Méneville, 1843 (1834)**

**Subfamily DASCILLINAE Guérin-Méneville, 1843 (1834)**

**Tribe Dascillini Guérin-Méneville, 1843 (1834)**

*Dascillus cervinus* (Linnaeus, 1758)

**Superfamily BUPRESTOIDEA Leach, 1815**

**Family BUPRESTIDAE Leach, 1815**

**Subfamily CHRYSOCHROINAE Laporte, 1835**

**Tribe Chalcophorini Lacordaire, 1857**

*Chalcophora mariana* (Linnaeus, 1758)

**Tribe Dicercini Gistel, 1848**

*Dicerca aenea aenea* (Linnaeus, 1760)

*Dicerca alni* (Fischer von Waldheim, 1824)

*Dicerca furcata* (Thunberg, 1787)

**Tribe Poecilonotini Jakobson, 1913**

*Poecilonota variolosa variolosa* (Paykull, 1799)

**Subfamily BUPRESTINAE Leach, 1815**

**Tribe Anthaxiini Gory & Laporte, 1839**

Anthaxia (Melanthaxia) quadripunctata quadripunctata (Linnaeus, 1758)

**Tribe Buprestini Leach, 1815**

Buprestis (Ancylocheira) haemorrhoidalis haemorrhoidalis Herbst, 1780

Buprestis (Ancylocheira) rustica rustica Linnaeus, 1758

Buprestis (Buprestis) octoguttata octoguttata Linnaeus, 1758

**Tribe Chrysobothrini Gory & Laporte, 1837**

Chrysobothris (Chrysobothris) affinis affinis (Fabricius, 1784)

Chrysobothris (Chrysobothris) chrysostigma chrysostigma (Linnaeus, 1758)

**Tribe Melanophilini Bedel, 1921**

*Melanophila acuminata* (De Geer, 1774)

*Phaenops cyanea* (Fabricius, 1775)

**Subfamily AGRILINAE Laporte, 1835**

**Tribe Agrilini Laporte, 1835**

Subtribe Agrilina Laporte, 1835

*Agrilus angustulus angustulus* (Illiger, 1803)*

*Agrilus ater* (Linnaeus, 1767)*

*Agrilus betuleti* (Ratzeburg, 1837)

*Agrilus biguttatus* (Fabricius, 1777)

*Agrilus cuprescens cuprescens* (Ménétriés, 1832)

*Agrilus cyanescens cyanescens* (Ratzeburg, 1837)

*Agrilus kaluganus* Obenberger, 1940 {ZIN}

*Agrilus pratensis* (Ratzeburg, 1837)

*Agrilus salicis* J. Frivaldszky, 1877

*Agrilus sulcicollis* Lacordaire, 1835

*Agrilus viridis* (Linnaeus, 1758)

*Agrilus zigzag* Marseul, 1866

**Tribe Coraebini Bedel, 1921**

Subtribe Coraebina Bedel, 1921

*Coraebus elatus* (Fabricius, 1787)

**Tribe Tracheini Laporte, 1835**

Subtribe Tracheina Laporte, 1835

*Trachys minutus minutus* (Linnaeus, 1758)

**Superfamily BYRRHOIDEA Latreille, 1804**

**Family BYRRHIDAE Latreille, 1804**

**Subfamily BYRRHINAE Latreille, 1804**

**Tribe Morychini El Moursy, 1961**

*Morychus aeneus* (Fabricius, 1775)

*Lamprobyrrhulus nitidus* (Schaller, 1783)

**Tribe Byrrhini Latreille, 1804**

Byrrhus (Byrrhus) fasciatus (Forster, 1771)

Byrrhus (Byrrhus) pilula pilula (Linnaeus, 1758)

Byrrhus (Byrrhus) pustulatus pustulatus (Forster, 1771)

*Cytilus sericeus* (Forster, 1771)

**Subfamily SYNCALYPTINAE Mulsant & Rey, 1869**

**Tribe Syncalyptini Mulsant & Rey, 1869**

Curimopsis (Curimopsis) paleata (Erichson, 1846)

**Family ELMIDAE Curtis, 1830**

**Subfamily ELMINAE Curtis, 1830**

**Tribe Macronychini Gistel, 1848**

*Macronychus quadrituberculatus* P.W.J. Müller, 1806

**Family DRYOPIDAE Billberg, 1820 (1817)**

*Dryops auriculatus* (Geoffroy, 1785)

*Dryops ernesti* Gozis, 1886

**Family LIMNICHIDAE Erichson, 1846**

**Subfamily LIMNICHINAE Erichson, 1846**

*Limnichus sericeus* (Duftschmid, 1825)

**Family HETEROCERIDAE W.S. MacLeay, 1825**

**Subfamily HETEROCERINAE W.S. MacLeay, 1825**

**Tribe Augylini Pacheco, 1964**

Augyles (Augyles) hispidulus (Kiesenwetter, 1843)

**Tribe Heterocerini W.S. MacLeay, 1825**

*Heterocerus fenestratus* (Thunberg, 1784)

*Heterocerus fossor* Kiesenwetter, 1843

*Heterocerus fusculus fusculus* Kiesenwetter, 1843

*Heterocerus marginatus* (Fabricius, 1787)

**Superfamily ELATEROIDEA Leach, 1815**

**Family EUCNEMIDAE Eschscholtz, 1829**

**Subfamily MELASINAE Fleming, 1821**

**Tribe Calyptocerini Muona, 1993**

*Otho sphondyloides* (Germar, 1818) {ZIN}

**Tribe Dirhagini Reitter, 1911**

*Microrhagus emyi* (Rouget, 1856) {ZIN}

*Microrhagus lepidus* (Rosenhauer, 1847) {ZIN}

*Microrhagus pygmaeus* (Fabricius, 1792) {ZIN}

*Dirrhagofarsus attenuatus* (Mäklin, 1845) {ZIN}

*Rhacopus sahlbergi* (Mannerheim, 1823) {ZIN}

**Tribe Epiphanini Muona, 1993**

*Hylis olexai* (Palm, 1955) {ZIN}

*Hylis procerulus* (Mannerheim, 1823) {ZIN}

**Tribe Melasini Fleming, 1821**

*Isorhipis marmottani* (Bonvouloir, 1871) {ZIN}

*Isorhipis melasoides* (Laporte, 1835)* {ZIN}

*Melasis buprestoides* (Linnaeus, 1760) {ZIN}

**Subfamily EUCNEMINAE Eschscholtz, 1829**

**Tribe Eucnemini Eschscholtz, 1829**

*Eucnemis zaitzevi* Mamaev, 1976* {ZIN}

[mistakenly indicated as *Eucnemis capucina* Ahrens, 1812 (Egorov et al. 2016, Kovalev and Egorov 2017)]

**Tribe Euryptychini Mamaev, 1976**

*Dromaeolus barnabita* (A. Villa & G.B. Villa, 1838) {ZIN}

**Family THROSCIDAE Laporte, 1840**

**Subfamily THROSCINAE Laporte, 1840**

**Tribe Throscini Laporte, 1840**

*Trixagus dermestoides* (Linnaeus, 1767) {ZIN}

**Family ELATERIDAE Leach, 1815**

**Subfamily AGRYPNINAE Candèze, 1857**

**Tribe Agrypnini Candèze, 1857**

*Agrypnus murinus* (Linnaeus, 1758)

*Danosoma conspersum* (Gyllenhal, 1808)

*Danosoma fasciatum* (Linnaeus, 1758)

*Lacon lepidopterus* (Panzer, 1800) {ZMMU}

**Subfamily CARDIOPHORINAE Candèze, 1859**

**Tribe Cardiophorini Candèze, 1859**

Cardiophorus (Cardiophorus) ebeninus (Germar, 1823)

Cardiophorus (Cardiophorus) ruficollis (Linnaeus, 1758)

*Dicronychus equiseti* (Herbst, 1784)

**Subfamily DENDROMETRINAE Gistel, 1848**

**Tribe Dendrometrini Gistel, 1848**

Subtribe Dendrometrina Gistel, 1848

Athous (Athous) haemorrhoidalis (Fabricius, 1801)

Athous (Athous) vittatus (Fabricius, 1792)

Athous (Haplathous) subfuscus (O.F. Müller, 1764)

*Limonius minutus* (Linnaeus, 1758)

*Pheletes aeneoniger* (De Geer, 1774)

Subtribe Denticollina Stein & J. Weise, 1877 (1848)

*Denticollis borealis* (Paykull, 1800) {ZIN}

*Denticollis linearis* (Linnaeus, 1758)

*Denticollis rubens* Piller et Mitterpacher, 1783* {ZIN}

Subtribe Hemicrepidiina Champion, 1894

*Diacanthous undulatus* (De Geer, 1774)

Hemicrepidius (Hemicrepidius) hirtus (Herbst, 1784)

Hemicrepidius (Hemicrepidius) niger (Linnaeus, 1758)

**Tribe Hypnoidini Schwarz, 1906 (1860)**

*Hypnoidus riparius* (Fabricius, 1792)

**Tribe Prosternini Gistel, 1856**

Actenicerus (Actenicerus) sjaelandicus (O.F. Müller, 1764)

*Anostirus castaneus castaneus* (Linnaeus, 1758)

*Aplotarsus incanus* (Gyllenhal, 1827)

*Ctenicera pectinicornis* (Linnaeus, 1758)

*Orithales serraticornis serraticornis* (Paykull, 1800)

*Prosternon tessellatum* (Linnaeus, 1758)

? *Pseudanostirus globicollis* (Germar, 1843) {Kurmaeva et al. 2008}

**Tribe Selatosomini Schimmel, Tarnawski, Han et Platia, 2015**

Subtribe Mosotalesina Schimmel, Tarnawski, Han et Platia, 2015

Mosotalesus (Mosotalesus) impressus impressus (Fabricius, 1792)

Mosotalesus (Mosotalesus) nigricornis (Panzer, 1799)

Subtribe Selatosomina Schimmel, Tarnawski, Han et Platia, 2015

*Pristilophus cruciatus* (Linnaeus, 1758)

Selatosomus (Selatosomus) aeneus (Linnaeus, 1758)

Selatosomus (Selatosomus) latus (Fabricius, 1801)

**Subfamily ELATERINAE Leach, 1815**

**Tribe Agriotini Laporte, 1840**

Subtribe Agriotina Laporte, 1840

Agriotes (Agriotes) lineatus (Linnaeus, 1767)

Agriotes (Agriotes) obscurus (Linnaeus, 1758)

Agriotes (Agriotes) sputator (Linnaeus, 1758)

*Dalopius marginatus* (Linnaeus, 1758)

*Ectinus aterrimus* (Linnaeus, 1760) {ZMMU}

**Tribe Ampedini Gistel, 1848**

Ampedus (Ampedus) balteatus (Linnaeus, 1758)

Ampedus (Ampedus) cinnabarinus (Eschscholtz, 1829)

Ampedus (Ampedus) elegantulus (Schönherr, 1817)

Ampedus (Ampedus) elongatulus (Fabricius, 1787) {ZMMU}

Ampedus (Ampedus) erythrogonus (P.W. Müller, 1821)

Ampedus (Ampedus) karpathicus (Buysson, 1886) {ZMMU}

Ampedus (Ampedus) nigerrimus (Lacordaire in Boisduval & Lacordaire, 1835) {ZMMU}

Ampedus (Ampedus) nigrinus (Herbst, 1784)

Ampedus (Ampedus) nigroflavus (Goeze, 1777)

Ampedus (Ampedus) pomonae (Stephens, 1830)

Ampedus (Ampedus) pomorum (Herbst, 1784)

Ampedus (Ampedus) praeustus (Fabricius, 1792)

Ampedus (Ampedus) sanguineus (Linnaeus, 1758)

Ampedus (Ampedus) sanguinolentus sanguinolentus (Schrank, 1776)

Ampedus (Ampedus) tristis (Linnaeus, 1758)

**Tribe Elaterini Leach, 1815**

*Elater ferrugineus ferrugineus* Linnaeus, 1758

Sericus (Sericus) brunneus brunneus (Linnaeus, 1758)

Sericus (Sericus) sulcipennis Buysson, 1893

**Tribe Melanotini Candèze, 1859 (1848)**

Melanotus (Melanotus) castanipes (Paykull, 1800)

Melanotus (Melanotus) villosus (Geoffroy, 1785)

**Tribe Synaptini Gistel, 1856**

*Synaptus filiformis* (Fabricius, 1781)

**Subfamily NEGASTRIINAE Nakane & Kishii, 1956**

**Tribe Negastriini Nakane & Kishii, 1956**

*Negastrius pulchellus* (Linnaeus, 1760)

*Oedostethus quadripustulatus* (Fabricius, 1792)

**Family LYCIDAE Laporte, 1838**

**Subfamily EROTINAE LeConte, 1881**

**Tribe Erotini LeConte, 1881**

*Aplatopterus rubens* (Gyllenhal, 1817)

Erotides (Glabroplatycis) nasutus (Kiesenwetter, 1874)

*Lopheros lineatus* (Gorham, 1883) {ZIN, ZMMU},

*Platycis minutus* (Fabricius, 1787)

**Tribe Dictyopterini Houlbert, 1922**

Subtribe Dictyopterina Houlbert, 1922

*Dictyoptera aurora* (Herbst, 1784)

*Pyropterus nigroruber* (De Geer, 1774)

**Tribe Conderini Bocák et Bocáková, 1990**

*Xylobanellus erythropterus* (Baudi di Selve, 1872)

**Subfamily LYCINAE Laporte, 1838**

**Tribe Calochromini Lacordaire, 1857**

*Lygistopterus sanguineus* (Linnaeus, 1758)

**Family LAMPYRIDAE Rafinesque, 1815**

**Subfamily LAMPYRINAE Rafinesque, 1815**

**Tribe Lampyrini Rafinesque, 1815**

*Lampyris noctiluca* (Linnaeus, 1758)

**Family CANTHARIDAE Imhoff, 1856 (1815)**

**Subfamily CANTHARINAE Imhoff, 1856 (1815)**

**Tribe Podabrini Gistel, 1856**

*Podabrus alpinus* (Paykull, 1798)

**Tribe Cantharini Imhoff, 1856 (1815)**

Cantharis (Cantharis) figurata Mannerheim, 1843

Cantharis (Cantharis) flavilabris Fallén, 1807

Cantharis (Cantharis) fusca Linnaeus, 1758

Cantharis (Cantharis) livida Linnaeus, 1758

Cantharis (Cantharis) nigricans O.F. Müller, 1776

Cantharis (Cantharis) obscura Linnaeus, 1758

Cantharis (Cantharis) pallida Goeze, 1777

Cantharis (Cantharis) paludosa Fallén, 1807

Cantharis (Cantharis) pellucida Fabricius, 1792

Cantharis (Cantharis) rufa Linnaeus, 1758

Cantharis (Cantharis) rustica Fallén, 1807

Cantharis (Cyrtomoptila) lateralis Linnaeus, 1758

Rhagonycha (Rhagonycha) atra (Linnaeus, 1767)

Rhagonycha (Rhagonycha) elongata (Fallén, 1807)

Rhagonycha (Rhagonycha) fugax fugax Mannerheim, 1843

Rhagonycha (Rhagonycha) fulva (Scopoli, 1763)

Rhagonycha (Rhagonycha) lignosa (O.F. Müller, 1764)

Rhagonycha (Rhagonycha) nigripes (W. Redtenbacher, 1842)

Rhagonycha (Rhagonycha) nigriventris Motschulsky, 1860

Rhagonycha (Rhagonycha) testacea (Linnaeus, 1758)

**Subfamily SILINAE Mulsant, 1862**

**Tribe Silini Mulsant, 1862**

*Silis ruficollis* (Fabricius, 1775)

**Subfamily MALTHININAE Kiesenwetter, 1852**

**Tribe Malthinini Kiesenwetter, 1852**

Malthinus (Malthinus) fasciatus (G.-A. Olivier, 1790)

Malthinus (Malthinus) flaveolus (Herbst, 1786)

Malthinus (Malthinus) frontalis (Marsham, 1802)

**Tribe Malthodini Böving & Craighead, 1931**

Malthodes (Malthodes) guttifer Kiesenwetter, 1852*

**Series BOSTRICHIFORMIA Forbes, 1926**

**Superfamily BOSTRICHOIDEA Latreille, 1802**

**Family DERMESTIDAE Latreille, 1804**

**Subfamily DERMESTINAE Latreille, 1804**

**Tribe Dermestini Latreille, 1804**

Dermestes (Dermestes) lardarius Linnaeus, 1758†

Dermestes (Dermestinus) frischii Kugelann, 1792†

Dermestes (Dermestinus) laniarius Illiger, 1801

Dermestes (Dermestinus) murinus murinus Linnaeus, 1758

Dermestes (Dermestinus) undulatus Brahm, 1790*

**Subfamily ORPHILINAE LeConte, 1861**

*Orphilus niger* (P. Rossi, 1790)

**Subfamily ATTAGENINAE Laporte, 1840**

**Tribe Attagenini Laporte, 1840**

Attagenus (Attagenus) schaefferi schaefferi (Herbst, 1792)

Attagenus (Attagenus) unicolor unicolor (Brahm, 1790)†

**Subfamily MEGATOMINAE Leach, 1815**

**Tribe Anthrenini Gistel, 1848**

Anthrenus (Anthrenus) scrophulariae scrophulariae (Linnaeus, 1758)

Anthrenus (Florilinus) museorum (Linnaeus, 1760)

**Tribe Megatomini Leach, 1815**

Ctesias (Ctesias) serra (Fabricius, 1792)

? Globicornis (Hadrotoma) corticalis (Eichhoff, 1863) {Egorov and Ruchin 2014}

Globicornis (Hadrotoma) emarginata (Gyllenhal, 1808)

Megatoma (Megatoma) undata undata (Linnaeus, 1758)

*Trogoderma glabrum* (Herbst, 1783)†

**Family BOSTRICHIDAE Latreille, 1802**

**Subfamily BOSTRICHINAE Latreille, 1802**

**Tribe Bostrichini Latreille, 1802**

*Bostrichus capucinus* (Linnaeus, 1758)

**Subfamily DINODERINAE C.G. Thomson, 1863**

*Stephanopachys linearis* (Kugelann, 1792)

**Family PTINIDAE Latreille, 1802**

**Subfamily PTININAE Latreille, 1802**

**Tribe Ptinini Latreille, 1802**

Ptinus (Bruchoptinus) rufipes G.-A. Olivier, 1790

Ptinus (Cyphoderes) raptor Sturm, 1837

Ptinus (Ptinus) fur (Linnaeus, 1758)†

Ptinus (Ptinus) villiger (Reitter, 1884)

**Subfamily ANOBIINAE Fleming, 1821**

**Tribe Anobiini Fleming, 1821**

*Anobium punctatum* (De Geer, 1774) {personal collection of S.K. Alekseev, Kaluga}

*Cacotemnus rufipes* (Fabricius, 1792)

*Hadrobregmus pertinax* (Linnaeus, 1758)

*Priobium carpini* (Herbst, 1793)

*Stegobium paniceum* (Linnaeus, 1758)†

**Subfamily DORCATOMINAE C**.**G. Thomson, 1859**

**Tribe Dorcatomini C**.**G. Thomson, 1859**

*Caenocara affine* (Sturm, 1837)

Dorcatoma (Dorcatoma) dresdensis Herbst, 1792

Dorcatoma (Dorcatoma) robusta A. Strand, 1938

Dorcatoma (Pilosodorcatoma) chrysomelina Sturm, 1837

Dorcatoma (Sternitodorcatoma) flavicornis (Fabricius, 1792)

**Subfamily ERNOBIINAE Pic, 1912**

**Tribe Ernobiini Pic, 1912**

*Ernobius explanatus explanatus* (Mannerheim, 1843)

*Ernobius longicornis* (Sturm, 1837)

**Subfamily PTILININAE Shuckard, 1839**

**Tribe Ptilinini Shuckard, 1839**

*Ptilinus fuscus* (Geoffroy, 1785)

**Subfamily XYLETININAE Gistel, 1848**

**Tribe Xyletinini Gistel, 1848**

Xyletinus (Xyletinus) longitarsis longitarsis Jansson, 1942

Xyletinus (Xyletinus) pectinatus pectinatus (Fabricius, 1792)

**Series CUCUJIFORMIA Lameere, 1938**

**Superfamily LYMEXYLOIDEA Fleming, 1821**

**Family LYMEXYLIDAE Fleming, 1821**

**Subfamily HYLECOETINAE Germar, 1818**

*Elateroides dermestoides* (Linnaeus, 1760)

**Subfamily LYMEXYLINAE Fleming, 1821**

*Lymexylon navale* (Linnaeius, 1758)

**Superfamily CLEROIDEA Latreille, 1802**

**Family BIPHYLLIDAE LeConte, 1861**

*Biphyllus lunatus* (Fabricius, 1787)

*Diplocoelus fagi* (Chevrolat, 1837)

**Family BYTURIDAE Gistel, 1848**

**Subfamily BYTURINAE Gistel, 1848**

*Byturus ochraceus* (L.G. Scriba, 1790)

*Byturus tomentosus* (De Geer, 1774)

**Family TROGOSSITIDAE Latreille, 1802**

**Subfamily PELTINAE Kirby, 1837**

**Tribe Lophocaterini Crowson, 1964**

*Grynocharis oblonga* (Linnaeus, 1758)

**Tribe Peltini Kirby, 1837**

*Peltis ferruginea* (Linnaeus, 1758)

*Peltis grossa* (Linnaeus, 1758)

**Tribe Thymalini Léveillé, 1888**

*Thymalus oblongus* Reitter, 1889

**Family CLERIDAE Latreille, 1802**

**Subfamily TILLINAE Fischer von Waldheim, 1813**

*Tillus elongatus* (Linnaeus, 1758)

**Subfamily CLERINAE Latreille, 1802**

*Allonyx quadrimaculatus* (Schaller, 1783) {ZIN}

*Thanasimus femoralis* (Zetterstedt, 1828)

*Thanasimus formicarius formicarius* (Linnaeus, 1758)

*Trichodes apiarius* (Linnaeus, 1758)

**Subfamily KORYNETINAE Laporte, 1838**

*Necrobia violacea* (Linnaeus, 1758)†

**Family MELYRIDAE Leach, 1815**

**Subfamily RHADALINAE LeConte, 1861**

Aplocnemus (Aplocnemus) nigricornis nigricornis (Fabricius, 1792)

**Subfamily DASYTINAE Laporte, 1840**

**Tribe Dasytini Laporte, 1840**

Dasytes (Dasytes) niger (Linnaeus, 1760)

Dasytes (Metadasytes) fusculus (Illiger, 1801)

*Dolichosoma lineare* (P. Rossi, 1794)

**Subfamily MALACHIINAE Fleming, 1821**

**Tribe Malachiini Fleming, 1821**

Anthocomus (Anthocomus) fasciatus (Linnaeus, 1758)

Anthocomus (Anthocomus) rufus rufus (Herbst, 1784)

Anthocomus (Celidus) equestris (Fabricius, 1781)

*Apalochrus femoralis* Erichson, 1840

*Charopus flavipes* (Paykull, 1798)

Clanoptilus (Clanoptilus) geniculatus (Germar, 1823)

*Cordylepherus viridis* (Fabricius, 1787)

Ebaeus (Ebaeus) pedicularius pedicularius (Linnaeus, 1758)

Malachius (Malachius) aeneus (Linnaeus, 1758)

Malachius (Malachius) bipustulatus (Linnaeus, 1758)

*Nepachys cardiacae* (Linnaeus, 1760)

**Superfamily CUCUJOIDEA Latreille, 1802**

**Family SPHINDIDAE Jacquelin du Val, 1860**

**Subfamily SPHINDINAE Jacquelin du Val, 1860**

*Sphindus dubius* (Gyllenhal, 1808)

**Subfamily ASPIDIPHORINAE Kiesenwetter, 1877**

*Aspidiphorus orbiculatus* (Gyllenhal, 1808)

**Family EROTYLIDAE Latreille, 1802**

**Subfamily EROTYLINAE Latreille, 1802**

**Tribe Dacnini Gistel, 1848**

*Combocerus glaber* (Schaller, 1783)*

Dacne (Dacne) bipustulata (Thunberg, 1781)

**Tribe Tritomini Curtis, 1834**

*Triplax aenea* (Schaller, 1783)

*Triplax collaris* (Schaller, 1783)

*Triplax lepida* (Faldermann, 1837)*

*Triplax rufipes* (Fabricius, 1787)

*Triplax russica* (Linnaeus, 1758)

*Triplax scutellaris* Charpentier, 1825

Tritoma (Tritoma) bipustulata Fabricius, 1775

Tritoma (Tritoma) subbasalis (Reitter, 1896)

**Family MONOTOMIDAE Laporte, 1840**

**Subfamily RHIZOPHAGINAE L. Redtenbacher, 1845**

Rhizophagus (Anomophagus) cribratus (Gyllenhal, 1827)

Rhizophagus (Eurhizophagus) depressus (Fabricius, 1792)

Rhizophagus (Rhizophagus) bipustulatus (Fabricius, 1792)

Rhizophagus (Rhizophagus) dispar (Paykull, 1800)

Rhizophagus (Rhizophagus) fenestralis (Linnaeus, 1758)

Rhizophagus (Rhizophagus) ferrugineus (Paykull, 1800)

Rhizophagus (Rhizophagus) nitidulus (Fabricius, 1798)

Rhizophagus (Rhizophagus) parallelocollis (Gyllenhal, 1827)

Rhizophagus (Rhizophagus) perforatus Erichson, 1845

Rhizophagus (Rhizophagus) picipes (G.-A. Olivier, 1790)

**Subfamily MONOTOMINAE Laporte, 1840**

*Monotoma picipes* Herbst, 1793

**Family KATERETIDAE Kirby, 1837**

*Brachypterolus linariae* (Stephens, 1830)

*Brachypterolus pulicarius* (Linnaeus, 1758)

*Brachypterus fulvipes* Erichson, 1843

*Brachypterus urticae* (Fabricius, 1792)

*Heterhelus scutellaris* (Heer, 1841)

*Kateretes pedicularius* (Linnaeus, 1758)

*Kateretes pusillus* (Thunberg, 1794)

**Family NITIDULIDAE Latreille, 1802**

**Subfamily EPURAEINAE Kirejtshuk, 1986**

**Tribe Epuraeini Kirejtshuk, 1986**

Epuraea (Epuraea) aestiva (Linnaeus, 1758)

Epuraea (Epuraea) longula Erichson, 1845 {personal collection of S.K. Alekseev, Kaluga}

Epuraea (Epuraea) marseuli Reitter, 1873

Epuraea (Epuraea) pallescens (Stephens, 1835) {personal collection of S.K. Alekseev, Kaluga}

Epuraea (Epuraea) variegata (Herbst, 1793)

Epuraea (Epuraeanella) neglecta (Heer, 1841) {personal collection of S.K. Alekseev, Kaluga}

**Subfamily CARPOPHILINAE Erichson, 1842**

Carpophilus (Carpophilus) hemipterus (Linnaeus, 1758)*†

**Subfamily CRYPTARCHINAE C.G. Thomson, 1859**

**Tribe Cryptarchini C.G. Thomson, 1859**

*Cryptarcha strigata* (Fabricius, 1787)

*Cryptarcha undata* (G.-A. Olivier, 1790)

Glischrochilus (Glischrochilus) quadripunctatus (Linnaeus, 1758)

Glischrochilus (Librodor) grandis (Tournier, 1872)

Glischrochilus (Librodor) hortensis (Geoffroy, 1785)

Glischrochilus (Librodor) quadriguttatus (Fabricius, 1777)*

Glischrochilus (Librodor) quadrisignatus (Say, 1835)*† {ZIN}

*Pityophagus ferrugineus* (Linnaeus, 1760)

**Subfamily NITIDULINAE Latreille, 1802**

**Tribe Cychramini Gistel, 1848**

*Cychramus luteus* (Fabricius, 1787)

*Cychramus variegatus* (Herbst, 1792)

**Tribe Cyllodini Everts, 1898**

*Cyllodes ater* (Herbst, 1792)

**Tribe Nitidulini Latreille, 1802**

*Amphotis marginata* (Fabricius, 1781)

Ipidia (Hemipidia) sexguttata (R.F. Sahlberg, 1834) {ZIN}

Ipidia (Ipidia) binotata Reitter, 1875

*Nitidula bipunctata* (Linnaeus, 1758)

*Nitidula carnaria* (Schaller, 1783)

*Nitidula rufipes* (Linnaeus, 1767)

*Omosita colon* (Linnaeus, 1758)

*Omosita depressa* (Linnaeus, 1758)

*Omosita discoidea* (Fabricius, 1775)†

*Omosita japonica* Reitter, 1874† {ZIN}

*Pocadius ferrugineus* (Fabricius, 1775)

*Soronia grisea* (Linnaeus, 1758)

*Soronia punctatissima* (Illiger, 1794)*

**Subfamily MELIGETHINAE C.G. Thomson, 1859**

Meligethes (Clypeogethes) aeneus (Fabricius, 1775)

Meligethes (Meligethes) flavimanus Stephens, 1830

**Family CRYPTOPHAGIDAE Kirby, 1826**

**Subfamily CRYPTOPHAGINAE Kirby, 1826**

**Tribe Caenoscelini Casey, 1900**

*Caenoscelis subdeplanata* C.N.F. Brisout de Barneville, 1882†

**Tribe Cryptophagini Kirby, 1826**

*Antherophagus pallens* (Linnaeus, 1758)

*Antherophagus silaceus* (Herbst, 1792)

*Antherophagus similis* Curtis, 1835

*Cryptophagus fallax* J. Balfour-Browne, 1953†

*Cryptophagus pilosus* Gyllenhal, 1827

*Cryptophagus hexagonalis* Tournier, 1872

*Henoticus serratus* (Gyllenhal, 1808)

*Pteryngium crenatum* (Fabricius, 1798)*

*Telmatophilus caricis* (G.-A. Olivier, 1790)

*Telmatophilus typhae* (Fallén, 1802)

**Subfamily ATOMARIINAE LeConte, 1861**

**Tribe Atomariini LeConte, 1861**

Atomaria (Anchicera) fuscata (Schönherr, 1808)

*Curelius exiguus* (Erichson, 1846)

*Ephistemus globulus* (Paykull, 1798)

**Family SILVANIDAE Kirby, 1837**

**Subfamily BRONTINAE Blanchard, 1845**

**Tribe Brontini Blanchard, 1845**

*Dendrophagus crenatus* (Paykull, 1799)

*Uleiota planatus* (Linnaeus, 1760)

**Tribe Telephanini LeConte, 1861**

*Psammoecus bipunctatus* (Fabricius, 1792)

**Family SILVANINAE Kirby, 1837**

*Silvanoprus fagi* (Guérin-Méneville, 1844)

*Silvanus bidentatus* (Fabricius, 1792)

*Silvanus unidentatus* (G.-A. Olivier, 1790)

**Family CUCUJIDAE Latreille, 1802**

*Cucujus cinnaberinus* (Scopoli, 1763)

*Cucujus haematodes* (Erichson, 1845)

*Pediacus depressus* (Herbst, 1797)

? *Pediacus fuscus* Erichson, 1845 {Plavilshchikov 1964}

**Family PHALACRIDAE Leach, 1815**

**Subfamily PHALACRINAE Leach, 1815**

*Olibrus bimaculatus* Küster, 1848

*Phalacrus caricis* Sturm, 1807

*Phalacrus corruscus* (Panzer, 1797)

*Phalacrus substriatus* Gyllenhal, 1813

**Family LAEMOPHLOEIDAE Ganglbauer, 1899**

**Subfamily LAEMOPHLOEINAE Ganglbauer, 1899**

*Cryptolestes corticinus* (Erichson, 1846) {personal collection of S.K. Alekseev, Kaluga}

*Laemophloeus monilis* (Fabricius, 1787)*

*Laemophloeus muticus* (Fabricius, 1781)

*Lathropus sepicola* (P.W.J. Müller, 1821)

*Leptophloeus alternans* (Erichson, 1846)

*Placonotus testaceus* (Fabricius, 1787)

**Superfamily COCCINELLOIDEA Latreille, 1807**

**Family BOTHRIDERIDAE Erichson, 1845**

*Bothrideres bipunctatus* (Gmelin, 1790)

**Family CERYLONIDAE Billberg, 1820**

**Subamily CERYLONINAE Billberg, 1820**

*Cerylon deplanatum* Gyllenhal, 1827

*Cerylon fagi* C.N.F. Brisout de Barneville, 1867

*Cerylon ferrugineum* Stephens, 1830

*Cerylon histeroides* (Fabricius, 1792)

*Cerylon impressum* Erichson, 1845

**Family LATRIDIIDAE Erichson, 1842**

**Subfamily LATRIDIINAE Erichson, 1842**

**Tribe Latridiini Erichson, 1842**

Cartodere (Cartodere) constricta (Gyllenhal, 1827)

*Enicmus brevicornis* (Mannerheim, 1844)

*Enicmus histrio* Joy & Tomlin, 1910

*Enicmus rugosus* (Herbst, 1793)

*Enicmus testaceus* (Stephens, 1830)

*Enicmus transversus* (G.-A. Olivier, 1790)

*Latridius brevicollis* (C.G. Thomson, 1868)

*Latridius consimilis* (Mannerheim, 1844)

*Latridius hirtus* Gyllenhal, 1827

*Latridius minutus* (Linnaeus, 1767)†

*Latridius porcatus* Herbst, 1793

*Stephostethus angusticollis* (Gyllenhal, 1827)

*Stephostethus lardarius* (De Geer, 1775)

*Stephostethus pandellei* (C.N.F. Brisout de Barneville, 1863)

*Thes bergrothi* (Reitter, 1881)

**Subfamily CORTICARIINAE Curtis, 1829**

*Corticaria ferruginea* Marsham, 1802†

*Corticarina minuta* (Fabricius, 1792)

*Corticarina truncatella* (Mannerheim, 1844)

*Cortinicara gibbosa* (Herbst, 1793)

Melanophthalma (Melanophthalma) transversalis (Gyllenhal, 1827)

**Family CORYLOPHIDAE LeConte, 1852**

**Subfamily CORYLOPHINAE LeConte, 1852**

**Tribe Corylophini LeConte, 1852**

*Corylophus cassidoides* (Marsham, 1802)

**Tribe Parmulini Poey, 1854**

*Arthrolips convexiuscula* (Motschulsky, 1849)

*Clypastraea pusilla* (Gyllenhal, 1810)

**Tribe Sericoderini Matthews, 1886**

*Sericoderus lateralis* (Gyllenhal, 1827)

**Family ANAMORPHIDAE Strohecker, 1953**

*Clemmus troglodytes* Hampe, 1850 {collection of Museum & Institute of Zoology, Polish Academy of Sciences, Warszawa, Poland}

**Family ENDOMYCHIDAE Leach, 1815**

**Subfamily ENDOMYCHINAE Leach, 1815**

*Endomychus coccineus* (Linnaeus, 1758)

**Subfamily LEIESTINAE C.G. Thomson, 1863**

*Leiestes seminiger* (Gyllenhal, 1808)

**Subfamily LYCOPERDININAE Bromhead, 1838**

*Dapsa horvathi* (Csiki, 1901)

*Lycoperdina succincta* (Linnaeus, 1767) {personal collection of S.K. Alekseev, Kaluga}

*Mycetina cruciata* (Schaller, 1783)

**Family COCCINELLIDAE Latreille, 1807**

**Subfamily COCCIDULINAE Mulsant, 1846**

**Tribe Coccidulini Mulsant, 1846**

*Coccidula rufa* (Herbst, 1783)

**Subfamily SCYMNINAE Mulsant, 1846**

**Tribe Hyperaspidini Mulsant, 1846**

Hyperaspis (Hyperaspis) concolor (Suffrian, 1843)

Hyperaspis (Hyperaspis) reppensis (Herbst, 1783)

**Tribe Scymnini Mulsant, 1846**

Nephus (Bipunctatus) bipunctatus (Kugelann, 1794)

Nephus (Nephus) redtenbacheri (Mulsant, 1846)

Scymnus (Neopullus) haemorrhoidalis Herbst, 1797

Scymnus (Pullus) ferrugatus (Moll, 1785)

Scymnus (Pullus) suturalis Thunberg, 1795

Scymnus (Scymnus) frontalis (Fabricius, 1787)

Scymnus (Scymnus) nigrinus Kugelann, 1794

**Tribe Stethorini Dobzhansky, 1924**

Stethorus (Stethorus) pusillus (Herbst, 1797)*

**Subfamily CHILOCORINAE Mulsant, 1846**

**Tribe Chilocorini Mulsant, 1846**

*Chilocorus bipustulatus* (Linnaeus, 1758)

*Chilocorus renipustulatus* (L.G. Scriba, 1791)

*Exochomus quadripustulatus* (Linnaeus, 1758)

**Tribe Platynaspini Mulsant, 1846**

*Platynaspis luteorubra* (Goeze, 1777)

**Subfamily COCCINELLINAE Latreille, 1807**

**Tribe Halyziini Mulsant, 1846**

*Halyzia sedecimguttata* (Linnaeus, 1758)

Psyllobora (Thea) vigintiduopunctata (Linnaeus, 1758)

*Vibidia duodecimguttata* (Poda von Neuhaus, 1761)

**Tribe Tytthaspidini Crotch, 1874**

*Anisosticta novemdecimpunctata* (Linnaeus, 1758)

*Coccinula quatuordecimpustulata* (Linnaeus, 1758)

*Tytthaspis gebleri* (Mulsant, 1850)

*Tytthaspis sedecimpunctata* (Linnaeus, 1760)

**Tribe Coccinellini Latreille, 1807**

Adalia (Adalia) bipunctata (Linnaeus, 1758)

Adalia (Adalia) decempunctata (Linnaeus, 1758)

*Anatis ocellata* (Linnaeus, 1758)

*Calvia decemguttata* (Linnaeus, 1767) {ZIN}

*Calvia quatuordecimguttata* (Linnaeus, 1758)

*Calvia quindecimguttata* (Fabricius, 1777)

Ceratomegilla (Ceratomegilla) notata (Laicharting, 1781)

Coccinella (Coccinella) hieroglyphica hieroglyphica Linnaeus, 1758

Coccinella (Coccinella) magnifica L. Redtenbacher, 1843

Coccinella (Coccinella) quinquepunctata Linnaeus, 1758

Coccinella (Coccinella) septempunctata Linnaeus, 1758

*Harmonia axyridis* (Pallas, 1773)†

*Harmonia quadripunctata* (Pontoppidan, 1763)

Hippodamia (Hemisphaerica) tredecimpunctata (Linnaeus, 1758)

Hippodamia (Hippodamia) variegata (Goeze, 1777)

Myrrha (Myrrha) octodecimguttata (Linnaeus, 1758)

*Mysia oblongoguttata oblongoguttata* (Linnaeus, 1758)

*Oenopia conglobata conglobata* (Linnaeus, 1758)

*Propylea quatuordecimpunctata* (Linnaeus, 1758)

*Sospita vigintiguttata* (Linnaeus, 1758)

**Subfamily EPILACHNINAE Mulsant, 1846**

**Tribe Epilachnini Mulsant, 1846**

*Subcoccinella vigintiquatuorpunctata* (Linnaeus, 1758)

**Superfamily TENEBRIONOIDEA Latreille, 1802**

**Family MYCETOPHAGIDAE Leach, 1815**

**Subfamily MYCETOPHAGINAE Leach, 1815**

**Tribe Mycetophagini Leach, 1815**

Litargus (Litargus) connexus (Geoffroy, 1785)

Mycetophagus (Arnoldiellus) tschitscherini (Reitter, 1897)

Mycetophagus (Ilendus) multipunctatus Fabricius, 1792

Mycetophagus (Mycetophagus) ater (Reitter, 1879)

Mycetophagus (Mycetophagus) quadripustulatus (Linnaeus, 1760)

Mycetophagus (Mycetoxides) fulvicollis Fabricius, 1792

Mycetophagus (Philomyces) populi Fabricius, 1798

Mycetophagus (Ulolendus) atomarius (Fabricius, 1787)

Mycetophagus (Ulolendus) piceus (Fabricius, 1777)

*Triphyllus bicolor* (Fabricius, 1777)

**Tribe Typhaeini C.G. Thomson, 1863**

*Typhaea stercorea* (Linnaeus, 1758)†

**Family CIIDAE Leach, 1819**

**Subfamily CIINAE Leach, 1819**

*Cis bidentatus* (G.-A. Olivier, 1790)

*Cis boleti* (Scopoli, 1763)

*Cis castaneus* (Herbst, 1793)

*Cis jacquemartii* Mellié, 1848

*Cis micans* (Fabricius, 1792)

*Ennearthron cornutum* (Gyllenhal, 1827)

*Hadraule elongatula* (Gyllenhal, 1827)

*Octotemnus glabriculus* (Gyllenhal, 1827)

*Orthocis alni* (Gyllenhal, 1813)

*Orthocis lucasi* (Abeille de Perrin, 1874)

*Sulcacis nitidus* (Fabricius, 1792)

**Family TETRATOMIDAE Billberg, 1820**

**Subfamily TETRATOMINAE Billberg, 1820**

Tetratoma (Abstrulia) ancora Fabricius, 1790* {ZIN}

**Subfamily HALLOMENINAE Gistel, 1848**

Hallomenus (Hallomenus) axillaris (Illiger, 1807)

Hallomenus (Hallomenus) binotatus (Quensel, 1790)

**Family MELANDRYIDAE Leach, 1815**

**Subfamily MELANDRYINAE Leach, 1815**

**Tribe Dircaeini Kirby, 1837**

Abdera (Caridua) affinis (Paykull, 1799)

Abdera (Caridua) flexuosa (Paykull, 1799)

*Dircaea quadriguttata* (Paykull, 1798)

Phloiotrya (Phloiotrya) subtilis (Reitter, 1897)

*Wanachia triguttata* (Gyllenhal, 1810)

**Tribe Hypulini Gistel, 1848**

*Hypulus quercinus* (Quensel, 1790)

**Tribe Melandryini Leach, 1815**

Melandrya (Melandrya) barbata (Fabricius, 1787) {ZIN}

Melandrya (Paramelandrya) dubia (Schaller, 1783)

Phryganophilus (Phryganophilus) pseudauritus Nikitsky, 1988* {ZIN}

Phryganophilus (Phryganophilus) ruficollis (Fabricius, 1798)

**Tribe Orchesiini Mulsant, 1856**

Orchesia (Clinocara) fasciata (Illiger, 1798)

Orchesia (Orchesia) micans (Panzer, 1793)

**Tribe Serropalpini Latreille, 1829**

*Serropalpus barbatus* (Schaller, 1783)

**Tribe Xylitini C.G. Thomson, 1864**

*Xylita laevigata* (Hellenius, 1786)

**Tribe Zilorini Desbrochers des Loges, 1900**

*Zilora elongata* J.R. Sahlberg, 1881

**Subfamily OSPHYINAE Mulsant, 1856 (1839)**

*Osphya bipunctata* (Fabricius, 1775)

**Family RIPIPHORIDAE Laporte, 1840**

**Subfamily PELECOTOMINAE Guérin-Méneville, 1857**

*Pelecotoma fennica* (Paykull, 1799)

**Subfamily RIPIPHORINAE Laporte, 1840**

**Tribe Ripihorini Laporte, 1840**

*Metoecus paradoxus* (Linnaeus, 1760)

**Family ZOPHERIDAE Solier, 1834**

**Subfamily COLYDIINAE Billberg, 1820**

**Tribe Colydiini Billberg, 1820**

*Aulonium trisulcum* (Geoffroy, 1785)

*Colydium filiforme* Fabricius, 1792

**Tribe Synchitini L. Redtenbacher, 1845**

*Bitoma crenata* (Fabricius, 1775)

*Synchita humeralis* (Fabricius, 1792)

**Family MORDELLIDAE Latreille, 1802**

**Subfamily MORDELLINAE Latreille, 1802**

**Tribe Curtimordini Odnosum, 2010**

*Curtimorda maculosa* (Næzén, 1794)

**Tribe Mordellini Latreille, 1802**

*Hoshihananomia perlata* (Sulzer, 1776)

*Mordella aculeata* Linnaeus, 1758

*Mordella brachyura brachyura* Mulsant, 1856

*Mordella holomelaena holomelaena* Apfelbeck, 1914

*Mordella viridescens* A. Costa, 1854

*Mordellaria aurofasciata* (Comolli, 1837)

*Tomoxia bucephala bucephala* A. Costa, 1854

Variimorda (Variimorda) briantea (Comolli, 1837)

Variimorda (Variimorda) mendax Méquignon, 1946

Variimorda (Variimorda) villosa (Schrank von Paula, 1781)

**Tribe Mordellistenini Ermisch, 1941**

Mordellistena (Mordellistena) hirtipes Schilsky, 1895

Mordellistena (Mordellistena) humeralis (Linnaeus, 1758)

Mordellistena (Mordellistena) micans (Germar, 1817)

Mordellistena (Mordellistena) parvicauda Ermisch, 1967

Mordellistena (Mordellistena) pentas Mulsant, 1856

Mordellistena (Mordellistena) pumila (Gyllenhal, 1810)

Mordellistena (Mordellistena) rugipennis Schilsky, 1895

Mordellistena (Mordellistena) secreta Horák, 1983

Mordellistena (Mordellistena) stenidea Mulsant, 1856

Mordellistena (Mordellistena) thuringiaca Ermisch, 1963

Mordellistena (Mordellistena) variegata (Fabricius, 1798)

**Tribe Mordellochroini Odnosum, 2010**

*Mordellochroa abdominalis* (Fabricius, 1775)

**Family TENEBRIONIDAE Latreille, 1802**

**Subfamily LAGRIINAE Latreille, 1825 (1820)**

**Tribe Lagriini Latreille, 1825 (1820)**

Subtribe Lagriina Latreille, 1825 (1820)

Lagria (Lagria) hirta (Linnaeus, 1758)

Lagria (Lagria) laticollis Motschulsky, 1860

**Subfamily TENEBRIONINAE Latreille, 1802**

**Tribe Alphitobiini Reitter, 1917**

*Diaclina fagi* (Panzer, 1799) {ZIN}

**Tribe Bolitophagini Kirby, 1837**

Subtribe Bolitophagina Kirby, 1837

*Bolitophagus reticulatus* (Linnaeus, 1767)

*Eledona agricola* (Herbst, 1783)

**Tribe Melanimonini Seidlitz, 1894 (1854)**

*Melanimon tibialis tibialis* (Fabricius, 1781)

**Tribe Opatrini Brullé, 1832**

Subtribe Opatrina Brullé, 1832

Opatrum (Opatrum) riparium W. Scriba, 1865

Opatrum (Opatrum) sabulosum sabulosum (Linnaeus, 1760)

**Tribe Palorini Matthews, 2003**

*Palorus depressus* (Fabricius, 1790)

**Tribe Pedinini Eschscholtz, 1829**

Subtribe Pedinina Eschscholtz, 1829

Pedinus (Pedinus) femoralis femoralis (Linnaeus, 1767)

**Tribe Tenebrionini Latreille, 1802**

*Bius thoracicus* (Fabricius, 1792)

*Neatus picipes* (Herbst, 1797)

*Tenebrio molitor* Linnaeus, 1758†

**Tribe Triboliini Gistel, 1848**

*Tribolium confusum* Jacquelin du Val, 1861†

*Tribolium destructor* Uyttenboogaart, 1933†

**Tribe Ulomini Blanchard, 1845**

Uloma (Uloma) culinaris (Linnaeus, 1758)

Uloma (Uloma) rufa (Piller & Mitterpacher, 1783)

**Subfamily DIAPERINAE Latreille, 1802**

**Tribe Crypticini Brullé, 1832**

Crypticus (Crypticus) quisquilius quisquilius (Linnaeus, 1760)

**Tribe Diaperini Latreille, 1802**

*Diaperis boleti boleti* (Linnaeus, 1758)

*Neomida haemorrhoidalis* (Fabricius, 1787)

*Platydema dejeanii* Laporte & Brullé, 1831

**Tribe Hypophlaeini Billberg, 1820**

Corticeus (Corticeus) bicolor (G.-A. Olivier, 1790)

Corticeus (Corticeus) fasciatus (Fabricius, 1790)

Corticeus (Corticeus) fraxini (Kugelann, 1794)

Corticeus (Corticeus) linearis (Fabricius, 1790)

Corticeus (Corticeus) longulus (Gyllenhal, 1827)

Corticeus (Corticeus) pini (Panzer, 1799)

Corticeus (Corticeus) suturalis (Paykull, 1800)

Corticeus (Corticeus) unicolor Piller & Mitterpacher, 1783

**Tribe Scaphidemini Reitter, 1922**

*Scaphidema metallica metallica* (Fabricius, 1792)

**Subfamily ALLECULINAE Laporte, 1840**

**Tribe Alleculini Laporte, 1840**

Subtribe Alleculina Laporte, 1840

*Hymenorus doublieri* Mulsant, 1852

Subtribe Gonoderina Seidlitz, 1896

Isomira (Isomira) murina murina (Linnaeus, 1758)

*Pseudocistela ceramboides* (Linnaeus, 1758)

Subtribe Mycetocharina Gistel, 1848

Mycetochara (Mycetochara) axillaris (Paykull, 1799)

Mycetochara (Mycetochara) flavipes (Fabricius, 1792)

**Tribe Cteniopodini Solier, 1835**

? Cteniopus (Cteniopus) sulphureus (Linnaeus, 1758)

(indicated as *C. flavus* (Scopoli, 1763) {Plavilshchikov 1964})

**Subfamily STENOCHIINAE Kirby, 1837**

**Tribe Cnodalonini Oken, 1843**

*Upis ceramboides* (Linnaeus, 1758)

**Family OEDEMERIDAE Latreille, 1810**

**Subfamily CALOPODINAE Costa, 1852**

*Calopus serraticornis* (Linnaeus, 1758)

**Subfamily OEDEMERINAE Latreille, 1810**

**Tribe Ditylini Mulsant, 1858**

*Chrysanthia geniculata geniculata* W.L.E. Schmidt, 1846

*Chrysanthia viridissima* (Linnaeus, 1758)

*Ditylus laevis laevis* (Fabricius, 1787)

**Tribe Oedemerini Latreille, 1810**

Oedemera (Oedemera) femorata (Scopoli, 1763)

Oedemera (Oedemera) lurida lurida (Marsham, 1802)

Oedemera (Oedemera) virescens virescens (Linnaeus, 1767)

**Family MELOIDAE Gyllenhal, 1810**

**Subfamily MELOINAE Gyllenhal, 1810**

**Tribe Cerocomini Leach, 1815**

Cerocoma (Cerocoma) schaefferi (Linnaeus, 1758)

**Tribe Lyttini Streubel, 1846**

*Alosimus syriacus austriacus* (Schrank von Paula, 1781)

Lytta (Lytta) vesicatoria vesicatoria (Linnaeus, 1758)

**Tribe Mylabrini Rafinesque, 1815**

? *Hycleus polymorphus polymorphus* (Pallas, 1771) {Plavilshchikov 1964}

? Mylabris (Eumylabris) fabricii Sumakov, 1924 {Plavilshchikov 1964}

Mylabris (Micrabris) sibirica Fischer von Waldheim, 1823

**Tribe Meloini Gyllenhal, 1810**

Meloe (Eurymeloe) brevicollis brevicollis Panzer, 1793

Meloe (Lampromeloe) variegatus variegatus Donovan, 1793

Meloe (Meloe) proscarabaeus proscarabaeus Linnaeus, 1758

Meloe (Meloe) violaceus Marsham, 1802

**Family BORIDAE C.G. Thomson, 1859**

**Subfamily BORINAE C.G. Thomson, 1859**

*Boros schneideri* (Panzer, 1796)

**Family PYTHIDAE Solier, 1834**

*Pytho depressus* (Linnaeus, 1767)

**Family PYROCHROIDAE Latreille, 1806**

**Subfamily PYROCHROINAE Latreille, 1806**

*Pyrochroa coccinea* (Linnaeus, 1760)

*Schizotus pectinicornis* (Linnaeus, 1758)

**Family SALPINGIDAE Leach, 1815**

**Subfamily SALPINGINAE Leach, 1815**

*Lissodema cursor* (Gyllenhal, 1813)

*Rabocerus foveolatus* (Ljungh, 1823)

*Salpingus planirostris* (Fabricius, 1787)

*Salpingus ruficollis* (Linnaeus, 1760)

*Sphaeriestes bimaculatus* (Gyllenhal, 1810)

**Family ANTHICIDAE Latreille, 1819**

**Subfamily ANTHICINAE Latreille, 1819**

**Tribe Anthicini Latreille, 1819**

*Anthicus antherinus antherinus* (Linnaeus, 1760)

*Anthicus ater* (Thunberg, 1787)

*Hirticomus hispidus* (Rossi, 1792)

*Omonadus floralis* (Linnaeus, 1758)†

**Subfamily NOTOXINAE Stephens, 1829**

*Notoxus monoceros* (Linnaeus, 1760)

**Family ADERIDAE Csiki, 1909**

**Tribe Aderini Csiki, 1909**

*Aderus populneus* (Creutzer, 1796)

*Anidorus nigrinus* (Germar, 1842)

**Tribe Euglenesini Seidlitz, 1875**

*Euglenes pygmaeus* (De Geer, 1775)

**Tribe Phytobaenini Báguena Corella, 1948**

*Phytobaenus amabilis amabilis* R.F. Sahlberg, 1834

**Family SCRAPTIIDAE Gistel, 1848**

**Subfamily SCRAPTIINAE Gistel, 1848**

**Tribe Scraptiini Gistel, 1848**

*Scraptia fuscula* P.W.J. Müller, 1821

**Subfamily ANASPIDINAE Mulsant, 1856**

**Tribe Anaspidini Mulsant, 1856**

Anaspis (Anaspis) frontalis (Linnaeus, 1758)

Anaspis (Anaspis) thoracica (Linnaeus, 1758)

Anaspis (Nassipa) rufilabris (Gyllenhal, 1827)

*Cyrtanaspis phalerata* (Germar, 1847)

**Superfamily CHRYSOMELOIDEA Latreille, 1802**

**Family CERAMBYCIDAE Latreille, 1802**

**Subfamily PRIONINAE Latreille, 1802**

**Tribe Prionini Latreille, 1802**

*Prionus coriarius* (Linnaeus, 1758)

**Subfamily LEPTURINAE Latreille, 1802**

**Tribe Lepturini Latreille, 1802**

*Alosterna ingrica* (Baeckmann, 1902)

*Alosterna tabacicolor tabacicolor* (De Geer, 1775)

*Anastrangalia reyi* (L. Heyden, 1889)

*Anastrangalia sanguinolenta* (Linnaeus, 1760)

Anoplodera (Anoplodera) sexguttata (Fabricius, 1775)

Etorofus (Etorofus) pubescens (Fabricius, 1787)

*Judolia sexmaculata* (Linnaeus, 1758)

Leptura (Leptura) annularis annularis Fabricius, 1801

Leptura (Leptura) aurulenta Fabricius, 1793 {ZIN}

Leptura (Leptura) quadrifasciata quadrifasciata Linnaeus, 1758

Leptura (Macroleptura) thoracica Creutzer, 1799

*Lepturalia nigripes nigripes* (De Geer, 1775)

*Lepturobosca virens* (Linnaeus, 1758)

*Nivellia sanguinosa* (Gyllenhal, 1827)

*Oedecnema gebleri* (Ganglbauer, 1889)

*Pseudovadonia livida bicarinata* (N. Arnold, 1869)

*Rutpela maculata maculata* (Poda von Neuhaus, 1761)

Stenurella (Priscostenurella) bifasciata bifasciata (O.F. Müller, 1776)

Stenurella (Stenurella) melanura melanura (Linnaeus, 1758)

Stictoleptura (Aredolpona) rubra rubra (Linnaeus, 1758)

Stictoleptura (Maculileptura) maculicornis (De Geer, 1775)

Stictoleptura (Variileptura) variicornis (Dalman, 1817)

*Strangalia attenuata* (Linnaeus, 1758)

**Tribe Oxymirini Danilevsky, 1997**

*Oxymirus cursor* (Linnaeus, 1758)

**Tribe Rhagiini Kirby, 1837**

Brachyta (Brachyta) interrogationis russica (Herbst, 1784)

*Carilia virginea virginea* (Linnaeus, 1758)

*Cortodera femorata* (Fabricius, 1787)

*Dinoptera collaris* (Linnaeus, 1758)

*Euracmaeops angusticollis* (Gebler, 1833)

*Euracmaeops marginatus* (Fabricius, 1781)

*Euracmaeops septentrionis* (C.G. Thomson, 1866)

Evodinellus (Evodinellus) borealis (Gyllenhal, 1827)

*Gnathacmaeops pratensis* (Laicharting, 1784)

*Pachyta quadrimaculata* (Linnaeus, 1758)

Rhagium (Megarhagium) mordax (De Geer, 1775)

Rhagium (Megarhagium) sycophanta (Schrank, 1781)

Rhagium (Rhagium) inquisitor inquisitor (Linnaeus, 1758)

Stenocorus (Stenocorus) meridianus (Linnaeus, 1758)

**Tribe Rhamnusiini Sama, 2009**

*Rhamnusium bicolor constans* Danilevsky, 2012

**Subfamily NECYDALINAE Latreille, 1825**

Necydalis (Necydalis) major Linnaeus, 1758

**Subfamily SPONDYLIDINAE Audinet-Serville, 1832**

**Tribe Asemini J. Thomson, 1861**

*Arhopalus rusticus rusticus* (Linnaeus, 1758)

*Asemum striatum* (Linnaeus, 1758)

**Tribe Spondylidini Audinet-Serville, 1832**

*Spondylis buprestoides* (Linnaeus, 1758)

**Tribe Tetropiini Seidlitz, 1891**

*Tetropium castaneum* (Linnaeus, 1758)

*Tetropium fuscum fuscum* (Fabricius, 1787) {personal collection of S.K. Alekseev, Kaluga}

**Subfamily CERAMBYCINAE Latreille, 1802**

**Tribe Callichromatini Swainson & Shuckard, 1840**

*Aromia moschata moschata* (Linnaeus, 1758)

**Tribe Callidiini Kirby, 1837**

Callidium (Callidium) violaceum (Linnaeus, 1758)

Callidium (Callidostola) aeneum aeneum (De Geer, 1775)

Callidium (Palaeocallidium) coriaceum (Paykull, 1800)

Phymatodes (Phymatoderus) abietinus Plavilstshikov & Lurie, 1960 {ZIN}

*Semanotus undatus* (Linnaeus, 1758)

**Tribe Clytini Mulsant, 1839**

Chlorophorus (Immaculatus) herbstii (Brahm, 1790)

Clytus (Clytus) arietis arietis (Linnaeus, 1758)

*Cyrtoclytus capra* (Germar, 1823)

*Plagionotus arcuatus arcuatus* (Linnaeus, 1758)

*Plagionotus detritus detritus* (Linnaeus, 1758)

*Rhaphuma gracilipes* (Faldermann, 1835)

Xylotrechus (Rusticoclytus) rusticus (Linnaeus, 1758)

Xylotrechus (Xylotrechus) antilope antilope (Schoenherr, 1817)

Xylotrechus (Xylotrechus) capricornus (Gebler, 1830)

**Tribe Deilini Fairmaire, 1864**

*Deilus fugax* (G.-A. Olivier, 1790)

**Tribe Hesperophanini Mulsant, 1839**

Subtribe Hesperophanina Mulsant, 1839

*Trichoferus campestris* (Faldermann, 1835)†

**Tribe Molorchini Gistel, 1848**

Molorchus (Caenoptera) minor minor (Linnaeus, 1758)

Molorchus (Molorchus) marmottani marmottani Brisout de Barneville, 1863

**Tribe Obriini Mulsant, 1839**

*Obrium cantharinum cantharinum* (Linnaeus, 1767)

**Tribe Purpuricenini J. Thomson, 1861**

*Purpuricenus globulicollis globulicollis* Dejean, 1839 {ZIN}

*Purpuricenus kaehleri kaehleri* (Linnaeus, 1758)

**Subfamily LAMIINAE Latreille, 1825**

**Tribe Acanthocinini Blanchard, 1845**

Acanthocinus (Acanthocinus) aedilis (Linnaeus, 1758)

Acanthocinus (Acanthocinus) griseus (Fabricius, 1793)

Leiopus (Leiopus) linnei Wallin, Nylander & Kvamme, 2009

**Tribe Acanthoderini J. Thomson, 1860**

*Aegomorphus clavipes* (Schrank, 1781)

*Aegomorphus obscurior* (Pic, 1904)

*Oplosia cinerea* (Mulsant, 1839)*

**Tribe Agapanthiini Mulsant, 1839**

Agapanthia (Agapanthia) cardui (Linnaeus, 1767)

Agapanthia (Epoptes) villosoviridescens (De Geer, 1775)

Agapanthia (Smaragdula) intermedia Ganglbauer, 1884

**Tribe Exocentrini Pascoe, 1864**

*Exocentrus lusitanus* (Linnaeus, 1767)

**Tribe Lamiini Latreille, 1825**

*Lamia textor* (Linnaeus, 1758)

**Tribe Mesosini Mulsant, 1839**

Mesosa (Mesosa) myops (Dalman, 1817)

**Tribe Monochamini Gistel, 1848**

Monochamus (Monochamus) galloprovincialis pistor (Germar, 1818)

Monochamus (Monochamus) saltuarius occidentalis Sláma, 2017

Monochamus (Monochamus) sutor sutor (Linnaeus, 1758)

Monochamus (Monochamus) urussovii (Fischer von Waldheim, 1805)

**Tribe Phytoeciini Mulsant, 1839**

Oberea (Oberea) oculata (Linnaeus, 1758)

Phytoecia (Opsilia) coerulescens coerulescens (Scopoli, 1763)

Phytoecia (Phytoecia) cylindrica (Linnaeus, 1758)

Phytoecia (Phytoecia) nigricornis (Fabricius, 1782)

Phytoecia (Phytoecia) pustulata pustulata (Schrank, 1776)

**Tribe Pogonocherini Mulsant, 1839**

Pogonocherus (Pogonocherus) hispidulus (Piller & Mitterpacher, 1783)

Pogonocherus (Pityphilus) decoratus Fairmaire, 1855

Pogonocherus (Pityphilus) fasciculatus fasciculatus (De Geer, 1775)

**Tribe Saperdini Mulsant, 1839**

Saperda (Lopezcolonia) perforata (Pallas, 1773)

Saperda (Lopezcolonia) scalaris scalaris (Linnaeus, 1758)

Saperda (Saperda) carcharias (Linnaeus, 1758)

*Stenostola dubia* (Laicharting, 1784) {ZIN}

*Stenostola ferrea ferrea* (Schrank, 1776)

**Tribe Tetropini Portevin, 1927**

Tetrops (Tetrops) praeustus praeustus (Linnaeus, 1758)

**Family MEGALOPODIDAE Latreille, 1802**

**Subfamily ZEUGOPHORINAE Böving & Craighead, 1931**

Zeugophora (Zeugophora) scutellaris Suffrian, 1840

Zeugophora (Zeugophora) subspinosa (Fabricius, 1781) {ZIN}

**Family ORSODACNIDAE C.G. Thomson, 1859**

**Subfamily ORSODACNINAE C.G. Thomson, 1859**

*Orsodacne cerasi* (Linnaeus, 1758)

**Family CHRYSOMELIDAE Latreille, 1802**

**Subfamily BRUCHINAE Latreille, 1802**

**Tribe Amblycerini Bridwell, 1932**

Subtribe Spermophagina Borowiec, 1987

*Spermophagus sericeus* (Geoffroy, 1785)

**Tribe Bruchini Latreille, 1802**

Subtribe Bruchina Latreille, 1802

*Bruchus atomarius* (Linnaeus, 1760)

*Bruchus loti* Paykull 1800

**Subfamily DONACIINAE Kirby, 1837**

**Tribe Donaciini Kirby, 1837**

*Donacia antiqua* Kunze, 1818

*Donacia aquatica* (Linnaeus, 1758)

*Donacia bicolora bicolora* Zschach, 1788

*Donacia cinerea* Herbst, 1784

*Donacia clavipes clavipes* Fabricius, 1792

*Donacia crassipes* Fabricius, 1775

*Donacia dentata* Hoppe, 1795

*Donacia impressa* Paykull, 1799

*Donacia marginata* Hoppe, 1795

*Donacia obscura* Gyllenhal, 1813

*Donacia semicuprea* Panzer, 1796

*Donacia tomentosa* Ahrens, 1810

*Donacia vulgaris vulgaris* Zschach, 1788

**Tribe Plateumarini Boving, 1922**

Plateumaris (Euplateumaris) discolor discolor (Panzer, 1795)

Plateumaris (Euplateumaris) sericea sericea (Linnaeus, 1758)

**Subfamily CRIOCERINAE Latreille, 1804**

? *Crioceris asparagi* (Linnaeus, 1758) {Plavilshchikov 1964}

Lema (Lema) cyanella (Linnaeus, 1758)

*Lilioceris merdigera* (Linnaeus, 1758)

*Oulema erichsonii* (Suffrian, 1841)

*Oulema gallaeciana* (L. Heyden, 1870)

*Oulema melanopus* (Linnaeus, 1758)

**Subfamily CASSIDINAE Gyllenhal, 1813**

**Tribe Cassidini Gyllenhal, 1813**

*Cassida denticollis* Suffrian, 1844

*Cassida flaveola* Thunberg, 1794

*Cassida lineola* Creutzer, 1799

*Cassida margaritacea* Schaller, 1783

*Cassida nebulosa* Linnaeus, 1758

*Cassida nobilis* Linnaeus, 1758

*Cassida pannonica* Suffrian, 1844

*Cassida panzeri* J. Weise, 1907

*Cassida prasina* Illiger, 1798

*Cassida rubiginosa rubiginosa* O.F. Müller, 1776

*Cassida sanguinolenta* O.F. Müller, 1776

*Cassida sanguinosa* Suffrian, 1844

*Cassida stigmatica* Suffrian, 1844

*Cassida subreticulata* Suffrian, 1844

*Cassida vibex* Linnaeus, 1767

*Cassida viridis* Linnaeus, 1758

*Hypocassida subferruginea* (Schrank, 1776)

**Tribe Hispini Gyllenhal, 1813**

*Hispa atra* Linnaeus, 1767

**Subfamily CHRYSOMELINAE Latreille, 1802**

**Tribe Chrysomelini Latreille, 1802**

Subtribe Chrysomelina Latreille, 1802

*Chrysomela collaris* Linnaeus, 1758

*Chrysomela populi* Linnaeus, 1758

*Chrysomela tremula* Fabricius, 1787

*Chrysomela vigintipunctata* (Scopoli, 1763)

*Plagiodera versicolora* (Laicharting, 1781)

*Plagiosterna aenea* (Linnaeus, 1758)

Subtribe Gastrophysina Kippenberg, 2010

Gastrophysa (Gastrophysa) polygoni polygoni (Linnaeus, 1758)

Gastrophysa (Gastrophysa) viridula viridula (De Geer, 1775)

Subtribe Phratorina Motschulsky, 1860

Phratora (Phratora) vulgatissima (Linnaeus, 1758)

Phratora (Phyllodecta) atrovirens (Cornelius, 1857)

Phratora (Phyllodecta) laticollis (Suffrian, 1851)

Phratora (Phyllodecta) tibialis tibialis (Suffrian, 1851)

Phratora (Phyllodecta) vitellinae (Linnaeus, 1758)

Subtribe Prasocurina Gistel, 1848

Phaedon (Phaedon) armoraciae (Linnaeus, 1758)

Phaedon (Phaedon) cochleariae cochleariae (Fabricius, 1792)

Phaedon (Phaedon) laevigatus laevigatus (Duftschmid, 1825)

Prasocuris (Hydrothassa) glabra (Herbst, 1783)

Prasocuris (Hydrothassa) hannoveriana (Fabricius, 1775)

Prasocuris (Hydrothassa) marginella marginella (Linnaeus, 1758)

Prasocuris (Prasocuris) junci (Brahm, 1790)

Prasocuris (Prasocuris) phellandrii (Linnaeus, 1758)

**Tribe Doryphorini Motschulsky, 1860**

Subtribe Chrysolinina S.-H. Chen, 1936

Chrysolina (Anopachys) eurina (Frivaldszky, 1883)† {ZIN}

Chrysolina (Chalcoidea) analis (Linnaeus, 1767)

Chrysolina (Chalcoidea) besseri (Krynicki, 1832)

Chrysolina (Chalcoidea) marginata marginata (Linnaeus, 1758)

Chrysolina (Chrysolina) staphylaea staphylaea (Linnaeus, 1758)

Chrysolina (Colaphodes) haemoptera (Linnaeus, 1758)

Chrysolina (Colaphosoma) sturmi sturmi (Westhoff, 1882)

Chrysolina (Erythrochrysa) polita polita (Linnaeus, 1758)

Chrysolina (Euchrysolina) graminis graminis (Linnaeus, 1758)

Chrysolina (Fastuolina) fastuosa fastuosa (Scopoli, 1763)

Chrysolina (Hypericia) geminata (Paykull, 1799)

Chrysolina (Hypericia) hyperici (Forster, 1771)

Chrysolina (Sphaeromela) varians (Schaller, 1783)

Chrysolina (Stichoptera) gypsophilae (Küster, 1845)

Chrysolina (Stichoptera) sanguinolenta (Linnaeus, 1758)

Chrysolina (Synerga) herbacea (Duftschmid, 1825)

Chrysolina (Zeugotaenia) limbata russiella Bieńkowski & Orlova-Bienkowskaja, 2011

? *Oreina (Allorina) caerulea* (G.-A. Olivier, 1790) {Plavilshchikov 1964}

Subtribe Doryphorina Motschulsky, 1860

*Leptinotarsa decemlineata* (Say, 1824)†

*Entomoscelis suturalis* J. Weise, 1882

**Tribe Gonioctenini Motschulsky, 1860**

Gonioctena (Gonioctena) decemnotata (Marsham, 1802)

Gonioctena (Gonioctena) linnaeana linnaeana (Schrank, 1781)

Gonioctena (Gonioctena) viminalis viminalis (Linnaeus, 1758)

Gonioctena (Goniomena) pallida (Linnaeus, 1758)

Gonioctena (Goniomena) quinquepunctata quinquepunctata (Fabricius, 1787)

**Subfamily GALERUCINAE Latreille, 1802**

**Tribe Galerucini Latreille, 1802**

Galeruca (Galeruca) jucunda (Faldermann, 1837)

Galeruca (Galeruca) tanaceti tanaceti (Linnaeus, 1758)

Galerucella (Galerucella) grisescens (Joannis, 1866)

Galerucella (Galerucella) nymphaeae (Linnaeus, 1758)

Galerucella (Neogalerucella) calmariensis (Linnaeus, 1767)

Galerucella (Neogalerucella) lineola lineola (Fabricius, 1781)

Galerucella (Neogalerucella) pusilla (Duftschmid, 1825)

Galerucella (Neogalerucella) tenella (Linnaeus, 1760)

*Lochmaea caprea* (Linnaeus, 1758)

*Lochmaea suturalis* (C.G. Thomson, 1866)

*Pyrrhalta viburni* (Paykull, 1799)

**Tribe Hylaspini Chapuis, 1875**

*Agelastica alni* (Linnaeus, 1758)

**Tribe Luperini Gistel, 1848**

Subtribe Luperina Gistel, 1848

*Calomicrus pinicola* (Duftschmid, 1825)

*Luperus luperus* (Sulzer, 1776) {ZIN}

*Phyllobrotica quadrimaculata* (Linnaeus, 1758)

**Subfamily ALTICINAE Newman, 1834**

**Tribe Alticini Newman, 1834**

*Altica palustris* (J. Weise, 1888)

*Altica quercetorum saliceti* J. Weise, 1888

*Altica tamaricis tamaricis* Schrank, 1785

? *Aphthona czwalinae* J. Weise, 1888 {Plavilshchikov 1964}

*Aphthona lutescens* (Gyllenhal, 1813)

*Aphthona nonstriata* (Goeze, 1777)

*Aphthona pallida* (Bach, 1856)

*Argopus nigritarsis* (Gebler, 1823)

*Batophila rubi* (Paykull, 1799)

Chaetocnema (Chaetocnema) aerosa (Letzner, 1847)

Chaetocnema (Chaetocnema) arida Foudras, 1860

Chaetocnema (Chaetocnema) aridula (Gyllenhal, 1827)

Chaetocnema (Chaetocnema) compressa (Letzner, 1847)

Chaetocnema (Chaetocnema) hortensis (Geoffroy, 1785)

Chaetocnema (Chaetocnema) mannerheimii (Gyllenhal, 1827)

Chaetocnema (Tlanoma) concinna (Marsham, 1802)

Chaetocnema (Tlanoma) semicoerulea semicoerulea (Koch, 1803)

*Crepidodera aurata* (Marsham, 1802)

*Crepidodera fulvicornis* (Fabricius, 1792)

*Crepidodera nitidula* (Linnaeus, 1758)

*Crepidodera plutus* (Latreille, 1804)

*Derocrepis rufipes* (Linnaeus, 1758)

*Epitrix pubescens* (Koch, 1803)

*Hippuriphila modeeri* (Linnaeus, 1760)

Longitarsus (Longitarsus) atricillus (Linnaeus, 1760)

Longitarsus (Longitarsus) brunneus (Duftschmid, 1825)

Longitarsus (Longitarsus) ganglbaueri ganglbaueri Heikertinger, 1912

Longitarsus (Longitarsus) holsaticus (Linnaeus, 1758)

Longitarsus (Longitarsus) jacobaeae (C.R. Waterhouse, 1858)

Longitarsus (Longitarsus) longiseta J. Weise, 1889

Longitarsus (Longitarsus) nigrofasciatus nigrofasciatus (Goeze, 1777)

Longitarsus (Longitarsus) succineus (Foudras, 1860)

Longitarsus (Longitarsus) suturellus (Duftschmid, 1825)

Longitarsus (Longitarsus) tabidus tabidus (Fabricius, 1775)

Longitarsus (Testergus) anchusae (Paykull, 1799)

*Lythraria salicariae* (Paykull, 1800)

Mantura (Mantura) chrysanthemi chrysanthemi (Koch, 1803)

*Neocrepidodera ferruginea* (Scopoli, 1763)

*Neocrepidodera transversa* (Marsham, 1802)

*Phyllotreta atra* (Fabricius, 1775)

*Phyllotreta flexuosa* (Illiger, 1794)

*Phyllotreta nemorum* (Linnaeus, 1758)

*Phyllotreta nigripes nigripes* (Fabricius, 1775)

*Phyllotreta ochripes* (Curtis, 1837)

*Phyllotreta striolata* (Fabricius, 1803)

*Phyllotreta tetrastigma* (Comolli, 1837)

*Phyllotreta undulata* Kutschera, 1860

*Phyllotreta vittula* (L. Redtenbacher, 1849)

Psylliodes (Psylliodes) affinis (Paykull, 1799)

Psylliodes (Psylliodes) chalcomera (Illiger, 1807)

Psylliodes (Psylliodes) dulcamarae (Koch, 1803)

Psylliodes (Psylliodes) napi (Fabricius, 1792)

Psylliodes (Psylliodes) picina (Marsham, 1802)

**Subfamily CRYPTOCEPHALINAE Gyllenhal, 1813**

**Tribe Clytrini Kirby, 1837**

Subtribe Clytrina Kirby, 1837

Clytra (Clytra) quadripunctata quadripunctata (Linnaeus, 1758)

*Coptocephala unifasciata unifasciata* (Scopoli, 1763)

Labidostomis (Labidostomis) lepida Lefevre, 1872

Labidostomis (Labidostomis) longimana (Linnaeus, 1760)

Labidostomis (Labidostomis) tridentata (Linnaeus, 1758)

*Smaragdina affinis affinis* (Illiger, 1794)

*Smaragdina flavicollis* (Charpentier, 1825)

**Tribe Cryptocephalini Gyllenhal, 1813**

Subtribe Cryptocephalina Gyllenhal, 1813

Cryptocephalus (Burlinius) exiguus exiguus D.N. Schneider, 1792

Cryptocephalus (Burlinius) fulvus fulvus (Goeze, 1777)

Cryptocephalus (Burlinius) labiatus (Linnaeus, 1760)

Cryptocephalus (Burlinius) pusillus Fabricius, 1777

Cryptocephalus (Cryptocephalus) anticus Suffrian, 1848

Cryptocephalus (Cryptocephalus) aureolus Suffrian, 1847

Cryptocephalus (Cryptocephalus) bipunctatus bipunctatus (Linnaeus, 1758)

Cryptocephalus (Cryptocephalus) biguttatus (Scopoli, 1763)

Cryptocephalus (Cryptocephalus) cordiger (Linnaeus, 1758)

Cryptocephalus (Cryptocephalus) decemmaculatus (Linnaeus, 1758)

Cryptocephalus (Cryptocephalus) flavipes Fabricius, 1781

Cryptocephalus (Cryptocephalus) solivagus Leonardi & Sassi, 2001

Cryptocephalus (Cryptocephalus) laetus Fabricius, 1792

Cryptocephalus (Cryptocephalus) moraei (Linnaeus, 1758)

Cryptocephalus (Cryptocephalus) nitidus (Linnaeus, 1758)

Cryptocephalus (Cryptocephalus) octopunctatus octopunctatus (Scopoli, 1763)

Cryptocephalus (Cryptocephalus) parvulus O.F. Müller, 1776

Cryptocephalus (Cryptocephalus) sericeus (Linnaeus, 1758)

Cryptocephalus (Cryptocephalus) sexpunctatus sexpunctatus (Linnaeus, 1758)

Cryptocephalus (Disopus) pini (Linnaeus, 1758)

Cryptocephalus (Heterichnus) coryli (Linnaeus, 1758)

Subtribe Pachybrachina Chapius, 1784

Pachybrachis (Pachybrachis) hieroglyphicus (Laicharting, 1781)

**Subfamily EUMOLPINAE Hope, 1840**

**Tribe Bromiini Baly, 1865 (1863)**

*Bromius obscurus* (Linnaeus, 1758)

Pachnephorus (Pachnephorus) tessellatus (Duftschmid, 1825)

**Subfamily SYNETINAE LeConte & Horn, 1883**

*Syneta betulae betulae* (Fabricius, 1792)* {ZIN}

**Superfamily CURCULIONOIDEA Latreille, 1802**

**Family NEMONYCHIDAE Bedel, 1882**

**Subfamily CIMBERIDINAE Gozis, 1882**

**Tribe Cimberidini Gozis, 1882**

*Cimberis attelaboides* (Fabricius, 1787)

**Family ANTHRIBIDAE Billberg, 1820**

**Subfamily ANTHRIBINAE Billberg, 1820**

**Tribe Anthribini Billberg, 1820**

*Anthribus nebulosus* Forster, 1770

**Tribe Platyrhinini Imhoff, 1856**

*Platyrhinus resinosus* (Scopoli, 1763)

**Tribe Platystomini Pierce, 1916**

*Platystomos albinus* (Linnaeus, 1758)

**Tribe Tropiderini Lacordaire, 1865**

*Gonotropis dorsalis* (Gyllenhal, 1813)

*Tropideres albirostris* (Schaller, 1783)

**Tribe Zygaenodini Lacordaire, 1865**

*Dissoleucas niveirostris* (Fabricius, 1798)

*Rhaphitropis marchica* (Herbst, 1797)

**Subfamily CHORAGINAE Kirby, 1819**

**Tribe Choragini Kirby, 1819**

*Choragus sheppardi* Kirby, 1819

**Family ATTELABIDAE Billberg, 1820**

**Subfamily ATTELABINAE Billberg, 1820**

**Tribe Apoderini Jekel, 1860**

Subtribe Apoderina Jekel, 1860

*Apoderus coryli* (Linnaeus, 1758)

Compsapoderus (Compsapoderus) erythropterus (Gmelin, 1790)

**Tribe Attelabini Billberg, 1820**

*Attelabus nitens* (Scopoli, 1763)

**Subfamily RHYNCHITINAE Gistel, 1848**

**Tribe Auletini Desbrochers des Loges, 1908**

Subtribe Pseudomesauletina Legalov, 2003

*Mesauletobius pubescens* (Kiesenwetter, 1852)† {ZIN}

**Tribe Byctiscini Voss, 1923**

Subtribe Byctiscina Voss, 1923

*Byctiscus betulae* (Linnaeus, 1758)

*Byctiscus populi* (Linnaeus, 1758)

**Tribe Deporaini Voss, 1929**

Subtribe Deporaina Voss, 1929

Deporaus (Deporaus) betulae (Linnaeus, 1758)

**Tribe Rhynchitini Gistel, 1848**

Involvulus (Involvulus) cupreus (Linnaeus, 1760)

Mecorhis (Pseudomechoris) aethiops (Bach, 1854)

Neocoenorrhinus (Neocoenorrhinus) germanicus (Herbst, 1797)

Rhynchites (Epirhynchites) auratus (Scopoli, 1763)

*Tatianaerhynchites aequatus* (Linnaeus, 1767)

*Temnocerus coeruleus* (Fabricius, 1798)

*Temnocerus nanus* (Paykull, 1792)

**Family BRENTIDAE Billberg, 1820**

**Subfamily APIONINAE Schoenherr, 1823**

**Tribe Apionini Schoenherr, 1823**

Subribe Apionina Schoenherr, 1823

*Apion cruentatum* Walton, 1844

*Apion frumentarium* (Linnaeus, 1758)

*Apion haematodes haematodes* Kirby, 1808

*Apion rubiginosum* Grill, 1893

Subtribe Aplemonina Kissinger, 1968

Perapion (Perapion) connexum (Schilsky, 1902)

Perapion (Perapion) curtirostre (Germar, 1817)

Perapion (Perapion) marchicum (Herbst, 1797)

Perapion (Perapion) oblongum (Gyllenhal, 1839)

Perapion (Perapion) violaceum violaceum (Kirby, 1808)

*Pseudoperapion brevirostre* (Herbst, 1797)

*Pseudostenapion simum* (Germar, 1817)

Subtribe Aspidapiina Alonso-Zarazaga, 1990

Aspidapion (Aspidapion) radiolus (Marsham, 1802)

Aspidapion (Aspidapion) validum (Germar, 1817)†

Aspidapion (Koestlinia) aeneum (Fabricius, 1775)

Subtribe Catapiina Alonso-Zarazaga,1990

*Catapion seniculus* (Kirby, 1808)

Subtribe Ceratapiina Alonso-Zarazaga, 1990

Ceratapion (Acanephodus) onopordi onopordi (Kirby, 1808)

Ceratapion (Ceratapion) gibbirostre (Gyllenhal, 1813)

Ceratapion (Echinostroma) penetrans penetrans (Germar, 1817) {ZIN}

*Diplapion detritum* (Mulsant & Rey, 1859)

*Omphalapion hookerorum* (Kirby, 1808)

Taphrotopium (Taphrotopium) sulcifrons (Herbst, 1797)

Subtribe Exapiina Alonso-Zarazaga, 1990

*Exapion corniculatum* (Germar, 1817)

Subtribe Kalcapiina Alonso-Zarazaga, 1990

*Kalcapion pallipes* (Kirby, 1808)

Melanapion (Melanapion) minimum (Herbst, 1797)

*Squamapion flavimanum* (Gyllenhal, 1833) {ZIN}

*Squamapion vicinum* (Kirby, 1808)

*Taeniapion urticarium urticarium* (Herbst, 1784)

Subtribe Oxystomatina Alonso-Zarazaga,1990

Cyanapion (Bothryorrhynchapion) gyllenhalii (Kirby, 1808)

Eutrichapion (Eutrichapion) ervi (Kirby, 1808)

Eutrichapion (Eutrichapion) viciae (Paykull, 1800)

Eutrichapion (Psilocalymma) facetum (Gyllenhal, 1839)

Eutrichapion (Psilocalymma) punctiger (Paykull, 1792)

Ischnopterapion (Chlorapion) virens (Herbst, 1797)

Ischnopterapion (Ischnopterapion) loti (Kirby, 1808) {ZIN}

*Oxystoma cerdo* (Gerstaecker, 1854)

*Oxystoma craccae* (Linnaeus, 1767)

*Oxystoma subulatum* (Kirby, 1808)

Synapion (Synapion) ebeninum (Kirby, 1808)

Subtribe Piezotrachelina Voss, 1959

*Protapion apricans* (Herbst, 1797)

*Protapion assimile assimile* (Kirby, 1808)

*Protapion filirostre* (Kirby, 1808)

*Protapion fulvipes fulvipes* (Geoffroy, 1785)

*Protapion interjectum interjectum* (Desbrochers des Loges, 1895)

*Protapion trifolii* (Linnaeus, 1768)

*Protapion varipes* (Germar, 1817)

Subtribe Trichapiina Alonso-Zarazaga,1990

*Betulapion simile simile* (Kirby, 1811)

**Subfamily NANOPHYINAE Gistel, 1848**

**Tribe Nanophyini Gistel, 1856**

*Nanomimus circumscriptus* (Aubé, 1864) {ZIN}

*Nanomimus hemisphaericus* (G.-A. Olivier, 1807)

*Nanophyes brevis brevis* Boheman, 1845

*Nanophyes globiformis* Kiesenwetter, 1864

*Nanophyes globulus* (Germar, 1821)

*Nanophyes marmoratus marmoratus* (Goeze, 1777)

**Family CURCULIONIDAE Latreille, 1802**

**Subfamily BAGOINAE C.G. Thomson, 1859**

Bagous (Bagous) binodulus (Herbst, 1795)

Bagous (Bagous) glabrirostris (Herbst, 1795)

Bagous (Bagous) puncticollis Boheman, 1845

Bagous (Bagous) subcarinatus Gyllenhal, 1836

Bagous (Macropelmus) nodulosus Gyllenhal, 1836

Bagous (Macropelmus) tempestivus (Herbst, 1795)*

**Subfamily BRACHYCERINAE Billberg, 1820**

**Tribe Erirhinini Schoenherr, 1825**

Subtribe Erirhinina Schoenherr, 1825

*Grypus equiseti* (Fabricius, 1775)

*Notaris acridulus* (Linnaeus, 1758)

*Notaris aethiops* (Paykull, 1792)

*Notaris scirpi* (Fabricius, 1792)

*Thryogenes festucae* (Herbst, 1795)

*Thryogenes nereis* (Paykull, 1800)

**Tribe Tanysphyrini Gistel, 1848**

*Tanysphyrus lemnae* (Paykull, 1792)

**Subfamily CONODERINAE Schoenherr, 1833**

**Supertribe Bariditae Schoenherr, 1836**

**Tribe Apostasimerini Schoenherr, 1844**

Subtribe Zygobaridina Pierce, 1907

*Limnobaris dolorosa* (Goeze, 1777)

*Limnobaris t-album* (Linnaeus, 1758)

**Tribe Baridini Schoenherr, 1836**

Subtribe Baridini Schoenherr, 1836

*Baris artemisiae* (Panzer, 1794)

**Supertribe Ceutorhynchitae Gistel, 1848**

**Tribe Amalini Wagner, 19**36

*Amalus scortillum* (Herbst, 1795)

**Tribe Ceutorhynchini Gistel, 1856**

*Calosirus apicalis* (Gyllenhal, 1827) {ZIN}

*Ceutorhynchus contractus* (Marsham, 1802)

*Ceutorhynchus erysimi* (Fabricius, 1787)

*Ceutorhynchus gallorhenanus* F. Solari, 1949

*Ceutorhynchus griseus* C.N.F. Brisout de Barneville, 1869

*Ceutorhynchus hampei* C.N.F. Brisout de Barneville, 1869

*Ceutorhynchus ignitus* Germar, 1823

*Ceutorhynchus pseudoarator* Korotyaev, 1989 {ZIN}

*Ceutorhynchus pulvinatus* Gyllenhal, 1837

*Ceutorhynchus rapae* Gyllenhal, 1837

*Ceutorhynchus roberti* Gyllenhal, 1837

*Ceutorhynchus syrites* Germar, 1823

*Ceutorhynchus typhae* (Herbst, 1795)

*Coeliastes lamii* (Fabricius, 1792) {ZIN}

*Coeliodes rana* (Fabricius, 1787)

*Coeliodinus rubicundus* (Herbst, 1795)

*Datonychus arquata* (Herbst, 1795)

*Datonychus urticae* (Boheman, 1845)

*Glocianus distinctus* (C.N.F. Brisout de Barneville, 1870)

*Glocianus punctiger* (C.R. Sahlberg, 1835)

*Micrelus ericae* (Gyllenhal, 1813) {ZIN}

*Microplontus campestris* (Gyllenhal, 1837) {ZIN}

*Microplontus millefolii* (Schultze, 1897) {ZIN}

*Microplontus triangulum* (Boheman, 1845)

*Mogulones crucifer* (Pallas, 1771)

*Mogulones cynoglossi* (Frauenfeld, 1866)

*Mogulones geographicus* (Goeze, 1777)

*Mogulones pallidicornis* (Gougelet & H. Brisout de Barneville, 1860)

*Nedyus quadrimaculatus* (Linnaeus, 1758)

*Thamiocolus viduatus* (Gyllenhal, 1813)

*Trichosirocalus troglodytes* (Fabricius, 1787)

*Zacladus geranii* (Paykull, 1800)

**Tribe Cnemogonini Colonnelli, 1979**

*Auleutes epilobii* (Paykull, 1800) {ZIN}

**Tribe Mononychini LeConte, 1876**

*Mononychus punctumalbum* (Herbst, 1784)

**Tribe Phytobiini Gistel, 1856**

Marmaropus besseri Gyllenhal, 1837 {ZIN}

*Neophytobius granatus* (Gyllenhal, 1835)

*Neophytobius muricatus* (C.N.F. Brisout de Barneville, 1867)

*Pelenomus commari* (Panzer, 1795)

*Pelenomus waltoni* (Boheman, 1843)

*Rhinoncus bruchoides* (Herbst, 1784)

*Rhinoncus leucostigma* (Marsham, 1802)

*Rhinoncus pericarpius* (Linnaeus, 1758)

*Rhinoncus perpendicularis* (Reich, 1797)

**Tribe Scleropterini Schultze, 1902**

*Rutidosoma graminosum* (Gistel, 1857)

*Tapinotus sellatus* (Fabricius, 1794)

**Supertribe Conoderitae Schoenherr, 1833**

**Tribe Coryssomerini C.G. Thomson, 1859**

*Coryssomerus capucinus* (Beck, 1817)

*Euryommatus mariae* Roger, 1857 {ZIN}

**Supertribe Orobitiditae C.G. Thomson, 1859**

**Tribe Orobitidini C.G. Thomson, 1859**

*Orobitis cyanea* (Linnaeus, 1758)

**Subfamily COSSONINAE Schoenherr, 1825**

**Tribe Cossonini Schoenherr, 1825**

Cossonus (Caenocossonus) parallelepipedus (Herbst, 1795)

**Tribe Rhyncolini Gistel, 1856**

Subtribe Rhyncolina Gistel, 1856

Rhyncolus (Rhyncolus) ater ater (Linnaeus, 1758)

Rhyncolus (Rhyncolus) elongatus (Gyllenhal, 1827)

**Subfamily CURCULIONINAE Latreille, 1802**

**Tribe Acalyptini C.G. Thomson, 1859**

*Acalyptus carpini* (Fabricius, 1792)

*Acalyptus sericeus* Gyllenhal, 1835

**Tribe Anoplini Bedel, 1884**

*Anoplus plantaris* (Næzén, 1794)

**Tribe Anthonomini C.G. Thomson, 1859**

Anthonomus (Anthomorphus) phyllocola (Herbst, 1795)

Anthonomus (Anthomorphus) pinivorax Silfverberg, 1977*

Anthonomus (Anthonomus) conspersus Desbrochers des Loges, 1868

Anthonomus (Anthonomus) incurvus (Panzer, 1795)

Anthonomus (Anthonomus) pomorum (Linnaeus, 1758)

Anthonomus (Anthonomus) rubi (Herbst, 1795)

Anthonomus (Anthonomus) sorbi Germar, 1821

Anthonomus (Anthonomus) ulmi (De Geer, 1775) {ZIN}

Anthonomus (Furcipus) rectirostris (Linnaeus, 1758)

Bradybatus (Bradybatus) kellneri Bach, 1854

**Tribe Cionini Schoenherr, 1825**

*Cionus hortulanus* (Geoffroy, 1785)

? *Cionus olivieri* Rosenschoeld, 1838 {Feoktistov 2011}

*Cionus scrophulariae* (Linnaeus, 1758)

*Cionus tuberculosus* (Scopoli, 1763)

**Tribe Curculionini Latreille, 1802**

Subtribe Archariina Pelsue & O’Brien, 2011

Archarius (Archarius) pyrrhoceras (Marsham, 1802)

Archarius (Archarius) salicivorus (Paykull, 1792)

Subtribe Curculionina Latreille, 1802

Curculio (Curculio) glandium Marsham, 1802

Curculio (Curculio) nucum Linnaeus, 1758

Curculio (Curculio) rubidus (Gyllenhal, 1835)

**Tribe Ellescini C.G. Thomson, 1859**

Subtribe Dorytomina Bedel, 1886

Dorytomus (Dorytomus) salicinus (Gyllenhal, 1827)

Dorytomus (Dorytomus) taeniatus (Fabricius, 1781)

Dorytomus (Dorytomus) tortrix (Linnaeus, 1760)

Dorytomus (Dorytomus) tremulae (Fabricius, 1787)

Subtribe Ellescina C.G. Thomson, 1859

*Ellescus bipunctatus* (Linnaeus, 1758)

*Ellescus infirmus* (Herbst, 1795)

*Ellescus scanicus* (Paykull, 1792)

**Tribe Mecinini Gistel, 1848**

*Cleopomiarus distinctus* (Boheman, 1845)

*Cleopomiarus graminis* (Gyllenhal, 1813)

*Gymnetron melanarium* (Germar, 1821)

*Gymnetron terminassianae* Smreczyński, 1975 {ZIN}

*Gymnetron veronicae* (Germar, 1821)

*Mecinus heydenii* Wencker, 1866

*Mecinus janthinus* Germar, 1821

*Mecinus labilis* (Herbst, 1795)

*Mecinus pascuorum* (Gyllenhal, 1813)

*Mecinus plantaginis* (Eppelsheim, 1875)

*Mecinus pyraster* (Herbst, 1795)

*Miarus ajugae* (Herbst, 1795)

*Rhinusa antirrhini* (Paykull, 1800)

*Rhinusa asellus* (Gravenhorst, 1807)

*Rhinusa collina* (Gyllenhal, 1813)

*Rhinusa linariae* (Panzer, 1795)

*Rhinusa neta* (Germar, 1821)

**Tribe Rhamphini Rafinesque, 1815**

Subtribe Rhamphina Rafinesque, 1815

*Isochnus foliorum* (O.F. Müller, 1764)

*Isochnus sequensi* (Stierlin, 1894)

Orchestes (Alyctus) calceatus (Germar, 1821)

Orchestes (Alyctus) rusci (Herbst, 1795)

Orchestes (Orchestes) hortorum (Fabricius, 1792)

*Pseudorchestes circumvistulanus* (Białooki, 1997) {ZIN}

*Pseudorchestes pratensis* (Germar, 1821)

*Rhamphus pulicarius* (Herbst, 1795)

Rhynchaenus (Rhynchaenus) xylostei Clairville, 1798

?*Tachyerges rufitarsis* (Germar, 1821) {Feoktistov 2011}

*Tachyerges salicis* (Linnaeus, 1758)

*Tachyerges stigma* (Germar, 1821)

**Tribe Smicronychini Seidlitz, 1891**

Smicronyx (Smicronyx) coecus (Reich, 1797)

Smicronyx (Smicronyx) smreczynskii F. Solari, 1952

**Tribe Tychiini C.G. Thomson, 1859**

Subtribe Tychiina C.G. Thomson, 1859

Sibinia (Sibinia) pellucens (Scopoli, 1772)

Sibinia (Sibinia) subelliptica (Desbrochers des Loges, 1873)

Sibinia (Sibinia) tibialis Gyllenhal, 1835

Sibinia (Sibinia) viscariae (Linnaeus, 1760)

Tychius (Tychius) medicaginis C.N.F. Brisout de Barneville, 1863

Tychius (Tychius) picirostris (Fabricius, 1787)

Tychius (Tychius) quinquepunctatus (Linnaeus, 1758)

Tychius (Tychius) stephensi Schoenherr, 1835

**Subfamily DRYOPHTHORINAE Schoenherr, 1825**

**Tribe Rhynchophorini Schoenherr, 1833**

Subtribe Litosomina Lacordaire, 1865

*Sitophilus granarius* (Linnaeus, 1758)†

Subtribe Sphenophorina Lacordaire, 1865

*Sphenophorus striatopunctatus* (Goeze, 1777)

**Subfamily ENTIMINAE Schoenherr, 1823**

**Tribe Brachyderini Schoenherr, 1826**

Brachyderes (Brachyderes) incanus (Linnaeus, 1758)

Strophosoma (Strophosoma) capitatum (De Geer, 1775)

**Tribe Cneorhinini Lacordaire, 1863**

*Attactagenus albinus* (Boheman, 1833)

**Tribe Otiorhynchini Schoenherr, 1826**

Otiorhynchus (Choilisanus) raucus (Fabricius, 1777)

*Otiorhynchus* (*Cryphiphorus) ligustici* (Linnaeus, 1758)

Otiorhynchus (Otiolehus) tristis (Scopoli, 1763)

Otiorhynchus (Pendragon) ovatus ovatus (Linnaeus, 1758)

**Tribe Phyllobiini Schoenherr, 1826**

Phyllobius (Alsus) brevis Gyllenhal, 1834

Phyllobius (Dieletus) argentatus argentatus (Linnaeus, 1758)

Phyllobius (Metaphyllobius) jacobsoni Smirnov, 1913

Phyllobius (Metaphyllobius) pomaceus Gyllenhal, 1834

Phyllobius (Nemoicus) oblongus (Linnaeus, 1758)

Phyllobius (Phyllobius) arborator (Herbst, 1797)

Phyllobius (Phyllobius) pyri (Linnaeus, 1758)

Phyllobius (Phyllobius) thalassinus Gyllenhal, 1834

Phyllobius (Pterygorrhynchus) maculicornis Germar, 1823

**Tribe Polydrusini Schoenherr, 1823**

Liophloeus (Liophloeus) tessulatus (O.F. Müller, 1776)

Polydrusus (Eudipnus) mollis (Strøm, 1768)

Polydrusus (Eurodrusus) cervinus (Linnaeus, 1758)

Polydrusus (Eurodrusus) confluens Stephens, 1831

Polydrusus (Eustolus) flavipes flavipes (De Geer, 1775)

Polydrusus (Eustolus) pterygomalis Boheman, 1840

Polydrusus (Polydrusus) fulvicornis fulvicornis (Fabricius, 1792)

Polydrusus (Polydrusus) tereticollis (De Geer, 1775)

**Tribe Sciaphilini Sharp, 1891**

Brachysomus (Brachysomus) echinatus (Bonsdorff, 1785)

*Eusomus ovulum* Germar, 1823

*Exomias lebedevi* (Roubal, 1926)

*Sciaphilus asperatus* (Bonsdorff, 1785)

**Tribe Sitonini Gistel, 1848**

*Charagmus griseus* (Fabricius, 1775)

*Sitona ambiguus* Gyllenhal, 1834

*Sitona cylindricollis cylindricollis* Fåhraeus, 1840

*Sitona hispidulus* (Fabricius, 1777)

*Sitona inops* Schoenherr, 1832

*Sitona lineatus* (Linnaeus, 1758)

*Sitona longulus* Gyllenhal, 1834

*Sitona macularius macularius* (Marsham, 1802)

*Sitona obsoletus obsoletus* (Gmelin, 1790)

*Sitona puncticollis* Stephens, 1831

*Sitona striatellus* Gyllenhal, 1834

*Sitona sulcifrons sulcifrons* (Thunberg, 1798)

*Sitona suturalis* Stephens, 1831

**Tribe Tanymecini Lacordaire, 1863**

Subtribe Tanymecina Lacordaire, 1863

*Chlorophanus viridis viridis* (Linnaeus, 1758)

Tanymecus (Tanymecus) palliatus (Fabricius, 1787)

**Tribe Trachyphloeini Gistel, 1848**

*Romualdius scaber* (Linnaeus, 1758)

**Subfamily HYPERINAE Lacordaire, 1863 (1848)**

**Tribe Hyperini Lacordaire, 1863 (1848)**

Hypera (Boreohypera) diversipunctata (Schrank, 1798)

Hypera (Boreohypera) fornicata (Penecke, 1928)

Hypera (Dapalinus) meles (Fabricius, 1792)

Hypera (Eririnomorphus) conmaculata (Herbst, 1795)

Hypera (Eririnomorphus) rumicis (Linnaeus, 1758)

Hypera (Hypera) miles (Paykull, 1792)

Hypera (Hypera) postica (Gyllenhal, 1813)

Hypera (Hypera) transsilvanica (Petri, 1901)

Hypera (Hypera) viciae (Gyllenhal, 1813)

Hypera (Kippenbergia) arator (Linnaeus, 1758)

*Limobius borealis* (Paykull, 1792)

**Subfamily LIXINAE Schoenherr, 1823**

**Tribe Cleonini Schoenherr, 1826**

*Asproparthenis foveocollis* (Gebler, 1834)

*Bothynoderes affinis* (Schrank, 1781)

*Cleonis pigra* (Scopoli, 1763)

Coniocleonus (Augustecleonus) hollbergii (Fåhraeus, 1842)

*Cyphocleonus dealbatus* (Gmelin, 1790)

*Cyphocleonus trisulcatus* (Herbst, 1795)

**Tribe Lixini Schoenherr, 1823**

Larinus (Larinomesius) obtusus Gyllenhal, 1835

Larinus (Phyllonomeus) planus (Fabricius, 1792)

Larinus (Phyllonomeus) sturnus (Schaller, 1783)

Larinus (Phyllonomeus) turbinatus Gyllenhal, 1835

Lixus (Dilixellus) bardanae (Fabricius, 1787)

Lixus (Dilixellus) fasciculatus Boheman, 1835

Lixus (Dilixellus) pulverulentus (Scopoli, 1763)

Lixus (Epimeces) filiformis (Fabricius, 1781)

Lixus (Eulixus) iridis G.-A. Olivier, 1807

Lixus (Eulixus) myagri G.-A. Olivier, 1807

Lixus (Lixus) paraplecticus (Linnaeus, 1758)

Lixus (Phillixus) brevipes C.N.F. Brisout de Barneville, 1866 {ZIN}

**Subfamily MESOPTILIINAE Lacordaire, 1863**

**Tribe Magdalidini Pascoe, 1870**

Magdalis (Edo) ruficornis (Linnaeus, 1758)

Magdalis (Magdalis) duplicata Germar, 1819

Magdalis (Magdalis) frontalis (Gyllenhal, 1827)

Magdalis (Magdalis) linearis (Gyllenhal, 1827)

Magdalis (Magdalis) phlegmatica (Herbst, 1797)

Magdalis (Magdalis) violacea (Linnaeus, 1758)

Magdalis (Odontomagdalis) armigera (Geoffroy, 1785)

**Subfamily MOLYTINAE Schoenherr, 1823**

**Tribe Cryptorhynchini Schoenherr, 1825**

Subtribe Cryptorhynchina Schoenherr, 1825

*Cryptorhynchus lapathi* (Linnaeus, 1758)

Subtribe Tylodina Lacordaire, 1865

*Acalles echinatus* (Germar, 1823)

**Tribe Molytini Schoenherr, 1823**

Subtribe Hylobiina Kirby, 1837

? Hylobius (Hylobius) excavatus (Laicharting, 1781) {Feoktistov 2011}

Hylobius (Callirus) abietis (Linnaeus, 1758)

Hylobius (Callirus) pinastri (Gyllenhal, 1813)

**Tribe Pissodini Gistel, 1848**

Subtribe Pissodina Gistel, 1848

Pissodes (Pissodes) castaneus (De Geer, 1775)

Pissodes (Pissodes) harcyniae (Herbst, 1795) {ZIN}

Pissodes (Pissodes) pini pini (Linnaeus, 1758)

Pissodes (Pissodes) piniphilus (Herbst, 1797)

Pissodes (Pissodes) validirostris (C.R. Sahlberg, 1834)

**Tribe Trachodini Gistel, 1848**

*Trachodes hispidus* (Linnaeus, 1758)

**Subfamily SCOLYTINAE Latreille, 1804**

**Tribe Corthylini LeConte, 1876**

Subtribe Pityophthorina Eichhoff, 1878

*Pityophthorus glabratus* Eichhoff, 1878 {ZIN}

*Pityophthorus lichtensteinii* (Ratzeburg, 1837)

*Pityophthorus micrographus micrographus* (Linnaeus, 1758) {ZIN}

*Pityophthorus traegardhi* Spessivtsev, 1921 {ZIN}

**Tribe Cryphalini Lindemann, 1877**

*Ernoporus tiliae* (Panzer, 1793) {ZIN}

*Trypophloeus binodulus* (Ratzeburg, 1837) {ZIN}

*Trypophloeus discedens* Palm, 1950 {ZIN}

**Tribe Crypturgini LeConte, 1876**

*Crypturgus cinereus* (Herbst, 1793) {ZIN}

*Crypturgus hispidulus* C.G. Thomson, 1870 {ZIN}

*Crypturgus pusillus* (Gyllenhal, 1813) {ZIN}

*Crypturgus subcribrosus* Eggers, 1933 {ZIN}

**Tribe Dryocoetini Lindemann, 1877**

*Dryocoetes autographus* (Ratzeburg, 1837) {ZIN}

*Dryocoetes hectographus* Reitter, 1913 {ZIN}

*Lymantor aceris aceris* (Lindemann, 1875) {ZIN}

*Lymantor coryli* (Perris, 1855) {ZIN}

**Tribe Hylastini LeConte, 1876**

*Hylastes angustatus* (Herbst, 1793)

*Hylastes ater* (Paykull, 1800) {ZIN}

*Hylastes brunneus* (Erichson, 1836) {ZIN}

*Hylastes cunicularius* Erichson, 1836 {ZIN}

*Hylastes opacus* Erichson, 1836

*Hylurgops palliatus* (Gyllenhal, 1813) {ZIN}

**Tribe Hylurgini Gistel, 1848**

*Dendroctonus micans* (Kugelann, 1794)

*Hylurgus ligniperda* (Fabricius, 1787) {ZIN}

*Tomicus minor* (Hartig, 1834)

*Tomicus piniperda* (Linnaeus, 1758) {ZIN}

**Tribe Ipini Bedel, 1888**

*Ips acuminatus* (Gyllenhal, 1827) {ZIN}

*Ips duplicatus* (C.R. Sahlberg, 1836)

*Ips sexdentatus* (Boerner, 1776) {ZIN}

*Ips typographus* (Linnaeus, 1758) {ZIN}

*Orthotomicus laricis* (Fabricius, 1792) {ZIN}

*Orthotomicus longicollis* (Gyllenhal, 1827)

*Orthotomicus proximus* (Eichhoff, 1868)

*Orthotomicus starki* Spessivtsev, 1926 {ZIN}

*Orthotomicus suturalis* (Gyllenhal, 1827)

*Pityogenes bidentatus* (Herbst, 1783)

*Pityogenes chalcographus* (Linnaeus, 1760) {ZIN}

*Pityogenes irkutensis irkutensis* Eggers, 1910

*Pityogenes quadridens* (Hartig, 1834)

**Tribe Polygraphini Chapuis, 1869**

*Carphoborus rossicus* Semenov, 1902 {ZIN}

*Polygraphus poligraphus* (Linnaeus, 1758) {ZIN}

*Polygraphus subopacus* C.G. Thomson, 1871 {ZIN}

**Tribe Scolytini Latreille, 1804**

*Scolytus intricatus* (Ratzeburg, 1837) {ZIN}

*Scolytus laevis* Chapuis, 1869 {ZIN}

*Scolytus multistriatus* (Marsham, 1802) {ZIN}

*Scolytus ratzeburgii* E.W. Janson, 1856 {ZIN}

*Scolytus rugulosus* (P.W.J. Müller, 1818) {ZIN}

*Scolytus scolytus* (Fabricius, 1775)

**Tribe Xyleborini LeConte, 1876**

*Anisandrus dispar* (Fabricius, 1792) {ZIN}

*Xyleborinus saxesenii* (Ratzeburg, 1837) {ZIN}

*Xyleborus cryptographus* (Ratzeburg, 1837) {ZIN}

**Tribe Xyloterini LeConte, 1876**

*Trypodendron laeve* Eggers, 1939 {ZIN}

*Trypodendron lineatum* (G.-A. Olivier, 1800) {ZIN}

*Trypodendron signatum* (Fabricius, 1792) {ZIN}

## Notes

This checklist includes data on 2145 species from 88 families (Table 1); the Ptiliidae and Clambidae collected in the Mordovia Nature Reserve remain to be identified. The occurrences of Spercheidae, Psephenidae, Drilidae, and Stenotrachelidae in the reserve is possible but not yet confirmed.

The most diverse families (Carabidae, Staphylinidae, Cerambycidae, Chrysomelidae and Curculionidae) make up a total of 57.6% of the Coleoptera diversity of the Reserve. Forty-seven species from 20 families are listed for the first time for the Mordovia State Nature Reserve and the Republic of Mordovia. Detailed information about them will be published separately.

**Table 1.** Coleoptera species richness by family and number of adventive species in Mordovia State Nature Reserve, Russia.

Taxon names	No. of species	Adventive species
** Myxophaga **		
Sphaeriusidae	1	
** Adephaga **		
Gyrinidae	5	
Carabidae	231	
Haliplidae	4	
Noteridae	2	
Dytiscidae	71	
** Polyphaga **		
** Hydrophiloidea **		
Helophoridae	1	
Georissidae	1	
Hydrochidae	4	
Hydrophilidae	38	1
Sphaeritidae	1	
Histeridae	38	
** Staphylinoidea **		
Hydraenidae	1	
Leiodidae	18	
Silphidae	16	
Staphylinidae	436	3
Scydmaenidae	12	
** Scarabaeoidea **		
Geotrupidae	3	
Trogidae	3	
Lucanidae	4	
Scarabaeidae	60	
** Scirtoidea **		
Scirtidae	6	
Eucinetidae	1	
** Dascilloidea **		
Dascillidae	1	
** Buprestoidea **		
Buprestidae	27	
** Byrrhoidea **		
Byrrhidae	7	
Elmidae	1	
Dryopidae	2	
Limnichidae	1	
Heteroceridae	5	
** Elateroidea **		
Eucnemidae	13	
Throscidae	1	
Elateridae	59	
Lycidae	8	
Lampyridae	1	
Cantharidae	26	
** Bostrichoidea **		
Dermestidae	15	4
Bostrichidae	2	
Ptinidae	19	2
** Lymexyloidea **		
Lymexylidae	2	
** Cleroidea **		
Biphyllidae	2	
Byturidae	2	
Trogossitidae	4	
Cleridae	6	1
Melyridae	15	
** Cucujoidea **		
Sphindidae	2	
Erotylidae	10	
Monotomidae	11	
Kateretidae	7	
Nitidulidae	33	4
Cryptophagidae	14	2
Silvanidae	6	
Cucujidae	4	
Phalacridae	4	
Laemophloeidae	6	
** Coccinelloidea **		
Bothrideridae	1	
Cerylonidae	5	
Latridiidae	20	2
Corylophidae	4	
Anamorphidae	1	
Endomychidae	5	
Coccinellidae	43	1
** Tenebrionoidea **		
Mycetophagidae	11	1
Ciidae	11	
Tetratomidae	3	
Melandryidae	16	
Ripiphoridae	2	
Zopheridae	4	
Mordellidae	23	
Tenebrionidae	37	3
Oedemeridae	7	
Meloidae	10	
Boridae	1	
Pythidae	1	
Pyrochroidae	2	
Salpingidae	5	
Anthicidae	5	1
Aderidae	4	
Scraptiidae	5	
** Chrysomeloidea **		
Cerambycidae	98	1
Megalopodidae	2	
Orsodacnidae	1	
Chrysomelidae	188	2
** Curculionoidea **		
Nemonychidae	1	
Anthribidae	8	
Attelabidae	14	1
Brentidae	52	1
Curculionidae	282	1
**Total**	**2145**	**31**

## Discussion

The Mordovia State Nature Reserve is a unique refugium of forest that has been little affected by human activity for many centuries (Ruchin and Khapugin 2019). This has preserved very rare species that are known from single records in the center of the European part of Russia: *Ilybius wasastjernae* (Dytiscidae), *Aleochara falcata*, *Alevonota egregia*, *Atheta sequanica*, *Bledius fergussoni*, *Gyrophaena nitidula* and *Sepedophilus binotatus* (Staphylinidae), *Agrilus kaluganus* (Buprestidae), *Isorhipis melasoides* (Eucnemidae), *Denticollis rubens* and *Ampedus nigerrimus* (Elateridae), *Erotides nasutus* and *Lopheros lineatus* (Lycidae), *Allonyx quadrimaculatus* (Cleridae), *Ipidia sexguttata* (Nitidulidae), *Cucujus cinnaberinus* (Cucujidae), *Clemmus troglodytes* (Anamorphidae), *Phryganophilus pseudauritus* (Melandryidae), *Diaclina fagi* (Tenebrionidae), *Leptura aurulenta*, *Phymatodes abietinus* and *Purpuricenus globulicollis* (Cerambycidae), *Syneta betulae* (Chrysomelidae), *Mesauletobius pubescens* (Rhynchitidae), *Ceutorhynchus pseudoarator*, *Euryommatus mariae* and *Anthonomus ulmi* (Curculionidae).

The Mordovia State Nature Reserve is important for the conservation of rare Coleoptera species. It is home to eight species listed in the Red book of the Russian Federation (Ruchin and Kurmaeva 2010, Ruchin and Egorov 2017b, Ruchin and Khapugin 2019, Egorov and Ruchin 2020): *Dytiscus latissimus*, *Trypocopris vernalis*, *Ceruchus chrysomelinus*, *Osmoderma barnabita*, *Protaetia speciosissima*, *Protaetia fieberi*, *Elater ferrugineus* and *Melandrya barbata*. *Trypocopris vernalis*, *Elater ferrugineus* and *Melandrya barbata* are only found only in the territory of the Mordovia State Nature Reserve within the Republic of Mordovia.

The Coleoptera fauna contains 31 adventive species as currently known (1.44% of beetle species diversity) from 17 families (Table 1). The largest number of adventive species has been recorded in the families Staphylinidae (4 species), Dermestidae, Nitidulidae and Tenebrionidae (3 species each). The small proportion of adventive species in the fauna possibly indicates the stability of the ecosystems of the Mordovia State Nature Reserve and the weak anthropogenic impact on them.

The obtained results on the diversity of beetles in the Mordovia State Nature Reserve can be compared with similar data from other protected areas with well-studied Coleoptera both in Russia and in other countries (see Table 2).

Analysis of the data on the degree of study of the beetle fauna in natural protected areas of the European part of Russia allows us to conclude that the beetle fauna of the Mordovian State Nature Reserve is the most studied.

The study of the beetle fauna of the Mordovia State Nature Reserve needs to be continued. The families Helophoridae, Hydraenidae, Leiodidae, Elmidae, Throscidae, Cryptophagidae, Phalacridae, and Scraptiidae have not been sufficiently studied and require particular attention.

**Table 2.** Comparative Coleoptera species richness in some protected areas of Russia and other countries.

**Name of the protected area**	**Country**	**Number of species**	**Area, km^2^**	**Source of information**
Mordovia State Nature Reserve	Russia	2145	321	Our data
Lasovsky Nature Reserve	Russia	2183	1210	Storozhenko et al. 2009
Oka State Nature Biosphere Reserve	Russia	1377	558	Priklonsky et al. 2001, Nikolaeva et al. 2015
Meshchera National Park	Russia	1390	1189	Semenov 2009
National Park “Smolensk Lakeland”	Russia	1526	1462	Semenov et al. 2011
National Park “Belovezhskaya pushcha”	Belarus	2101	870	Tsinkevich 2017
Białowieża National Park	Poland	2973	630	Plewa et al. 2020
Gauja National Park	Latvia	1583	917	Kalniņš et al. 2007
New Forest National Park	England	2600	571	https://www.newforestnpa.gov.uk/discover/wildlife/beetles/
Great Smoky Mountains National Park	USA	2522	2108	Carlton 2013
